# The phenotypic landscape of the model firmicute *Bacillus subtilis*

**DOI:** 10.1073/pnas.2619440123

**Published:** 2026-09-29

**Authors:** Lili M. Kim, Byoung-Mo Koo, Hannah N. Burkhart, Daniela Vollmer, Waldemar Vollmer, Horia Todor, Carol A. Gross

**Affiliations:** ^a^ Department of Microbiology and Immunology, University of California, San Francisco, CA 94158; ^b^ Institute for Molecular Bioscience, The University of Queensland, Brisbane 4067, QLD, Australia; ^c^ Department of Cell and Tissue Biology, University of California, San Francisco, CA 94158; ^d^ Quantitative Biosciences Institute, University of California, San Francisco, CA 94158

**Keywords:** *Bacillus subtilis*, chemical genomics, firmicutes

## Abstract

Firmicutes are gram-positive bacteria with important roles in human health, disease, and industry. However, more than a quarter of genes in the model firmicute *Bacillus subtilis* remain completely uncharacterized, including numerous phylum-specific genes. Here, we design a compact pooled CRISPRi library targeting all protein-coding genes in *B. subtilis* and test its growth in ~150 stress conditions. Using data from this screen as a hypothesis generator, we perform targeted experiments, revealing that the conserved essential firmicute protein YneF is part of the SRP cotranslational protein secretion pathway. We also demonstrate that ECF-transporters play a broadly conserved role in cell wall homeostasis, perform an unbiased analysis of amino acid crossfeeding, and make additional insights into bacterial competition. This work additionally provides a rich dataset to nucleate future studies of uncharacterized genes and presents a framework for performing accessible full-genome functional genomic screens in other bacteria.

## Significance

Large-scale chemical genomics screens facilitate the characterization of genes by generating phenotypic data across a library of gene mutants. Here, using CRISPRi, we designed a compact pooled library targeting all genes in *B. subtilis* and screened growth of the library in ~150 conditions. Importantly, we used this dataset to expand *B. subtilis* biology on levels ranging from molecular pathways to bacterial communities. We discovered a role for an essential firmicute gene in protein secretion, implicated two uncharacterized genes in cell wall homeostasis, and unraveled fundamental factors driving competition and cooperation. This rich dataset will serve as a hypothesis generator, expanding our set of bacterial phenotypic data and driving future experiments in *B. subtilis* and other gram-positive firmicutes.

*Bacillus subtilis* is an important microbe for biomedical research and industrial applications. It is the model organism for gram-positive firmicutes, which include numerous pathogens (e.g., *Staphylococcus aureus*, *Streptococcus pneumoniae*, *Clostridium difficile*) and commensals (e.g., *Holdemanella biformis, Ruminococcus bromii, *Lactobacillus* plantarum*), boasts powerful tools for genetic and molecular research (1–6), and is widely recognized as a workhorse in biotechnology (7, 8). However, despite being one of the most intensively studied organisms, the function of ~25% of all *B. subtilis* genes remains uncharacterized or poorly understood (9). Such a gap can be addressed by large-scale chemical genomics screens, which measure the fitness of each strain in a genome-wide mutant library in a wide range of stress conditions. These screens identify genes required in specific conditions, and, importantly, associate genes of unknown function to known pathways on the basis of correlated phenotypes across conditions [phenotypic signatures; (10)]. Chemical-genomics screens have been previously performed in other firmicutes; however, these have been technically limited. Large genome-wide screens in *S. aureus* (11) and *S. pneumoniae* (12) used TnSeq (13, 14), which cannot query essential gene phenotypes and is limited by sequencing depth. Conversely, small scale CRISPRi screens in *B. subtilis* and *S. pneumoniae* targeted only essential genes and used an arrayed approach (4, 15). Finally, all of these screens used a small selection of (primarily) antibiotic stressors (20 to 32 conditions), which limits the interpretability of correlated phenotypic signatures.

Here, we overcome these limitations and perform a comprehensive chemical genomics screen in *B. subtilis* using a pooled CRISPRi library targeting all protein-coding genes. Our library combines full knockdown of nonessential genes (approximately equivalent to their deletion) with graded partial knockdown of essential genes using mismatch-CRISPRi to titrate sgRNA efficacy (16), which allows probing their mutant phenotypes without cell death. By designing our library to maximize phenotypic data while minimizing library size, we facilitate screening and enable extensive multiplexing, reducing experimental effort and sequencing cost. We subjected our library to an extensive array of conditions (n = 149), which included antibiotics targeting all essential processes as well as environmental stressors such as pH, osmotic stress, and nonantibiotic chemical perturbations. Our final dataset was highly reproducible and yielded thousands of phenotypes and gene–gene correlations, which 1) revealed the role of the uncharacterized essential gene *yneF*; 2) discovered narrowly and broadly conserved players in cell wall homeostasis; and 3) provided systems-level overviews of inter- and intraspecies interactions and crossfeeding. We present this robust dataset as a powerful new resource for understanding firmicute biology and as a framework for accessible full-genome functional genomic screens in other organisms.

## Results and Discussion

### A Genome-Wide Pooled CRISPRi Screen in *B. subtilis*.

**Fig. 1.**
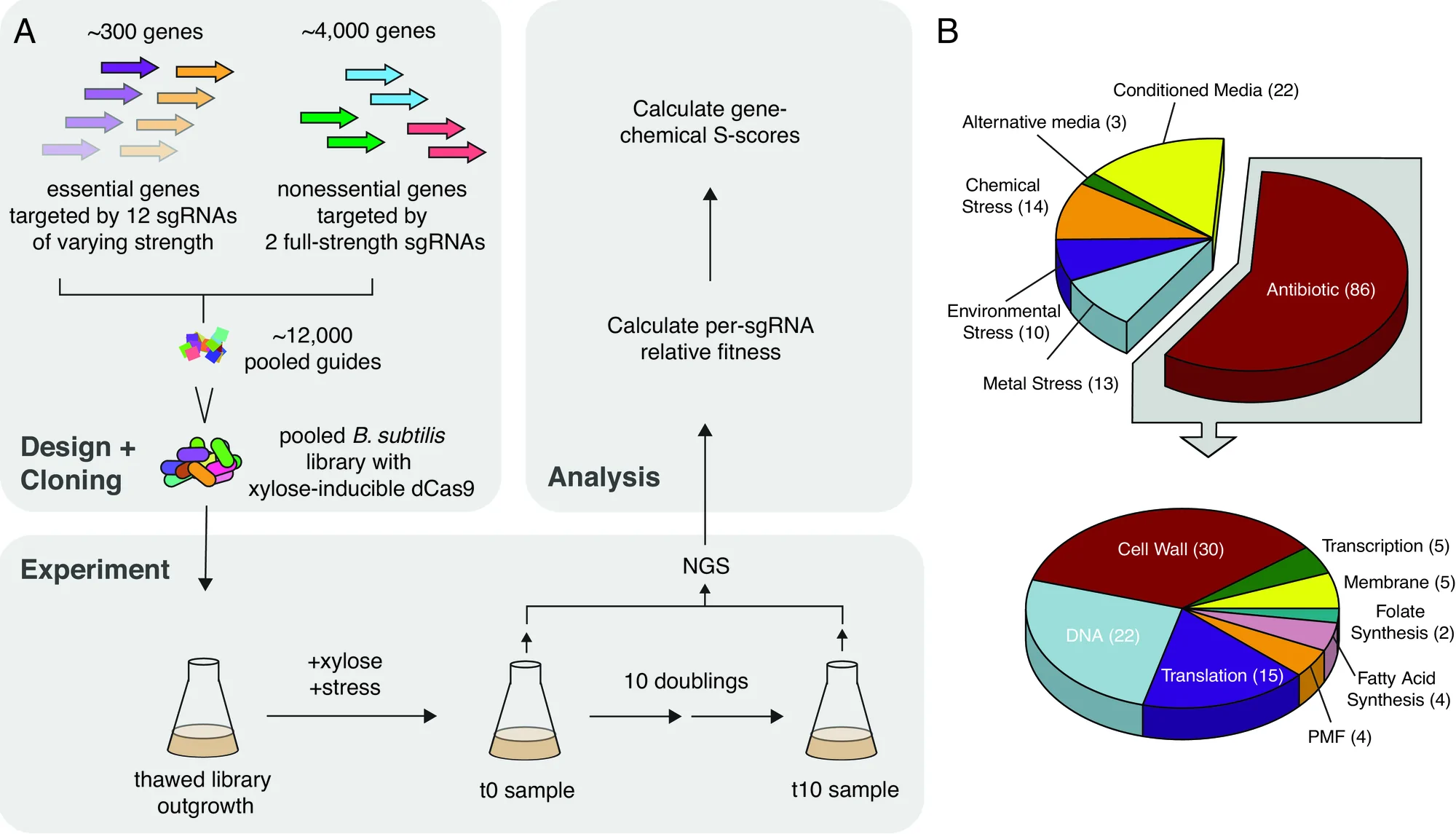
Design of full-genome CRISPRi chemical genomics screen. (*A*) Strategy for design (*Top Left*), experimental setup (*Bottom*), and analysis (*Top Right*) of an 11,559-sgRNA CRISPRi library targeting the full *B. subtilis* genome. Full details provided in *Methods*. (*B*) Categories of stress conditions tested (*Top*) and subcategories of antibiotics tested (*Bottom*). When possible, each stressor was tested at two sub-MIC concentrations (~10% and 25% growth inhibition); each concentration of each stressor is counted.

Our pooled CRISPRi library targeted all protein-coding genes in *B. subtilis* using a previously described and well-established inducible CRISPRi system (4, 5, 16). Both sgRNAs and dCas9 are integrated into the chromosome; sgRNAs are constitutively expressed under *P_veg_* while dCas9 is xylose-inducible under *P_xyl_* (*Methods* and Dataset S1). Nonessential genes (n = 3,981 genes) were fully knocked-down by two fully complementary, computationally optimized sgRNAs [*Methods*; (17)], while essential genes and those with strong growth defects [n = 307 genes; (5)] were targeted by 12 sgRNAs spanning the full range of possible knockdown levels explored in a previous study [(16); Fig. 1*A*; Dataset S2 and *Methods*]. This pooled, compact library (n = 11,559 sgRNAs) is easy to handle and multiplex, facilitating screening in a large number of conditions.

We simultaneously subjected our exponentially growing pooled CRISPRi knockdown library to dCas9 induction and a stress condition. Our 83 unique stressors target all major processes in *B. subtilis* and include antibiotics (e.g., β-lactams, macrolides, fluoroquinolones), antimicrobials (e.g., ethidium bromide, NaCl, SDS), metals (e.g., ZnCl_2_, MnCl_2_, CuCl_2_), diverse environments, and others (Fig. 1*B* and Dataset S1). Where possible, we used two sub-MIC concentrations of each stressor (~10% and ~25% growth inhibition) to maximize our ability to accurately measure both resistance and susceptibility (149 conditions total). Cultures were sampled for next generation sequencing immediately before dCas9 induction and after 10 doublings post induction, as previously described (16, 18–20). We calculated the relative fitness (RF) of each strain in each condition by comparing its relative abundance before and after the experiment (Fig. 1*A* and Dataset S3), as previously established for pooled bacterial mutant screens (13). RF values were consistent across replicates (*r* > 0.95; *SI Appendix*, Fig. S1 *A* and *B*), and with previously published CRISPRi and knockout studies [*r* > 0.95; (5, 16), *SI Appendix*, Fig. S1 *C* and *D* and *Methods*].

Knockdown of a gene by CRISPRi can affect the expression of other genes in the operon (4, 21, 22). To quantify the prevalence of these “polar effects”, we focused on nonessential genes sharing an operon with essential genes. We reasoned that such nonessential genes exhibiting reduced (e.g., RF < 0.9) fitness in the absence of stress would indicate a polar effect on the coexpressed essential gene. 43% such sgRNAs had an RF < 0.9 in LB (183/423 sgRNAs), suggesting that polar effects are common but not universal (*SI Appendix*, Fig. S1*D*). To mitigate these effects, we used single-gene deletion strains (5) for all follow-up experiments.

**Fig. 2.**
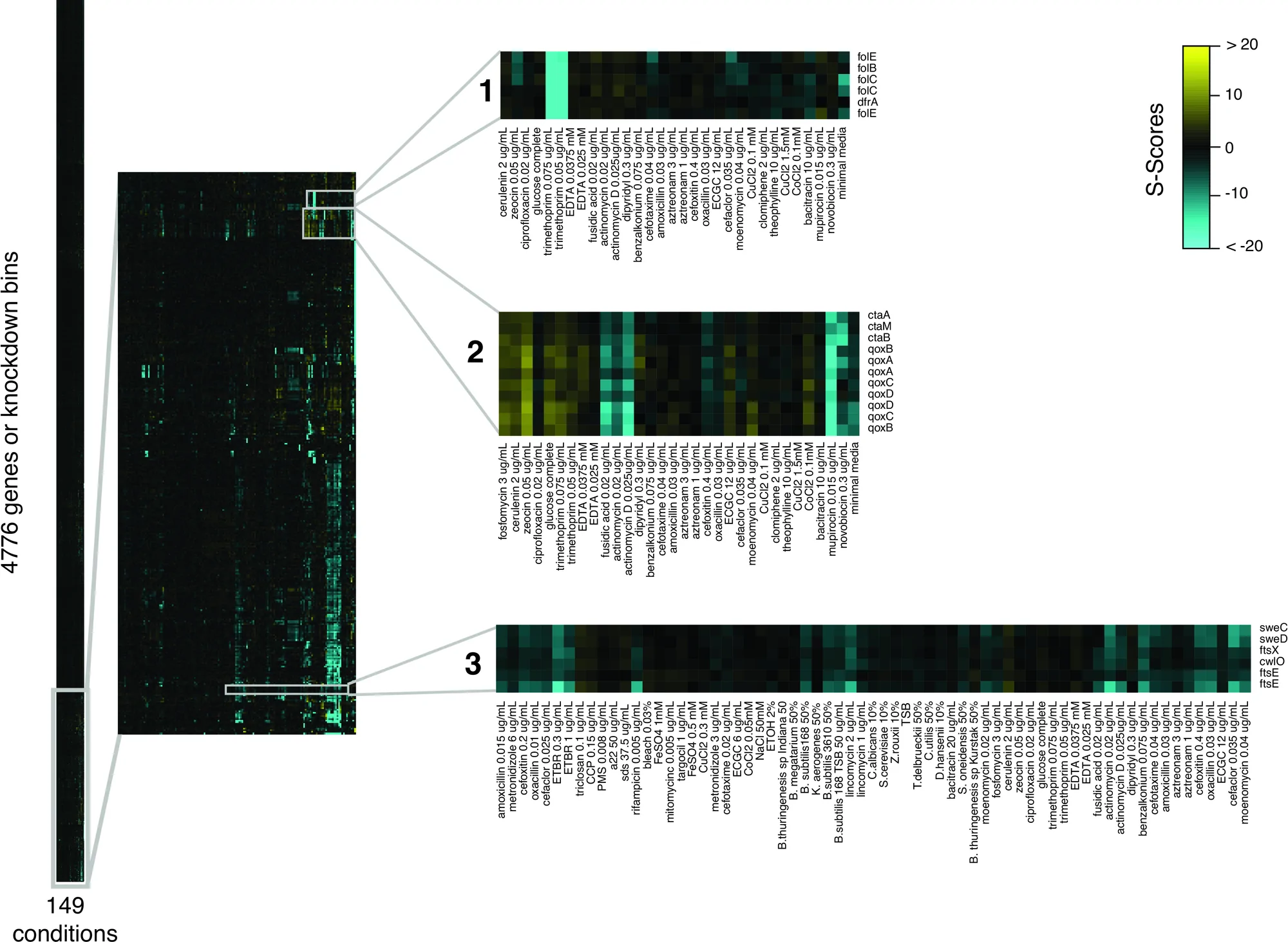
Clustered heatmap of fitness scores for 4776 nonessential genes or binned knockdowns of essential- or semiessential genes in response to all 149 conditions. Zoomed insets demonstrate coclustering of conditions (x-axis) and genes (y-axis): 1. *dfrA* and *folBCE*, essential biosynthesis genes of folate, cocluster due to shared sensitivity to trimethoprim, which targets DfrA. 2. *qoxABCD,* the cytochrome aa3 oxidase, *ctaAB,* synthesis genes of the heme cofactor used by the Qox oxidase, and *ctaM,* required for Qox assembly and function (28), cluster together due to diverse positive (e.g., zeocin) and negative (e.g., mupirocin) phenotypes. 3. *cwlO,* involved in cell elongation, *ftsEX,* required for CwlO function, and *sweCD*, cofactors of FtsEX (29), cluster together due to shared sensitivity to cell-wall drugs. Essential genes appear multiple times in the heatmap because they have three separate sets of S-scores for low, medium, and high knockdown efficiency bins. The three bins may not always cluster together; high-knockdown bins are less likely to show specific negative phenotypes (as cells are already sick due to the strong knockdown of an essential gene), while low-knockdown bins are less likely to show specific positive phenotypes (as these mutants often grow similarly to WT).

We converted our RF values to chemical–gene interaction scores [S-scores; *Methods*; (23)]. For each gene–chemical interaction (e.g., *pbpA* in amoxicillin 0.03 μg/mL), S-scores integrate the RF of the strain and its RF variance both in the given condition and across all conditions (*Methods*). Thus, S-scores account for RF measurement variability and facilitate the identification of strains with condition-specific growth differences. For the 307 essential/semiessential genes, sgRNAs were binned into low, medium, and high knockdown groups, with separate S-scores for each bin. Our final dataset consists of a matrix of S-scores for each of the 4,776 genes/bins by the 149 conditions that passed our quality-control criteria (Dataset S4 and *Methods*), and contains 13,104 significant (|S-score| > 5) and 5,783 strong (|S-score| > 7) chemical–gene interactions, as well as many weaker interactions (Fig. 2 and Dataset S4). Many of these recapitulated previously reported chemical–gene interactions: knockdown of *fabF* sensitized cells to cerulenin (4, 24), knockdown of *vmlR* sensitized cells to lincomycin (25), and the essentiality of *fpa* [*ylaN;* (26)] was rescued by excess iron (4, 27) (Dataset S4).

Hierarchical clustering of our S-scores revealed genes with the most similar phenotypes (Fig. 2), and identified functional modules that span multiple operons. For example, the essential folate synthesis genes *dfrA* and *folBCE* cluster together, as expected (Fig. 2, *Inset 1*). The terminal aa3 oxidase encoded by *qoxABCD*, which uses a heme A cofactor, clusters with the heme A biosynthesis genes (*ctaAB*) and *ctaM*, which is required for Qox activity [(28); Fig. 2, *Inset 2*]; demonstrating that related genes can have similar phenotypes beyond specific drug–target interactions. Finally, the peptidoglycan (PG) hydrolase *cwlO* clusters with its regulator, *ftsEX*, and with FtsEX cofactors *sweCD* [(29); Fig. 2, *Inset 3*].

More broadly, phenotypic correlations generally indicated functional connections: a majority of gene pairs with highly correlated phenotypes (*r* > 0.90, 674/1,056; Dataset S5) had functional interactions documented in the STRING database (even when accounting for the polarity of CRISPRi knockdown by excluding intraoperon interactions; *SI Appendix*, Fig. S2 and *Methods*). Taken together, these analyses suggest that our data accurately reflect known gene–chemical and gene–gene interactions. Importantly, we found at least one strong phenotype for 18% of genes with function “unknown” in SubtiWiki [(30); Dataset S4].

### High-Throughput Chemical Genomics Expands Previous Studies.

The genome-wide and unbiased nature of our data synergizes with previous genome-wide and targeted studies, allowing us to confirm and substantially expand results from previous studies in *B. subtilis* and other species, as illustrated by the three stories below.

We recently quantified genetic interactions (GIs) between cell-envelope genes in *B. subtilis* (31). These data can be analyzed together with chemical genomics data to better understand the mechanisms underlying observed GIs. For example, our GI screen implicated four genes [*yrrS* (*ragB* (32))*, ytxG* (*facZ* (33)), *ypbE,* and *yerH* (*clcR* (34))] in cell division due to their strong negative GIs with *ezrA,* a negative regulator of FtsZ-ring formation. Here, we find that knockdown strains of *yrrS/ragB, ytxG/facZ*, and *ypbE* were highly sensitized to moenomycin (S-scores < −24; Dataset S4), which inhibits the transglycosylase (TG) activity of class A PBPs. Intriguingly, other cell division gene knockdown strains also were sensitized to moenomycin, including *ftsZ*, *minJ,* and *gpsB,* raising the possibility that undercharacterized genes strongly sensitized to moenomycin (e.g., *yabM, ydbJKL,* or *yubA*) may also function in cell division.

Inhibition of fatty acid synthesis (FAS) was previously shown to rescue growth of *B. subtilis* lacking the central PG synthase *ponA* (35). Our data significantly expand this finding, demonstrating that knockdown of other elongasome genes, including essential ones like *rodA* and *mreC,* is also rescued by limiting FAS (*SI Appendix*, Fig. S3*A*). Moreover and conversely, we found that inhibiting FAS was lethal in mutants with impaired divisomes, reinforcing the important connection between membrane synthesis and cell wall surface area and raising new questions about coregulation of these processes (*SI Appendix*, Fig. S3*A*).

Finally, our data extend recent findings in gram-negative bacteria that link the stringent response and iron metabolism to gram-positive bacteria. Damage to Fe-S clusters, required for synthesis of branched- and sulfur-containing amino acids, triggers the stringent response in *Salmonella enterica*, *Enterobacter* cloaceae, and *Klebsiella pneumoniae* (36). We found that knockdown of the *suf* operon (involved in Fe-S cluster synthesis) sensitizes *B. subtilis* to mupirocin (*SI Appendix*, Fig. S3*B*), which stops cell growth by inhibiting IleS and triggering the stringent response. Moreover, we found that depleting *mntR* [which normally represses Mn^2+^ import (37)] also sensitizes *B. subtilis* to mupirocin, likely because high Mn^2+^ disrupts the function of Fe-S cluster-containing proteins [(36); *SI Appendix*, Fig. S3*B*]. Our results orthogonally confirm disruption of iron metabolism as an input to the stringent response, which is exacerbated by mupirocin-induced amino acid limitation, and extend this connection to gram-positive bacteria.

These examples demonstrate how integrating our data with targeted and high-throughput experiments from *B. subtilis* and other bacteria allows it to transcend *B. subtilis* biology, verifying and expanding regulatory themes, generating hypotheses, and revealing cross-organism similarities.

### YneF Is a Conserved, Essential Component of the SRP.

**Fig. 3.**
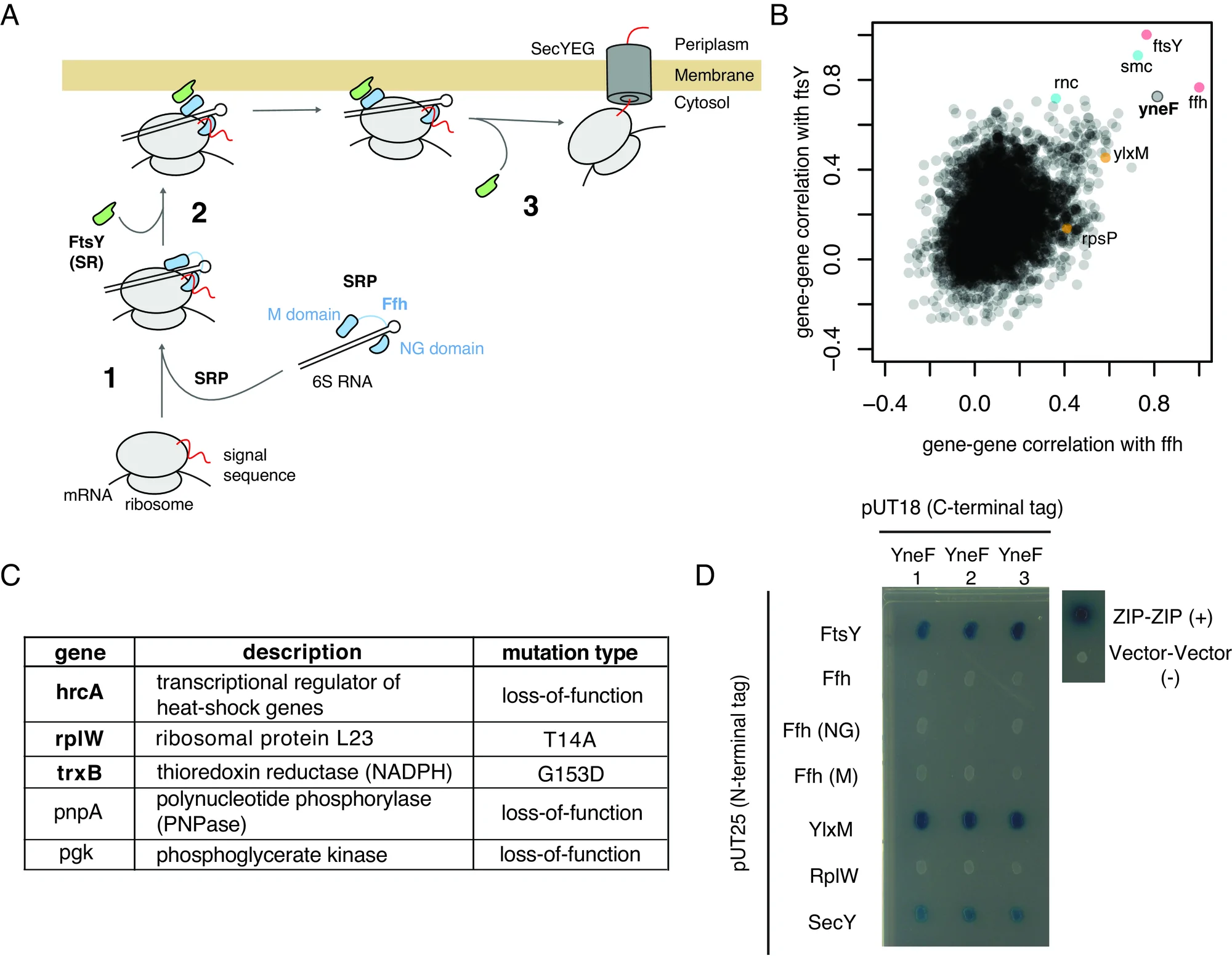
YneF is a novel, essential member of the SRP. (*A*) The SRP-mediated cotranslational protein insertion pathway. The SRP (Ffh and 6S RNA) binds to signal sequences as they are translated by the ribosome (1) and associates with FtsY (SR), which localizes the ribosome–SRP complex to the membrane (2), ultimately resulting in binding to the SecYEG translocon for protein insertion and disassembly of the complex (3). SecYEG also secretes non-SRP substrates. Schematic adapted from ref. 41. (*B*) Correlation values of each gene (n = 4162) with *ffh* are plotted against their correlation values with *ftsY* based on similarity of phenotypes across conditions. For genes with three gene knockdown bins, the maximum correlation is plotted. Circled: *yneF*. Cyan: genes sharing an operon with *ftsY* (*smc, rnc*). Orange: genes sharing an operon with *ffh* (*ylxM, rpsP, khpA*). Red: *ffh-ftsY* correlation. High correlation between *smc/rnc* and SRP genes is likely due to polar effects of their knockdown on *ftsY*. Highlighted genes with r ≈ 0 to *ffh* and *ftsY* may be occluded by other points. (*C*) Suppressor mutations that rescued growth of a *yneF* knockout mutant on LB. Bolded genes are discussed in the text. Further information is provided in Dataset S6. (*D*) Bacterial two-hybrid (BACTH) assay of YneF and other SRP components. Proteins are tagged with either the T18 or T25 domains of *B. pertussis* adenylate cyclase and three biological replicates are plated on minimal maltose media + Xgal [*SI Appendix*, *Supplemental Methods*, (42)]. Positive (ZIP) and negative (vector) controls are shown. Blue color indicates a positive interaction.

*yneF* is an essential and conserved gene across Firmicutes (5) and Tenericutes (38–40) and has been flagged as an understudied, highly expressed gene in *B. subtilis* (9). Structural predictions by AlphaFold3 suggest that YneF contains a transmembrane helix and may dimerize (assessed by interface predicted template modeling score [ipTM], the accuracy score of a predicted complex; ipTM = 0.47, *SI Appendix*, Fig. S4*A*). Our data revealed strong phenotypic correlations between *yneF,* and genes encoding proteins involved in the Signal Recognition Particle (SRP) pathway which performs cotranslational protein insertion (Fig. 3 *A* and *B*). The SRP machinery, universally conserved in all domains of life, minimally consists of 4.5S/6S/7SL RNA (in *B. subtilis*, a 6S RNA encoded by *scr*), the SRP54/Ffh protein, and the signal receptor (SR, FtsY). The SRP (6S and Ffh) binds signal sequences as they emerge from the ribosome and delivers the ribosome to SR (FtsY), triggering GTP hydrolysis and conformational changes that enable binding to the Sec (SecYEG) translocon and subsequent membrane insertion [(41), Fig. 3*A*].

Knockdown of *yneF* and other SRP components had strong positive S-scores in minimal media (MM) (Dataset S4), suggesting that this condition may rescue a *yneF* deletion. To test this, we transformed a previously designed *yneF* deletion construct (5) into WT *B. subtilis*, and recovered transformants on MM but not on LB (*SI Appendix*, Fig. S4*B*). This allowed us to identify suppressors of *ΔyneF* able to grow on LB. We streaked individual *ΔyneF* colonies growing on a MM plate onto an LB plate, selected colonies that grew, and performed whole genome sequencing. We identified 5 unique suppressors, three of which were consistent with YneF functioning in the SRP machinery (Fig. 3*C* and Dataset S6).

(a) Deletion of *hrcA* suppressed *yneF* essentiality. HrcA represses two heat shock operons (*hrcA-grpE-dnaK-dnaJ-yqeT-yqeU-yqeV* and *groES-groEL*) and its deletion results in overexpression of these genes (43). Overexpression of *dnaK/J* is among the first cellular responses to SRP depletion (44) and suppresses translocon defects in *Escherichia coli* (45), likely by maintaining the folding state of substrates until they can enter the translocon. To test whether *ΔhrcA* suppresses *yneF* via overexpression of *dnaK/J,* we constructed double deletion mutants of *hrcA* and most genes in the *hrcA* operon (e.g., *ΔhrcA*/*ΔdnaK*) and tested whether these strains could be transformed with *ΔyneF*. We reasoned that if overexpression of an operon member was required for suppressing *yneF*, the triple deletion (e.g., *ΔhrcA*/*ΔdnaK/ΔyneF*) would not be viable. We were able to construct triple deletions with every gene in the *hrcA* operon except *ΔdnaJ* and *ΔdnaK*, indicating that deletion of *hrcA* rescues *ΔyneF* by overexpression of *dnaJ* and *dnaK* (*SI Appendix*, Fig. S4*C*).
(b) A point mutation (G153D) in *trxB,* which encodes thioredoxin reductase, suppressed *yneF* essentiality. We speculate that this mutation may enhance the TrxB thiol-dependent antioxidant system, protecting misfolded proteins from thiol oxidation.
(c) A point mutation (T14A) in *rplW,* which encodes the essential L23 ribosomal protein, suppressed *yneF* essentiality. L23 plays a crucial role in the interaction between the ribosome and the SRP (46, 47) and the translocon (48–50), providing another link between YneF and the SRP pathway. Together, these suppressors support a role for YneF in the SRP pathway.

To better understand the function of YneF in the SRP machinery, we tested whether YneF associates with SRP components using an adenylate cyclase-based bacterial two-hybrid system in *E. coli* [BACTH; (51); Fig. 3*D* and *SI Appendix*, Fig. S4*D*]. We constructed N- and C-terminally tagged versions of YneF, FtsY, Ffh (the entire protein and NG- and M domains alone), RplW, SecY, and YlxM [a Firmicute-specific SRP accessory protein encoded upstream of and cotranscribed with *ffh* (52)], and tested the interactions of C- and N- terminally tagged YneF with all other proteins. C-terminally tagged YneF interacted with several proteins (Fig. 3*D*) whereas N-terminally tagged YneF did not exhibit any interactions (*SI Appendix*, Fig. S4*D*), likely because the N-terminal adenylate cyclase fragment precludes proper membrane insertion. The interaction between YneF and the transmembrane components of the SRP (SecY and FtsY) is consistent with its predicted localization and establishes its involvement in the SRP. YneF also interacted with YlxM. YlxM modulates Ffh GTPase activity (52), raising the possibility that the interaction of these Firmicute-specific SRP components synchronize the cytoplasmic and membrane components of the SRP, perhaps working with the additional Alu domain of *B. subtilis* to effect developmental programs such as sporulation (53). Taken together, our phenotypic data, suppressor screen, and BACTH data firmly establish a role for YneF in the SPR machinery.

### YbfF Drives Resistance to β-Lactams.

**Fig. 4.**
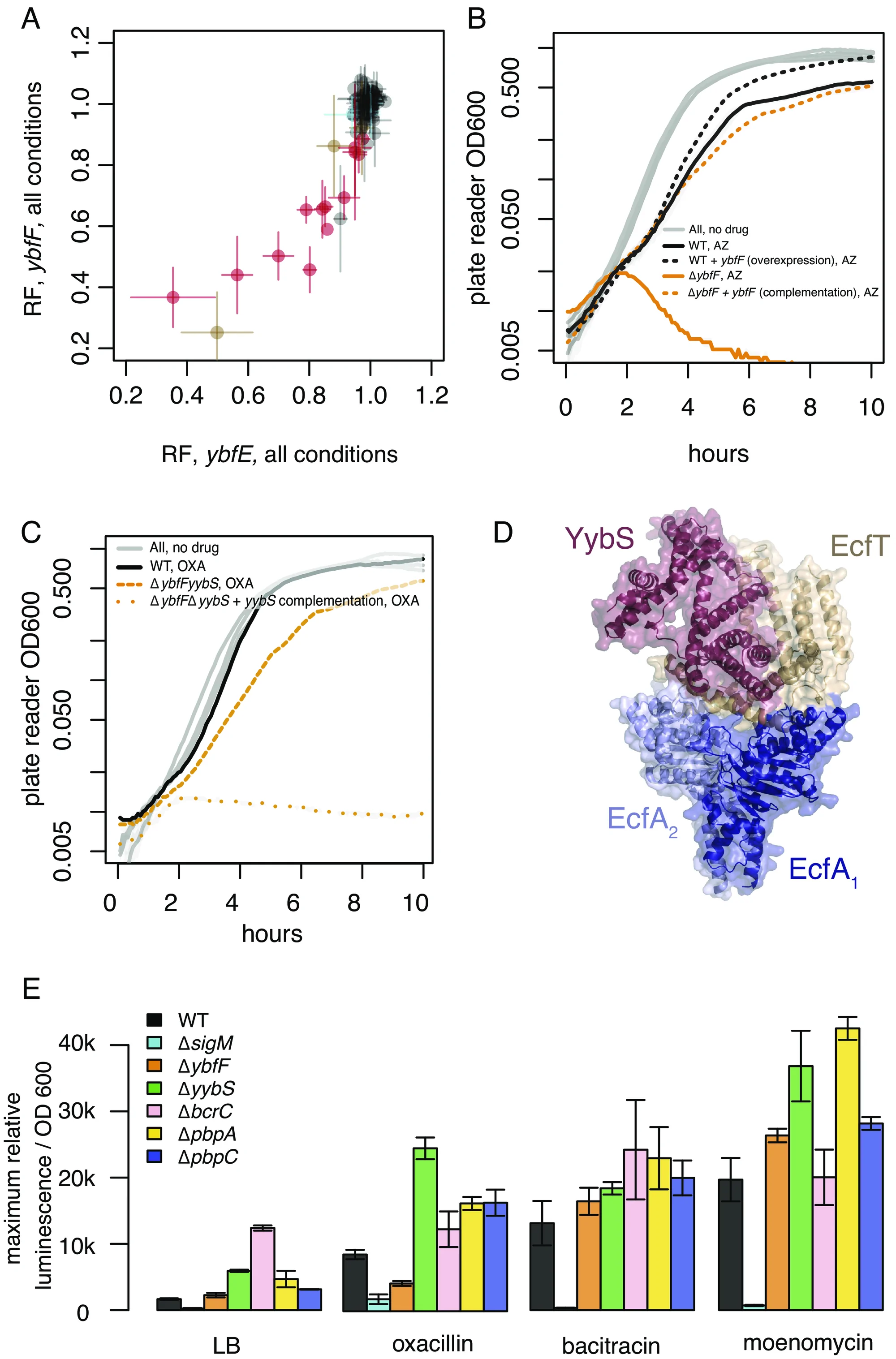
YbfF and YybS are contributors to cell wall homeostasis. (*A*) Average RF values of *ybfE* and *ybfF* knockdown strains over all conditions (all concentrations of each stressor). Red = β-lactams; brown = EGCG, a catechin suggested to directly bind PG and cell surface proteins (54, 55); cyan = other cell wall antibiotics (moenomycin, bacitracin, cycloserine D, and fosfomycin; all with RFs ≈ 1.0). (*B*) YbfF protects against β-lactams dependent on its expression. WT (WT + P_xyl_-ybfF at *lac* without inducer), *ybfF* overexpression (WT + P_xyl_-ybfF at *lac* + 1% xylose induction), *ΔybfF* (*ΔybfF* + P_xyl_-ybfF without inducer), and *ybfF* complementation (*ΔybfF* + P_xyl_-ybfF + 1% xylose induction) were grown from a 1:100 dilution in LB only (gray, all strains) or LB + 6 μg/mL aztreonam (AZ). (*C*) Deletion of *yybS* rescues β-lactam sensitivity of *ΔybfF* deletion. This effect is reversed when *yybS* is exogenously expressed, indicating that this is due to the presence of YybS and not to a polar effect. Mid-log cultures of WT, *ΔybfFΔyybS* (*ΔybfFΔyybS* + P_xyl_-*yybS* without inducer), and *ΔybfFΔyybS* + *yybS* complementation (*ΔybfFΔyybS* + P_xyl_-*yybS* + 1% xylose induction), were diluted 1:100 in LB only (gray, all strains) or LB + 0.04 μg/mL oxacillin (OXA). (*D*) Predicted AlphaFold3 structure of YybS, EcfA_1_, EcfA_2_, and EcfT (complex ipTM = 0.84). YybS has predicted interactions with EcfT (ipTM = 0.81), EcfA_1_ (ipTM = 0.78), and EcfA_2_ (ipTM = 0.76). (*E*) Maximum relative luminescence per OD600 during 4 h of growth of WT, *ΔsigM*, *ΔybfF, ΔyybS, ΔbcrC, ΔpbpA*, and *ΔpbpC* strains with a P_lux_-SigM reporter (*SI Appendix*, *Supplemental Methods*). Strains were diluted into LB + 0.15 μg/mL oxacillin, LB, LB + 40 μg/mL bacitracin, or LB + 0.4 μg/mL moenomycin in a 96-well plate and OD600 and luminescence were measured. *ΔybfF* displays a depressed SigM response in response to the β-lactam oxacillin, but not the non-β-lactam antibiotics moenomycin or bacitracin. *ΔyybS* displays a heightened SigM response compared to WT in all growth conditions, including LB. Full growth and luminescence curves provided in *SI Appendix*, Fig. S7.

The most successful and widespread class of antibiotics are those that target the cell wall, particularly β-lactams, which inhibit the transpeptidation (TP) of PG. To identify genes underpinning resistance to β-lactams, we searched for strains with β-lactam-specific phenotypes. Knockdowns of *ybfE* and *ybfF*, in the putative *ybfGFE* operon, were consistently, specifically, and exquisitely sensitized to β-lactams, but not to other PG-targeting drugs such as the TG inhibitor moenomycin or the undecaprenyl pyrophosphatase inhibitor bacitracin (Fig. 4*A*). Testing the phenotypes of single gene deletions (5) to deconvolute CRISPRi operon effects revealed that only *ΔybfF* was sensitized to β-lactams (*SI Appendix*, Fig. S5*A*). *ybfF* encodes a unique polytopic membrane protein predicted to dimerize by AlphaFold3 (ipTM = 0.71) (*SI Appendix*, Fig. S5*B*) and restricted to *B. subtilis* and closely related species. The *ΔybfF* mutant lysed in the presence of β-lactams, and this phenotype was rescued by complementation with *ybfF* (Fig. 4*B*). Importantly, overexpression of *ybfF* in a WT background resulted in increased resistance to β-lactams compared to a WT control (Fig. 4*B*), indicating that YbfF is a bona-fide β-lactam resistance mechanism.

### YybS Is an ECF-Transporter Specificity Subunit Implicated in Cell Wall Homeostasis.

We identified six *ΔybfF* colonies that grew within the zone of inhibition of aztreonam and subjected them to whole genome sequencing to identify suppressors. We found loss-of-function mutations in *ecfA_2_* and *ecfT,* which encode components of the energy coupling factor (ECF) transporter, and in *yybS,* a polytopic DUF2232-family protein (Dataset S6). Because *yybS* is encoded upstream of *gdpP*, a c-di-AMP phosphodiesterase with β-lactam phenotypes (56, 57), we constructed a clean deletion of *yybS* and excised the antibiotic resistance cassette (5) to ensure that *gdpP* expression was not artificially upregulated by the downstream promoter driving the KanR cassette. We used this strain to validate that *yybS* was solely responsible for suppressing *ΔybfF* sensitivity to aztreonam (and for all subsequent *yybS* experiments). Complementing the *ΔyybS* strain restored β-lactam sensitivity (Fig. 4*C*), confirming that the observed phenotype is due to *yybS* and not other genes in the operon.

Given that *ecfA_2_* and *ecfT* were recovered as suppressors of *ΔybfF* aztreonam sensitivity in addition to *yybS*, we wondered whether *yybS* function was associated with ECF transporters. The core ECF transporter is composed of *ecfA_1_, ecfA_2_*, and *ecfT* and reversibly binds to substrate-specific (S) subunits to import various nutrients, metals, and vitamins (58, 59). Several analyses suggest that YybS functions as a ECF transporter S-subunit. First, YybS exhibited significant structural similarity to other ECF S-subunits (FoldSeek (60) median e-value: 1.32e−2, *SI Appendix*, Fig. S5*C* and Dataset S7). Second, AlphaFold3 predicted a high-confidence complex between YybS and the ECF transporter complex (Fig. 4*D*; overall ipTM = 0.84), and in this predicted complex YybS bound in the same location and orientation as an S-subunit (e.g., PanU; RMSD = 0.607 Å). Finally, genomic databases indicate that some Actinobacterial DUF2232 domains are fused to transporter nucleotide binding domains like those of EcfA_1_/A_2_ or to EcfT homologs [e.g., A0A2U1E8B4 from *Actinomycetospora cinnamomea*, (61)]. Taken together with the suppressor data, these structural analyses suggest that YybS associates with the core ECF transporter to affect the β-lactam sensitivity of the *ΔybfF* mutant.

### GIs, PG Structure, and SigM Induction of *ΔyybS* and *ΔybfF* Mutants.

Extensive studies of PG homeostasis have identified penicillin-binding proteins (PBPs) and SigM as key players whose depletion results in altered sensitivity to cell wall targeting drugs. We therefore performed a series of GI experiments between *ponA*, *pbpA*, *pbpC*, and *sigM* and both *ybfF* and *yybS*. First, since we initially identified *ΔyybS* as a suppressor of *ΔybfF,* we tested whether *ΔyybS* could also suppress the β-lactam sensitivity of *ΔponA*, *ΔpbpA, ΔpbpC,* and *ΔsigM* mutants. *ΔyybS* partially rescued the β-lactam sensitivity of all four mutants as well as the growth defect of *ΔponA*, suggesting that *ΔyybS* is a general suppressor of β-lactam sensitivity and PG homeostasis (Fig. 4*C* and *SI Appendix*, Fig. S5 *D*–*H*). Consistent with this hypothesis, EcfA_1_ was identified in a separate screen as a suppressor of *ΔponA* β-lactam sensitivity (Dataset S6). Second, we tested whether the original YbfF mutant functions by activating PonA, PbpA, PbpC, or SigM. We reasoned that if YbfF is specifically required for the activity of one of these proteins, the double deletion strain would phenocopy that of the single PBP deletion strain. Assessing the β-lactam sensitivity of *ΔybfF/ΔponA*, *ΔybfF/ΔpbpA, ΔybfF/ΔpbpC,* and *ΔybfF/ΔsigM* strains revealed that all double deletion strains were more sensitive to β-lactams than the individual mutants, indicating that YbfF does not function solely by activating one of these proteins (*SI Appendix*, Fig. S5 *H*–*K*).

Given the effects of *ΔybfF* and *ΔyybS* on cell wall homeostasis, we also tested whether they exhibited altered PG structure during exponential growth in LB using HPLC [(62), *SI Appendix*, *Supplemental Methods*]. Both *ΔybfF* and *ΔyybS* cells had a PG composition similar to WT (*SI Appendix*, Fig. S6), indicating that neither grossly affects PG composition in the absence of stress.

We next assessed SigM induction in the *ΔyybS* and *ΔybfF* mutants using a previously described SigM reporter. Decades of research have established that SigM upregulates a large regulon in response to reduced levels of undecaprenyl phosphate (UndP) caused by diverse cell wall stressors (56, 63, 64). Consistent with this role, SigM a was the primary sigma factor required to resist β-lactams in our screen (by S-score, compared to other sigma factors implicated in cell wall homeostasis, Dataset S4). Low UndP levels can be caused rapidly and directly by a drug that sequesters UndP (e.g., bacitracin) or slowly and indirectly by drugs that inhibit PG synthesis (e.g., β-lactams), which then cause UndP sequestration in UndP-WTA that cannot be ligated due to blocked PG synthesis (65).

SigM induction was reduced in the *ΔybfF* strain in response to the β-lactam oxacillin (*ΔybfF* maximum signal ~0.48x that of WT; Fig. 4*E* and *SI Appendix*, Fig. S7), but not in response to moenomycin or to bacitracin (*ΔybfF* maximum signal ~1.3x that of WT in both conditions; Fig. 4*E* and *SI Appendix*, Fig. S7), implying that the *ΔybfF* strain has more available UndP than WT in the presence of oxacillin (but not moenomycin or bacitracin). This could result from either reduced WTA synthesis or increased PG synthesis in *ΔybfF* relative to WT in the presence of oxacillin. Since reduced WTA synthesis does not affect β-lactam sensitivity in our screen (Dataset S4), the most parsimonious explanation is that under β-lactam stress, the *ΔybfF* strain has increased PG synthesis compared to WT. One possibility is that YbfF synchronizes TP and TG activity by repressing TG activity in the absence of TP activity. If so, in the absence of *ybfF,* TG activity would continue even when TP activity is reduced by β-lactams, producing PG that allows WTA attachment but lacks crosslinks. This would explain the β-lactam-specific (and moenomycin independent) phenotypes of the *ΔybfF* strain: its exquisite sensitivity and its lack of SigM activation. Future work measuring PBP (e.g., ref. 66) and autolysin (e.g., ref. 67) activity under β-lactam stress in these strains will further elucidate the role of YbfF.

In the *ΔyybS* strain, SigM was overinduced in response to every tested cell wall drug (*ΔyybS* maximum signal ~1.4-2.9x that of WT; Fig. 4*E* and *SI Appendix*, Fig. S7); even basal SigM expression was higher than WT (*ΔyybS* maximum signal ~3.6x that of WT; Fig. 4*E* and *SI Appendix*, Fig. S7), suggesting a slight but pervasive defect in UndP availability, perhaps due to a requirement for the substrate of YybS in UndP synthesis, transport, or regulation. We hypothesize that slightly decreased UndP availability may limit futile cycling (68), explaining how *ΔyybS* suppresses diverse β-lactam sensitivity phenotypes.

Taken together, these data identify YybS and YbfF as major players in cell wall homeostasis in *B. subtilis.* Intriguingly, despite its importance, YbfF homologs are only found in *B. subtilis* and closely related species, suggesting that YbfF may be a recent or *Bacillus*-specific innovation. By contrast, the importance of YybS and the ECF transporter is not limited to *Bacilli*; *S. aureus* transposon insertion mutants of *yybS* and *ecfT* exhibited highly similar phenotypes, including resistance to lysobactin, an antibiotic that binds to Lipid II (*SI Appendix*, Fig. S8 *A* and *B*), and YybS-like (DUF2232) proteins are widespread in firmicutes and present in other phyla. YybS-like proteins are essential in *Azospirillum brasilense* (an α-proteobacteria) (69), *Synechococcus elongatus* (a cyanobacteria) (69), and *C. difficile* (a firmicute) (70), highlighting the importance of this ECF S-subunit.

### Adaptation to Environmental Change by Community Members Driven by Diverse Response Mechanisms.

*B. subtilis* has evolved in the presence of numerous bacterial and fungal competitors (71) and is known to induce stress responses in the presence of antimicrobial compounds produced by other bacilli (e.g., ref. 72). Conditioned media experiments, where one species is grown in the supernatant of another species, have been used to model interspecies interactions such as inhibition (73), cross-feeding (74, 75), or resource competition (76, 77). To understand the genetic underpinnings of *B. subtilis* competition with other species, we grew our library in the presence of conditioned media from a wide range of ecologically relevant organisms [(71), *SI Appendix*, *Supplemental Methods* and Dataset S8]. We identified three major sets of genes required for growth in the presence of spent media from multiple bacterial species.

**Fig. 5.**
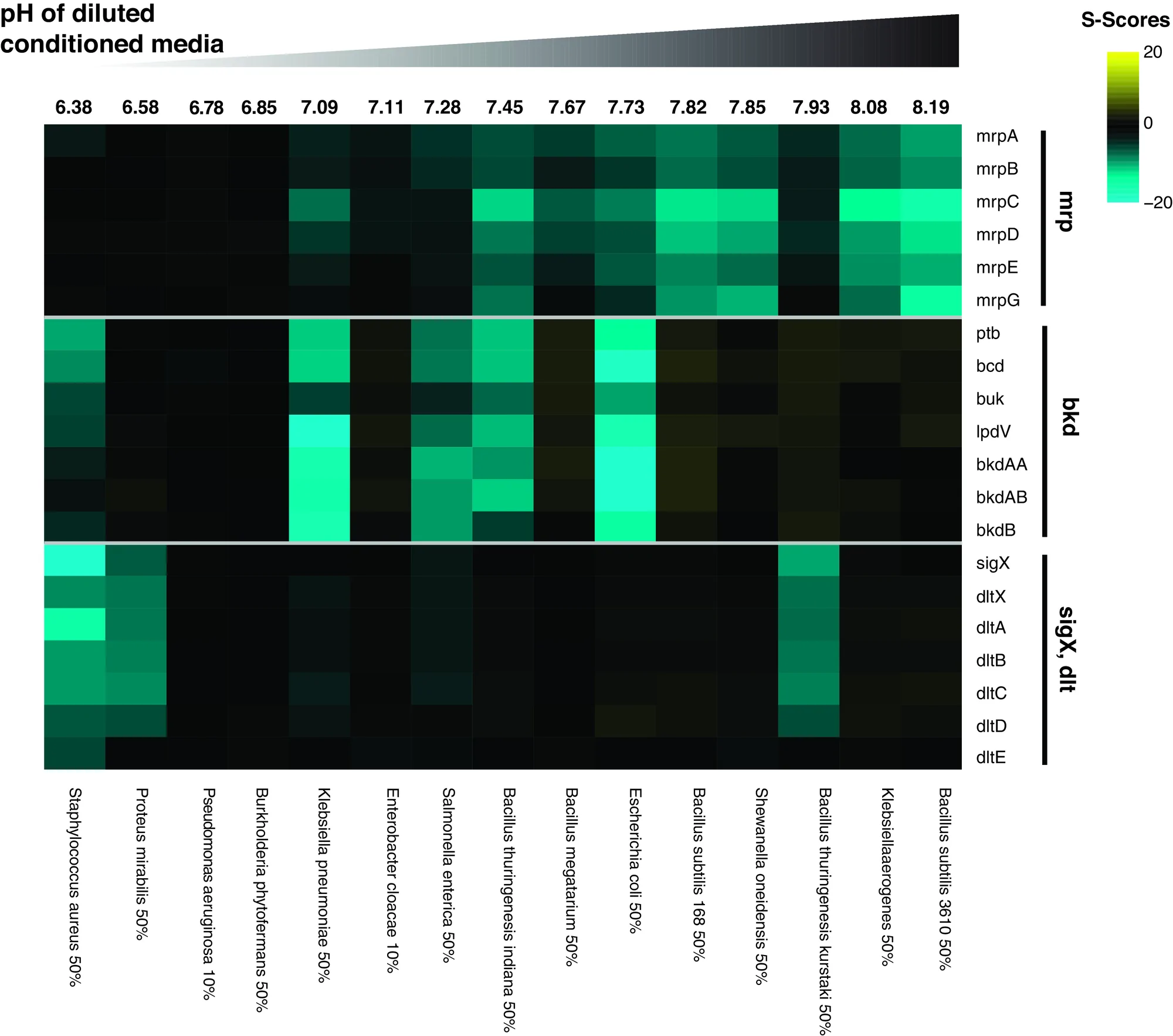
Major processes required for growth in diverse bacterial conditioned media. Heatmap of gene–chemical S-scores of operons required for growth in multiple bacterial conditioned media. Conditioned medium from each strain (x-axis, *Bottom*) is sorted by the pH of the conditioned media after dilution with fresh LB and before growth of the library (dilution percentage noted after the strain name, *SI Appendix*, *Supplemental Methods*). LB used in this screen is pH = 7.0. For genes targeted by multiple knockdown bins, only the most responsive bin (greatest sum of absolute S-scores across bacterial conditioned media) is shown.

First, knockdown of genes in the *mrp* operon, which encodes the essential Na+/H+ antiporter complex, strongly sensitized *B. subtilis* to conditioned media from *Klebsiella* aerogenes, Shewanella oneidensis, and *B. subtilis* strains (Fig. 5). Mrp antiporters (for “Multiple resistance and pH”) play a role in resisting diverse stresses, particularly alkaline stress (78, 79), raising the possibility that those strains change the pH of the media. Consistent with this hypothesis, we found that the growth of *mrp* knockdown strains was strongly negatively correlated with the pH of the conditioned media used in the screen (r = −0.85 between pH and S-scores of medium-knockdown *mrpA* bin; *SI Appendix*, *Supplemental Methods*): *mrp* knockdown strains grew more poorly in conditioned media that is more alkaline. pH is a major driver of microbial distribution and success in the soil (80) and the increased sensitivity of *mrp* knockdown mutants to alkaline conditioned media emphasizes the importance of maintaining pH homeostasis in the presence of other bacteria.

Second, knockdown of the *bkd* operon (*ptb*-*bcd*-*buk*-*lpdV*-*bkdAA*-*bkdAB*-*bkdB,* hereafter *bkd*) sensitized cells to conditioned media from *E. coli*, *B. thuringiensis* var. Indiana, *Klebsiella pneumoniae*, and *S. aureus* (Fig. 5). *bkd* encodes the branched-chain keto acid dehydrogenase complex, which converts branched-chain amino acids to branched-chain fatty acids (BCFAs) (81), affecting membrane fluidity (82, 83). In *B. subtilis, bkd* is important for cold adaptation (84). *bkd* knockdown strains additionally exhibited impaired growth in MM, glucose complete medium, and at pH 7.5 in our screen (Dataset S4), suggesting multiple ways in which *bkd* knockdowns could be sensitized to conditioned media. Our data implicate the production of BCFAs and, by extension, the alteration of membrane fluidity as an important aspect of interspecies competition.

Finally, knockdown strains of *sigX* (an ECF-type sigma factor) and the *dlt* operon [*dltXABCDE*, part of the SigX regulon (85)] were sensitized to conditioned media from *Proteus mirabilis*, *S. aureus*, and *B. thuringiensis* var. *Kurstaki* (Btk) (Fig. 5). SigX and Dlt are required for resistance to cationic antimicrobial peptides (CAMPs); SigX is activated by CAMPs and transcribes the *dlt* operon (85). Dlt lowers the net negative charge of the cell envelope by adding an alanine to the negatively charged teichoic acids, rendering the cell less susceptible to CAMPs (85). CAMPs, such as bacteriocins, are widely produced by bacteria, including *S. aureus* and Btk (86–88). That *sigX* and *dlt* knockdown strains are sensitized to the conditioned media of specific species suggests that those species secrete relatively broad-spectrum CAMPs even when growing as a monoculture. Activation of the *dlt* operon may also be required to survive additional coculture stressors beyond antimicrobials.

Together, these data demonstrate our screen captures responses to complex stress conditions and reveal major factors for resisting the environmental changes produced by the growth of other bacterial community members.

### Glutamate and Alanine Are Crossfed in Gram-Positive But Not Gram-Negative Bacteria.

**Fig. 6.**
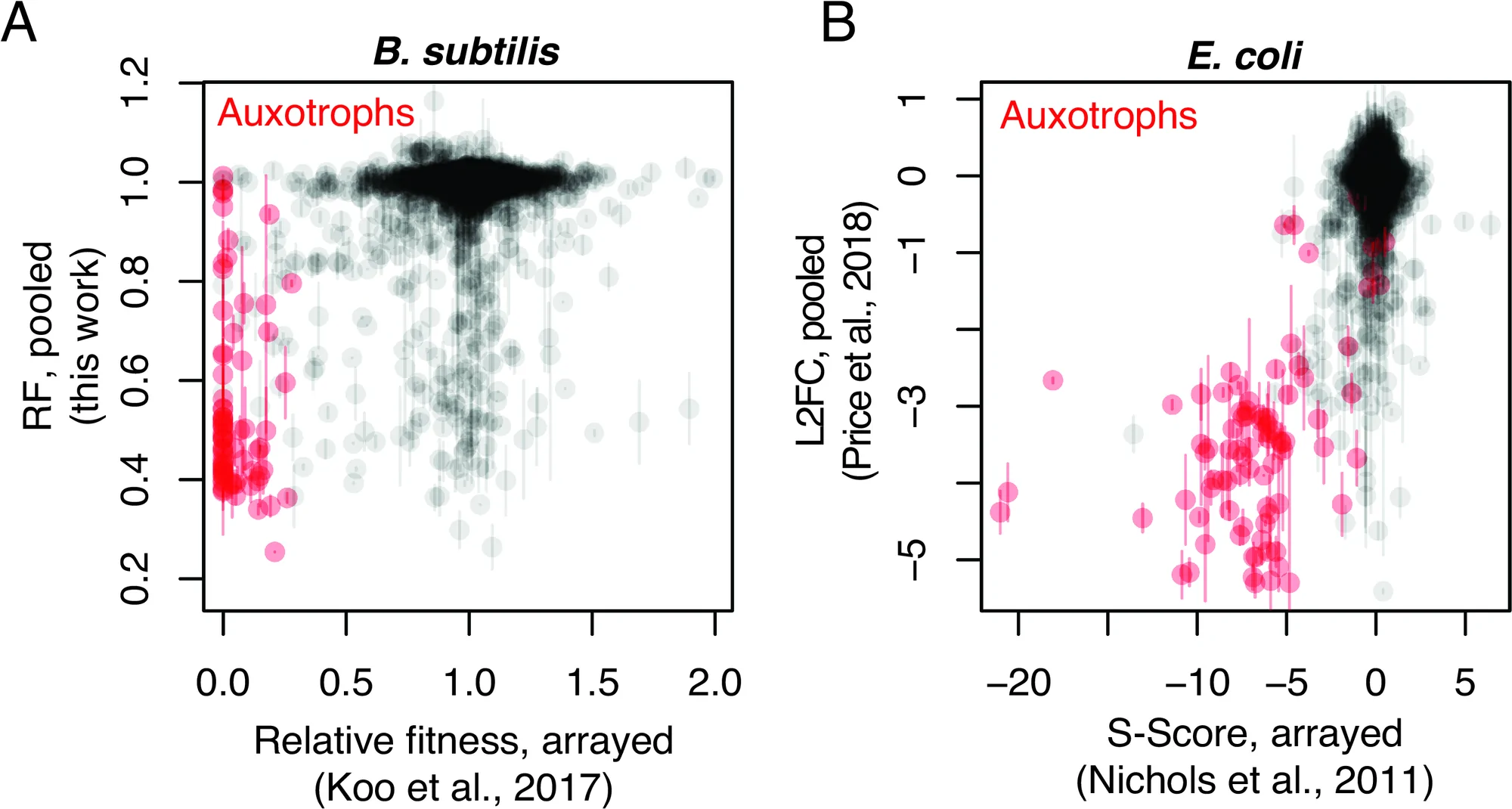
Cross-feeding in *B. subtilis* and *E. coli*. (*A*) Growth of *B. subtilis* mutant strains grown either as single colonies of knockouts on MM agar plates [x-axis, (5)] or as a pooled knockdown library in MM (y-axis) (all genes present in both libraries; n = 3774 genes). WT growth has an RF = 1.0 for both the single colony and liquid culture experiments. Error bars signify SD of RF values in MM of the two targeting sgRNAs in pooled culture. Auxotrophs are highlighted in red (defined in ref. 5). Cross-feeding behavior of a strain manifests as a RF of 0 as single colonies and a RF of 1.0 in pooled culture (*Upper Left* quadrant). (*B*) Growth of *E. coli* knockout strains (91) grown as single colonies on MM agar plates [x-axis, (10, 92)] or in pooled liquid MM culture growth [y-axis, (69)] (all genes present in both screens; n = 3,410 genes). For single colonies, WT growth is signified as an S-score of 0. For growth in liquid culture, WT growth is signified as a log_2_ fold change (L2FC: doublings of mutant strain minus growth doublings of the bulk library culture) of 0. Error bars signify SD of two separate measurements of growth in pooled culture. Auxotrophs are highlighted in red (defined in ref. 5). Cross-feeding behavior of a strain would manifest as a negative S-score (i.e., S-score < −5) as single colonies and a L2FC of 0 in pooled culture (*Upper Left* quadrant).

Cross-feeding enables the metabolic products of one bacterial species to sustain another one. These interactions are important for determining community composition and structure in the soil and the gut (75, 89, 90), but are incompletely characterized. We performed a global unbiased analysis of cross-feeding in *B. subtilis* by comparing the growth of our pooled knockdown library in MM (which enables crossfeeding) to the growth of an arrayed knockout library on plates of the same MM (5) (where colonies are too far apart to permit crossfeeding). We identified 5 knockdown strains (*gltA, gltB, gltC, icd,* and *alaT*) that appear to be cross-fed in the pooled condition but not in the arrayed condition (Fig. 6*A*).

To test whether this phenomenon represented true cross-feeding or was a byproduct of differences in growth between liquid and solid media (or between knockouts and knockdowns), we tested the ability of the *ΔgltA, ΔgltB,* and *ΔalaT* strains to grow on solid MM away from or in close proximity to WT *B. subtilis* using a previously developed cross-feeding assay (93). All three auxotrophic strains were able to grow on MM near (but not away from) WT. In contrast, non-cross-fed *ΔhisA* and *ΔcysJ* strains, which grew poorly on MM in both the arrayed- and pooled screen, were unable to grow on MM regardless of the presence of WT (*SI Appendix*, Fig. S9), confirming the results of our analysis.

The *E. coli* genome-wide knockout library (91) has also been screened in MM in both pooled (69) and arrayed (10, 92) settings. We therefore applied the same analysis to assess cross-feeding behavior in *E. coli*, a gram-negative bacterium. Remarkably, we found no evidence of cross-feeding in *E. coli*: No mutants sick as single colonies showed growth akin to WT in pooled media (Fig. 6*B*), suggesting that *E. coli* is unable to support growth of any auxotrophs via cross-feeding. Because L-alanine, D-alanine, and D-glutamate are part of the *B. subtilis* and *E. coli* PG stem peptides (94), crossfeeding differences between gram-positive and gram-negative bacteria may be due to differences in PG recycling. Gram-positive bacteria perform multiple steps of PG recycling extracellularly, including the separation of the stem peptide (95–98), which may diffuse away and cross-feed auxotrophs or other organisms. In contrast, gram-negative bacteria retain over 90% of PG peptides within the periplasm for reuse (96, 99). More generally, gram-positive PG turnover and recycling may be an overlooked component of community-level nutrient metabolism.

## Perspective

As a model Firmicute, *B. subtilis* anchors our understanding of this diverse phylum, whose members play important roles in health, disease, industry, and ecosystems. The importance of *B. subtilis* is underscored by decades of research and by the arsenal of tools developed to probe its biology; for example, it is the only gram-positive organism with a genome-wide single gene deletion library (5). However, despite extensive studies, many core aspects of firmicute biology remain hidden, as illustrated by several recent foundational discoveries: runaway transcription (100), eukaryotic-like ribosome quality control (101), and DedA UndP flippases (102, 103). To reveal new facets of firmicute biology and facilitate hypothesis generation, we performed a full-genome, CRISPRi-based chemical genomics screen in *B. subtilis*. Our screen tested ~150 diverse conditions (increasing the interpretability of correlated phenotypes), used CRISPRi instead of Tn-seq or knockout libraries (allowing us to query essential gene phenotypes), and optimized library design (facilitating handling and multiplexing). The data from our screen identified and filled many gaps in our understanding of *B. subtilis* biology and will serve as a resource for the field.

First, we identified roles and phenotypes for genes of unknown function, including essential genes, setting the stage for future mechanistic studies. For example, our screen guided experiments which demonstrated that *yneF*, a highly expressed, essential, and deeply conserved gene with no annotated function, encodes an auxiliary component of the SRP pathway and physically interacts with the universally conserved pathway components (FtsY and SecY), and with the Firmicute-specific component YlxM, raising the possibility that these two auxiliary members contribute to the regulation of pathway performance. Moreover, we implicated and characterized two uncharacterized genes (*ybfF* and *yybS*) in cell wall homeostasis, showing that the narrowly conserved protein YbfF plays a major and specific role in resisting β-lactams, and that the more broadly conserved DUF2232-family protein YybS is an ECF transporter S-subunit that may affect UndP synthesis. Beyond these findings, our dataset contains many unexplored gene–chemical and gene–gene interactions, which will nucleate future studies targeting core aspects of firmicute biology.

Second, our dataset contains information on the genetic determinants of susceptibility and resistance for numerous antibiotics and antimicrobial compounds covering a spectrum of targets and structural classes. These data can be leveraged to determine the mechanism of action (MoA) of novel antibiotics, an approach that has been demonstrated in *S. aureus* and in *B. subtilis* (4, 11). Importantly, because our CRISPRi screen includes essential genes and ~150 chemical conditions, it can be used to accurately predict MoAs for diverse antimicrobials. Additionally, our data can be mined to provide insight into overlapping MoAs of different compounds, revealing and informing synergistic antibiotic combinations (104).

Finally and most importantly, our phenotypic data are a critical part of an integrated global approach for understanding *B. subtilis* and other firmicutes, providing important functional information for interpreting existing datasets, such as proteomic (e.g., refs. 6, 105, and 106) and transcriptomic (e.g., refs. 2, 107, and 108) associations between genes. The firm experimental footing provided by chemical genomics experiments will become even more important for validating and interpreting results from completely computational (e.g., AI-based) approaches. For example, we recently developed pooled-AlphaFold3, a strategy that enables accurate genome-scale screening of protein–protein interactions (PPIs) (109), and demonstrated the power of combining such an analysis (110) with chemical genomics (20) to decipher gene function and pathway connections in Mycobacteria. As genome-scale PPI screening becomes increasingly widespread, we envision compact genome-wide CRISPRi chemical genomics screens as necessary complements for revealing bacterial gene functions and connections.

In summary, our broad and comprehensive screen has revealed novel aspects of *B. subtilis* biology with implications for other gram-positive bacteria. These data will serve as a valuable resource for the *B. subtilis* research community, acting as a “hypothesis-generator” for future studies and integrating with future and existing genome-wide datasets. Our approach, with its compact and information rich sgRNA library design, is a model for future chemical genomics screens in diverse bacteria, which will bridge the gene–function gap and expand our knowledge of bacterial biology.

## Methods

### General Strain Manipulations and Procedures.

Unless otherwise noted, all strain construction and growth assays for *B. subtilis* were done in LB medium using antibiotics at the specified concentrations: Spectinomycin (100 μg/mL), Chloramphenicol (6 μg/mL), Kanamycin (7.5 μg/mL). LB, glucose complete, and minimal media were prepared as previously described [Dataset S1, (5)].

### sgRNA Plasmid Construction.

pJSHa77 (16) was modified to include a 6 bp unique molecular identifier (UMI). pJSHa77 was digested with BamHI and an NNNNNN sequence was inserted into the BamHI digestion site with NEB HiFi (NEB #C2987) to create pJSHa77b.

### CRISPRi Library Construction.

A *B. subtilis* CRISPRi library was designed and cloned as previously described (16, 111), with minor modifications. Oligos encoding sgRNAs included ~100 bp homology with the pJSHa77b backbone were ordered from Twist Biosciences and amplified for 14 cycles. The pJSHa77b backbone was digested with BsaI-HFv2. The oligo library was inserted into the backbone via NEB HiFi and transformed into NEB 10beta *E. coli*, recovered, then grown overnight in 100 mL LB and midiprepped the following day with the Qiagen Plasmid Plus kit (Qiagen #12945). The plasmid library was digested with NdeI for 3 h at 37 °C, cleaned up, and transformed into *B. subtilis* as previously described (16).

### sgRNA Library Design.

The CRISPRi library targets the full genome: Each essential gene was targeted by 12 sgRNAs with a range of predicted knockdown efficacies, and each nonessential gene was targeted by 2 fully complementary sgRNAs for a total library size of 11,559 sgRNAs. Selection of specific sgRNA categories are expanded below.

#### Essential genes.

The essential gene set was defined as the set of 300 genes previously investigated in ref. 16. Using experimental RF data from ref. 16, two of the 10 parent sgRNA families from each gene were selected from the original library. Parent sgRNA families were selected based on distance from the median experimental RF line. 5 of the 10 daughter mismatch sgRNAs of varying knockdown efficiencies were selected from each family based on bins and distance from the median as described in ref. 16.

#### Nonessential genes with Koo 2017 phenotypes (“Impaired” genes).

17 nonessential gene knockouts in ref. 5 had significant growth phenotypes in LB at 37 °C. Of these, 10 were already included in ref. 16 and the library was selected as described in “Essential Genes”. The remaining 7 (*mbl, lipL, qoxABCD,* and *rpmH*) were targeted by the two parent sgRNAs with the highest score and 10 mismatch daughters as described in refs. 16 and 17.

#### Nonessential genes.

For nonessential genes without strong phenotypes in ref. 5 and passing exclusion criteria, sgRNAs were generated as described in ref. 17 and scored based on observed knockdown efficacy weights (sequence logo in ref. 17). sgRNAs with more than 10 off-target hits and sgRNAs containing BsaI or NdeI restriction sites (used in cloning steps) were excluded. For each gene, the two highest-scoring sgRNAs were selected.

### Chemical Genomics Experiments and Sequencing.

Library aliquots were thawed, diluted to OD600 0.01, and grown in appropriate media (LB for all chemical stressors; MM, GC, or TSB if an alternative medium was being tested) at 37 °C to an OD600 of 0.10. From here, a t_0_ sample was taken, and the culture was back diluted to OD600 0.01 in 37 °C growth media with the stressor and 1% xylose to induce dCas9 expression. When possible, two concentrations of each stressor were chosen for a 10% or 25% reduction in growth rate relative to WT *B. subtilis* in LB. After approximately 5 doublings, the culture was back diluted 1:10 into fresh media + 1% xylose and chemical stressor; after another 5 doublings, the culture was pelleted and frozen. OD600 was measured at each back-dilution and before collection via Synergy H4 plate reader (BioTek) calibrated to OD600 spectrophotometer readings. Genomic DNA was extracted from samples with the DNeasy Blood and Tissue Kit (Qiagen #69581) and libraries were prepared, pooled, and sequenced using Illumina NovaSeq as previously described (16).

### Relative Fitness Analysis and Correction.

RF values were calculated from sequence counts as previously described (16, 111), with minor adjustments. Briefly, for each strain *X* with at least 10 counts at t_0_, we calculate the RF *F*(*x*) as

$$F\left(x\right)=\frac{\log\;2\;\frac{r_{\mathrm{wt}}\;(t_{0})*r_{x}\;(t_{10})}{r_{\mathrm{wt}}\;(t_{10})*r_{x}\;(t_{0})}}{g_{\mathrm{wt}}}+1,$$

where *r_x_*(*t*_0_) is the fraction of strain *X* in the population at time *i* and *g_wt_* is the number of doublings of wildtype growth in the experiment. *g_wt_* is calculated from OD measurements. For this screen, *r*_wt_(*t*_i_) is calculated as the median value of nonessential sgRNAs.

Experimental samples with a median NE gene knockdown |L2FC| > 0.6 were discarded. Additionally, replicates with Pearson’s *r* < 0.8 with the other two replicates of the same condition were discarded. These criteria generally corresponded with bottleneck by low sample read depth.

Calculated RF values were then corrected for doubling artifacts as described previously (112). While we documented the number of doublings for each experiment, various technical errors and chemical effects can still preclude accurate doubling counts. Minimal and glucose complete media experiments were excluded from the correction, as there was no appropriate reference condition.

A small number of nonessential genes (<3%) were excluded from further analysis because the two sgRNAs targeting them had discrepant median RFs across experiments (at least one median RF > 0.9, difference in median RF > 0.1), indicating possible toxicity or off-target effects of one sgRNA and precluding a reliable phenotype. We took advantage of polar effects to salvage sgRNAs from a small number of genes: If one sgRNA had a high maximum correlation with another sgRNA targeting within the same operon (r > 0.5) and the other did not (maximum correlation with an operon-targeting sgRNA < 0.3), the highly correlated sgRNA was salvaged, and the other was discarded (Dataset S3). We note that several of the genes with discrepant sgRNAs share operons with essential genes, resulting in variable fitnesses due to polar effects.

### Calculation of Chemical–Gene S-Scores.

For nonessential genes, S-scores were calculated as described below. For essential genes and those targeted by a library of mismatch sgRNAs, sgRNAs were binned into three bins by strength (low, medium, and high knockdown efficiency), and S-scores for each bin were calculated separately.

RFs were transformed into S-scores as previously described (23). First, we took the median fitness of each gene knockdown across all conditions, and its dispersion about the median using the median absolute deviation (MAD) times 1.4826 to align with SD. As applied previously, a floor was assigned to MADS; if a gene knockdown MAD was below the median MAD, it was replaced with the median MAD. Each RF was normalized by subtracting the gene knockdown RF median and scaled by the calculated MAD. For calculating S-scores, mean and variance for each gene–chemical interaction was calculated from all fitness values of relevant sgRNAs in each replicate of the given condition (e.g., for a nonessential gene targeted by two sgRNAs, in a condition with three technical replicates, mean and variance was calculated from the 6 RF measurements). These values were used for all gene–gene and condition–condition correlation analyses.

## Supplementary Material

Appendix 01 (PDF)

Dataset S01 (XLSX)

Dataset S02 (PDF)

Dataset S03 (XLSX)

Dataset S04 (XLSX)

Dataset S05 (CSV)

Dataset S06 (XLSX)

Dataset S07 (XLSX)

Dataset S08 (XLSX)

## Data Availability

Raw sequencing data are deposited on NCBI-SRA Bioproject PRJNA1375834 (113). All study data are included in the article and/or supporting information.
