# Supplementary material for: The phenotypic landscape of the model firmicute Bacillus subtilis
Source: Proc Natl Acad Sci U S A. 2026 Sep 29;123(40):e2619440123. doi: 10.1073/pnas.2619440123 (PMC13643137; doi:10.1073/pnas.2619440123)
Supplement: Supplementary file 1 [file pnas.2619440123.sapp.pdf]

SI Appendix for: The phenotypic landscape of the model firmicute *Bacillus subtilis*

Authors: Lili M. Kim[1], Byoung-Mo Koo[1], Hannah N. Burkhart[1], Daniela Vollmer[4], Waldemar Vollmer[4], Horia Todor[1,*], Carol A. Gross[1,2,3,*]

Affiliations:

[1]Department of Microbiology and Immunology, University of California, San Francisco, San Francisco, CA 94158, USA

[2]Department of Cell and Tissue Biology, University of California, San Francisco, San Francisco, CA 94158, USA.

[3]California Institute of Quantitative Biology, University of California, San Francisco, San Francisco 94158, CA, USA.

[4]Institute for Molecular Bioscience, The University of Queensland, Brisbane, Australia

*Correspondence: horia.todor@gmail.com and cgrossucsf@gmail.com

SUPPLEMENTAL METHODS

Comparison to the STRING Database

Comparison to the STRING database was performed by downloading the *Bacillus subtilis* 168-specific interactions scores from http://string-db.org/ (1). Scores across all channels were included in the analysis. Correlations between genes in the same operon were eliminated from the analysis.

Disk Diffusion Assays

Disk diffusion assays were performed as described in (2). Briefly, 200uL of OD 1.0 culture was mixed with 4mL of 1% LB agar and evenly spread across an LB agar plate. 7mm filter discs were placed on the top agar and 3-5uL of antibiotic stock solution was applied to each disc.

Suppressor Mapping

Suppressors of aztreonam sensitivity (Δ*ybfF*) or essentiality on LB (Δ*yneF*) were grown overnight in LB. Genomic DNA was extracted using the DNeasy Blood and Tissue Kit (Qiagen #69504). Whole-genome sequencing and variant calling was performed by SeqCenter in Pittsburgh, PA.

AlphaFold3 Modelling

The webserver of AlphaFold3 (3) was used to predict atomic models of the YneF dimer, YbfF dimer, and $EcfA_1/EcfA_2$/EcfT/YybS complex. Jobs were run with default options.

Analysis of peptidoglycan composition

Cell wall purification and muropeptide analysis were performed as previously described in ref (4). Briefly, exponentially growing *B. subtilis* cells were harvested by centrifugation, the cell pellet resuspended in PBS and the suspension added dropwise into boiling 5% SDS solution. The crude cell walls were recovered by high-speed centrifugation, washed free of SDS, broken with glass beads in a Precellys homogenizer and incubated with DNase, RNase, and trypsin. The trypsin was inactivated by adding 1% SDS and incubating at 80°C. The cell walls were thoroughly washed with water and freeze-dried.

For removal of the wall teichoic acid, the cell wall was resuspended with 48% hydrofluoric acid, stirred at 4°C for 48 h and washed several times, resulting in purified peptidoglycan. The

muropeptides (disaccharide peptide subunits) were released by digestion with the muramidase cellosyl (Hoechst, Germany), reduced with sodium borohydride (5 mg/ml in sodium borate buffer pH 9.0) and separated by HPLC (Shimadzu CBM 40) using a reversed phase Prontosil 120-3-C18-AQ 3 μM column (Bischoff) and a linear gradient from 100% solvent A (40 mM sodium phosphate, pH 4.5, 0.0003% sodium azide) to 100% solvent B (40 mM sodium phosphate, 20% methanol, pH 4.0) at 52°C, in 270 min. Muropeptides were detected at 202 nm and peaks identified by comparison with ref (4, 5).

Bacterial Two-Hybrid (BACTH)

BACTH was performed as described in ref (6). Briefly, plasmids derived from pUT18 and pKT25 containing T18 and T25 fusions to *B. subtilis* proteins were transformed into a *cya-E. coli* strain and selected on LB agarose containing kanamycin (40μg/mL) and carbenicillin (100μg/mL). Resulting transformants were pinned on maltose minimal agarose containing kanamycin, carbenicillin, and X-gal (40μg/mL) and incubated for 2 days at 30°C. Growing on maltose minimal media provides a highly specific readout: only cells with a functional adenylate cyclase, resulting from association of T18 and T25 by the interaction of their two fused proteins, are able to utilize maltose and grow.

SigM reporter assay

Luciferase reporter construction and measurement was performed as previously described (7). Luciferase measurements without antibiotic stress were performed by inoculating 1uL of mid-log cells into 99uL of fresh LB in a 96 well plate. Luciferase measurements with antibiotic stress were performed by adding 100uL of exponentially growing cells (OD ~0.1) and the antibiotic to a 96 well plate. Plates were incubated at 37°C with shaking in a Spark Multimode Microplate reader (Tecan), and OD600 and luminescence were measured every 5 minutes. Promoter activity was normalized to "relative luminescence" by dividing the relative light units (RLU) by OD600. Assays were conducted with three biological replicates.

High-throughput growth assays

All strains were grown to OD ~1.0, then back-diluted 1:100 into 200 μL fresh LB (for induced growth curves, LB was supplemented with 1% xylose) and grown with shaking at 37°C in a Synergy H4 plate reader (BioTek) for 10 hours. OD600 was measured at 5-min intervals. Assays were conducted with three technical replicates unless otherwise noted.

Conditioned media experiments

When selecting strains for conditioned media experiments, we prioritized species which were easily accessible, grew to saturation in LB culture, and could reasonably be found in the soil. To make conditioned media, each species or strain was grown overnight in LB (bacteria) or TSB (yeast and a *B. subtilis* 168 control) at 37°C or 30°C to saturation. Cultures were spun down and supernatant was passed through a 0.22 µm PVDF filter to remove cells. Conditioned media was frozen at -80°C until use. For the screen, conditioned media was diluted to 50% or 10% final concentration with fresh media, whichever produced ~90% WT growth rate of the library.

Growth assays of the library in conditioned media experiments were performed largely as described in "*Chemical genomics experiments and sequencing.*" Briefly, the library was thawed, diluted to OD600 0.01 and grown in appropriate media (LB or TSB) at 37°C to an OD600 of 0.10. A t_0 sample was taken and the culture was back diluted to OD600 0.01 in 37°C diluted conditioned media with 1% xylose to induce dCas9 expression. After approximately 5 doublings, the culture was back diluted 1:10 into fresh diluted conditioned media + 1% xylose; after another 5 doublings, the culture was pelleted and frozen. Sample preparation was performed as described in "*Chemical genomics experiments and sequencing.*"

Data Retrieval and Processing for Crossfeeding Comparisons

B. subtilis, arrayed. Per-gene RF and normalized RF (nRF) values were taken from Table S4 of ref (8). The KanR library data was used. The nRF of the KanR library on glucose ammonium minimal medium ("nRF_KanR_glucose+NH4+") was multiplied by the RF on LB ("RF_KanR_LB37°C") to remove normalization before plotting.

E. coli, pooled. Per-gene L2FC data was retrieved from https://fit.genomics.lbl.gov/ (9). L2FC values were averaged from the two D-Glucose experiments (set1IT003 and set1IT004) before plotting.

E. coli, arrayed. Per-gene fitness scores were taken from Dataset S1 of ref (10). Fitness scores from the Glucose (M9) condition reanalyzed from the original 2011 dataset (11) ("M9min glucose [0.2% (w/v)] {0}") were plotted.

Crossfeeding assays

Crossfeeding assays were performed as previously described (12). Briefly, a WT *B. subtilis* “donor” strain was struck onto a MM agar plate and incubated at 37°C. After 3 hours, an auxotrophic recipient strain was struck 3mm from the donor in parallel. Photos were taken after 48 hours of incubation.

SUPPLEMENTAL FIGURES AND LEGENDS

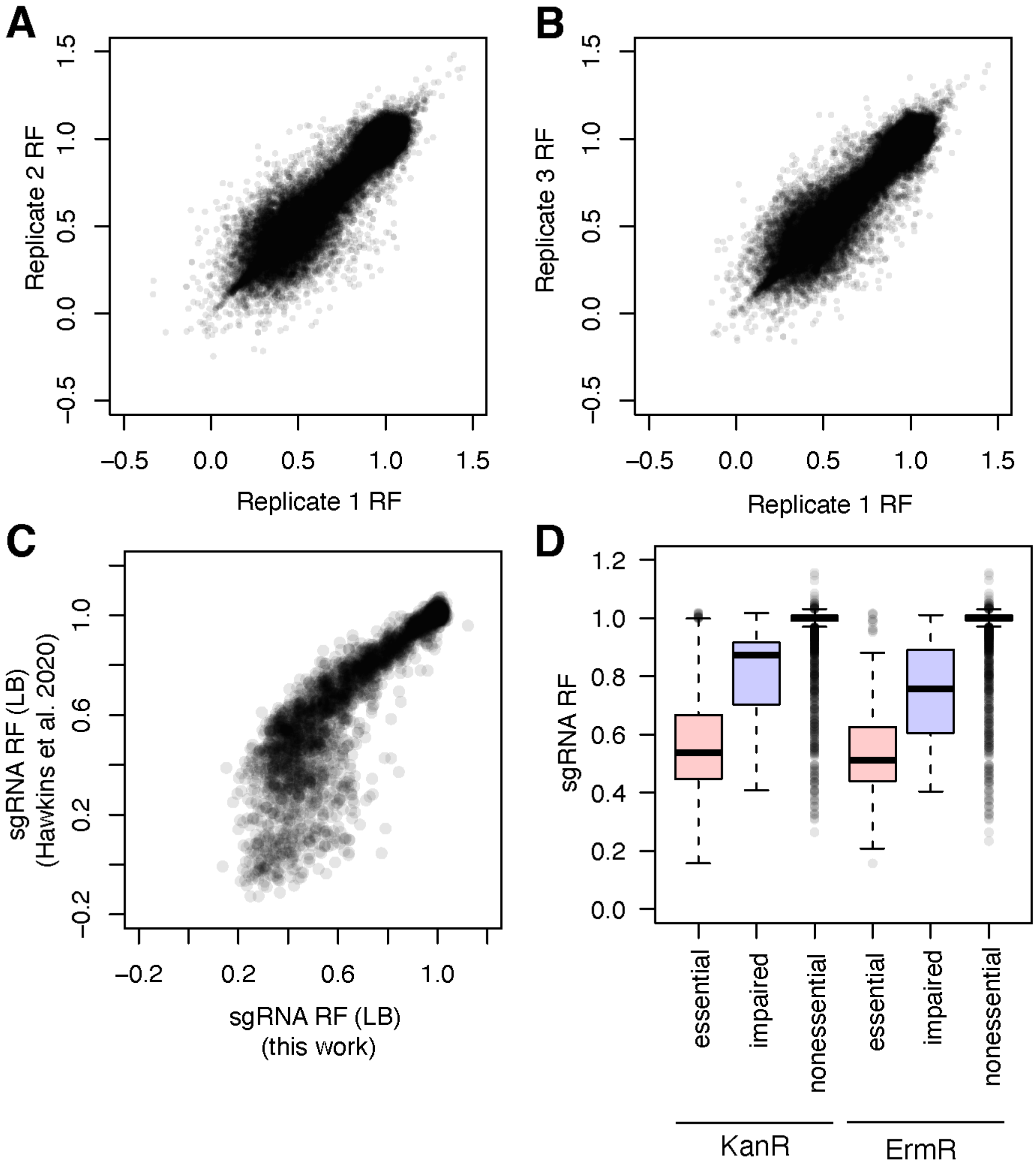

Fig. S1. RF of sgRNAs is reproducible and consistent with previous measurements of sgRNA- or mutant fitness.

A-B. RFs are reproducible across conditions. RF of all sgRNAs plotted pairwise from each experimental replicate of every condition (done in triplicate): 11,559 sgRNAs x 149 conditions ≈

1.7 million RF values per replicate. Pearson's *r* for all comparisons > 0.95. Condition replicates with severely abnormal median sgRNA behavior and/or outlier correlation values to the other triplicates generally indicate bottlenecking from read depth and were excluded during preprocessing prior to this analysis (Methods). 100,000 randomly chosen data points are displayed (out of approximately 1.7 million).

C. RF of sgRNAs shared between our library and ref (13) are consistent. sgRNAs were selected from ref (13). Lower minimum RF in ref (13) reflects a higher sequencing depth in that study. Pearson's *r* = 0.88.

D. RF of fully-complementary sgRNAs targeting genes designated as "nonessential", "impaired" or "essential" based on their single gene deletion phenotypes in KanR or ErmR libraries from ref (8). Box bounds: first and third quartile, midline: median, whiskers: most extreme datapoints. sgRNAs targeting nonessential genes with RF < 0.9 may reflect polar knockdown effects on essential operon partners.

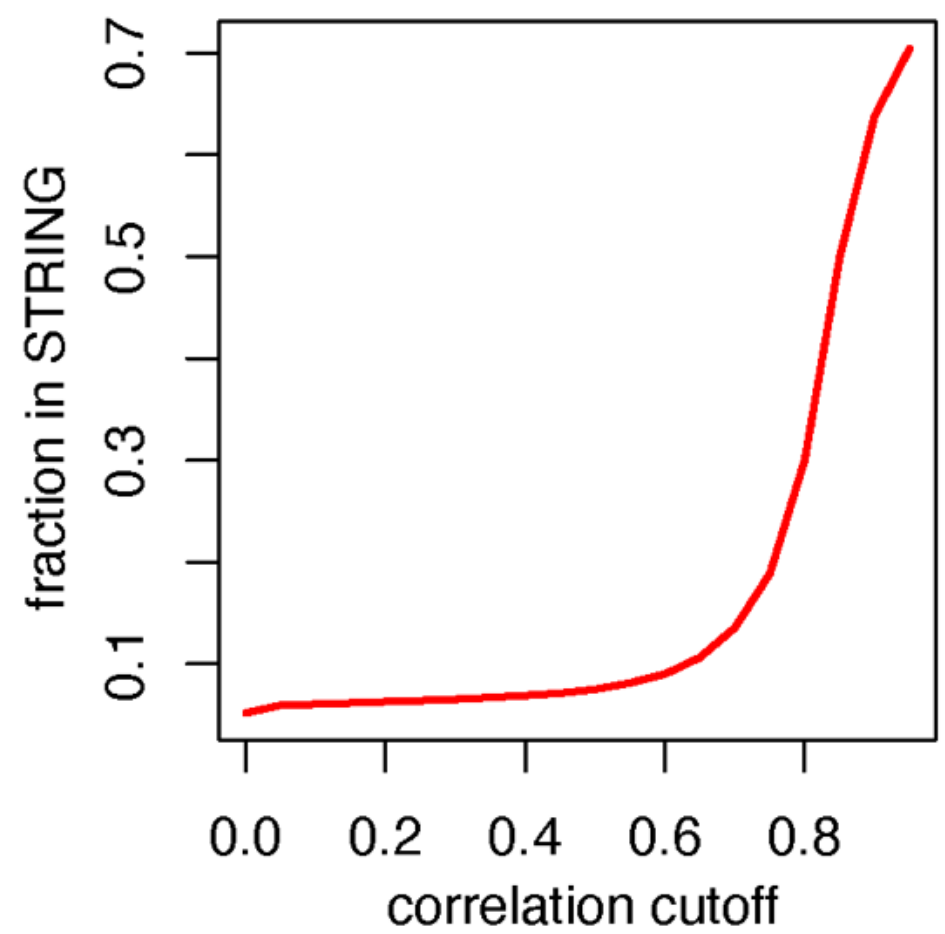

Fig. S2. Correlations between the phenotypic profiles of genes reflect shared function. High-correlation gene pairs in the screen are enriched for STRING interactions. Intra-operon interactions (n=13k) are excluded due to CRISPRi polar effects. For genes with multiple knockdown bins, the maximum correlation between the bin sets was taken. STRING interaction scores are calculated from experimental and database datasets (Methods).

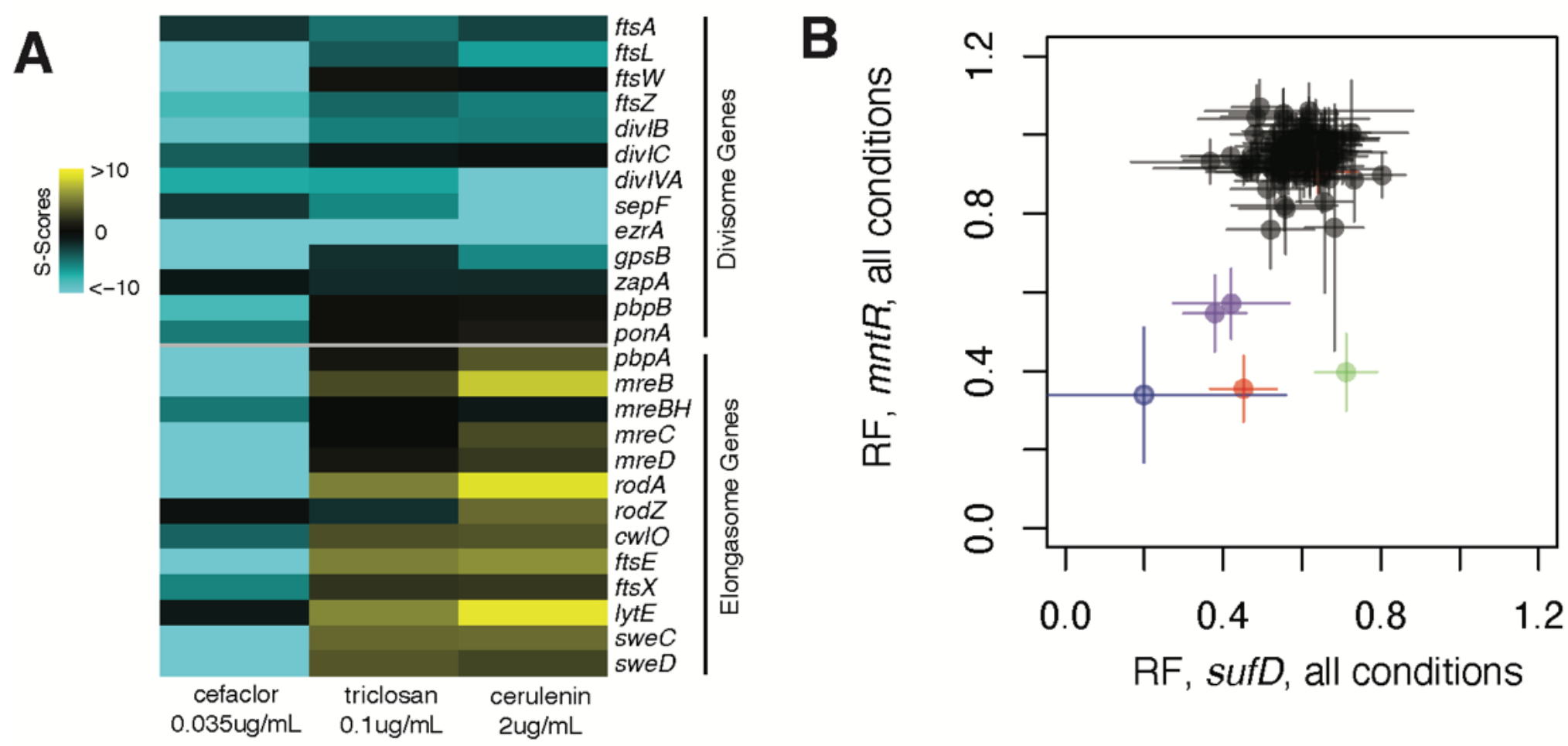

Fig. S3. Expanded insight into previously identified pathway-pathway interactions.

A. Gene-chemical S-Scores of elongasome or divisome genes against the PG synthesis inhibitor cefaclor and the FAS inhibitors cerulenin and triclosan. As expected, knockdown of genes encoding the PG synthesis machineries of both the elongasome and divisome are sensitized to the β-lactam cefaclor. In contrast, inhibition of FAS by triclosan or cerulenin generally had a protective effect on knockdown of elongasome genes, but a sensitizing effect on knockdown of divisome genes. Drug concentrations with strongest S-scores are displayed. For genes targeted by multiple knockdown bins, only the most responsive bin (greatest sum of absolute S-scores across the drug concentrations used) is shown.

B. RF values of *mntR* knockdown and *sufD* knockdown across all conditions. For *sufD*, the average RF of the two fully complementary sgRNAs is plotted. Red = mupirocin, Blue = dipyridyl (iron chelator), Green = excess manganese, Purple = minimal and glucose complete media.

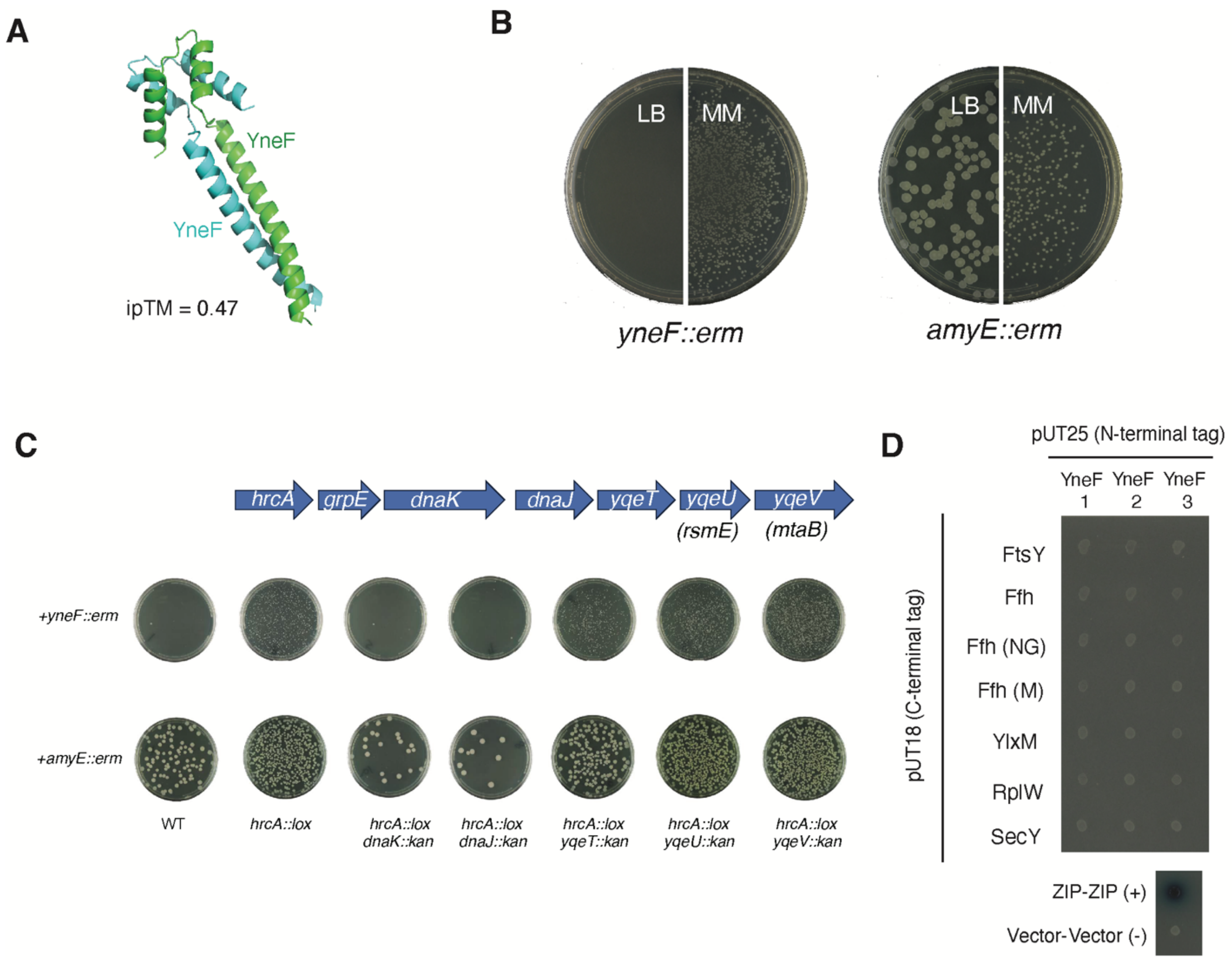

Fig. S4. Additional data regarding YneF.

A. YneF modeled as a dimer by AlphaFold3 (ipTM = 0.47).

B. Transformation of Δ*yneF::erm* or a Δ*amyE::erm* control into WT onto LB and MM. Plates were grown 72hr at 37°C before being photographed. Viable Δ*yneF::erm* transformants are produced when plating on MM but not LB.

C. DnaKJ is required for suppression of *yneF* phenotype. Double deletions of *hrcA* and each gene in the *hrcA* operon were transformed with either Δ*yneF::erm* or an Δ*amyE::erm* control. Only the Δ*hrcA*Δ*dnaK* and Δ*hrcA*Δ*dnaJ* double deletions produced no viable colonies when transformed with Δ*yneF::erm*. We were not able to perform this assay with *groES-groEL* because they are essential.

D. Bacterial two-hybrid (BACTH) assay of N-terminally tagged YneF and C-terminally tagged SRP components. Proteins are tagged with either the T18 or T25 domains of *B. pertussis* adenylate cyclase and plated on minimal maltose media + Xgal (Methods, (6)). Three biological

replicates are shown. Improper insertion of YneF into the membrane due to the N-terminal tag likely precludes the positive interactions observed in C-terminally tagged YneF (Fig. 3D).

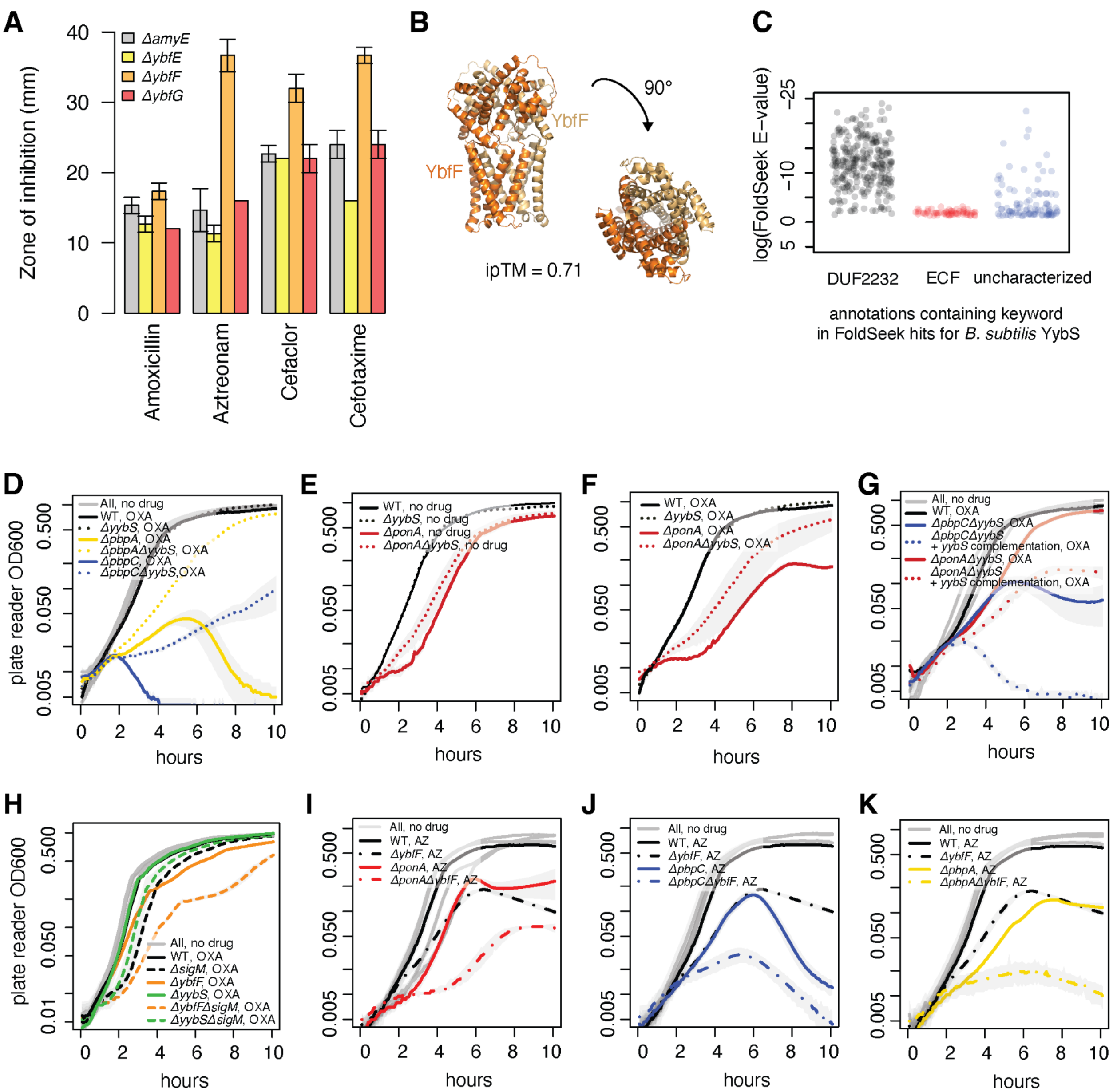

Fig. S5. Additional data regarding *ybfF* and *yybS.*

A. Zone of inhibition from disk diffusion assays of *ΔybfE*, *ΔybfF, ΔybfG*, and an *ΔamyE* control against four β-lactams: 30μg aztreonam, 5μg cefaclor, 5μg amoxicillin, 5μg cefotaxime. Measurement is the diameter across the zone of inhibition (including filter disk). Results indicate *ybfF* is solely responsible for phenotypes.

B. Predicted AlphaFold3 model of YbfF, a polytopic membrane protein. YbfF is predicted to act as a dimer (iptM = 0.71).

C. FoldSeek results for the DUF2232 protein *B. subtilis* YybS grouped by annotation. FoldSeek was run with default parameters.
D. Deletion of *yybS* rescues β-lactam sensitivity of *ΔpbpC* and *ΔpbpA*. Overnight cultures were diluted 1:100 into LB (grey, all strains) or LB + 0.04 µg/ml oxacillin (OXA).
E. Deletion of *yybS* rescues β-lactam sensitivity of *ΔponA*. Overnight cultures were diluted 1:100 into LB + 0.04 µg/ml oxacillin (OXA).
F. Deletion of *yybS* enhances growth of *ΔponA*. Overnight cultures were diluted 1:100 into LB.
G. Deletion of *yybS* rescues β-lactam sensitivity of PBP deletions. This effect is reversed when *yybS* is exogenously expressed, indicating that this is due to the presence of YybS and not to a polar effect. Mid-log cultures of WT, *ΔpbpCΔyybS* (*ΔpbpCΔyybS* + P_{xyl}-*yybS* without inducer), *ΔpbpCΔyybS* + *yybS* complementation (*ΔpbpCΔyybS* + P_{xyl}-*yybS* + 1% xylose induction), *ΔponAΔyybS* (*ΔponA* + P_{xyl}-*yybS* without inducer), and *ΔponAΔyybS* (*ΔponA* + P_{xyl}-*yybS* + 1% xylose induction) were diluted 1:100 in LB only (grey, all strains) or LB + 0.04 µg/ml oxacillin (OXA).
H. Genetic interactions between *sigM*, *ybfF*, and *yybS*. *ΔsigMΔybfF* double deletion is more sensitive to β-lactams than single deletions alone. Deletion of *yybS* rescues β-lactam sensitivity of *ΔsigM*. WT, *ΔybfF*, *ΔyybS, ΔsigM*, *ΔsigMΔybfF,* and *ΔsigMΔyybS* were grown from a 1:100 dilution in LB only (grey, all strains) or LB + 0.04 µg/ml oxacillin (OXA).
I-K. Deletion of *ybfF* exacerbates β-lactam sensitivity of *ΔponA, ΔpbpC,* and *ΔpbpA*. Overnight cultures were diluted 1:100 into LB (grey, all strains) or LB + 3 µg/ml aztreonam (AZ).

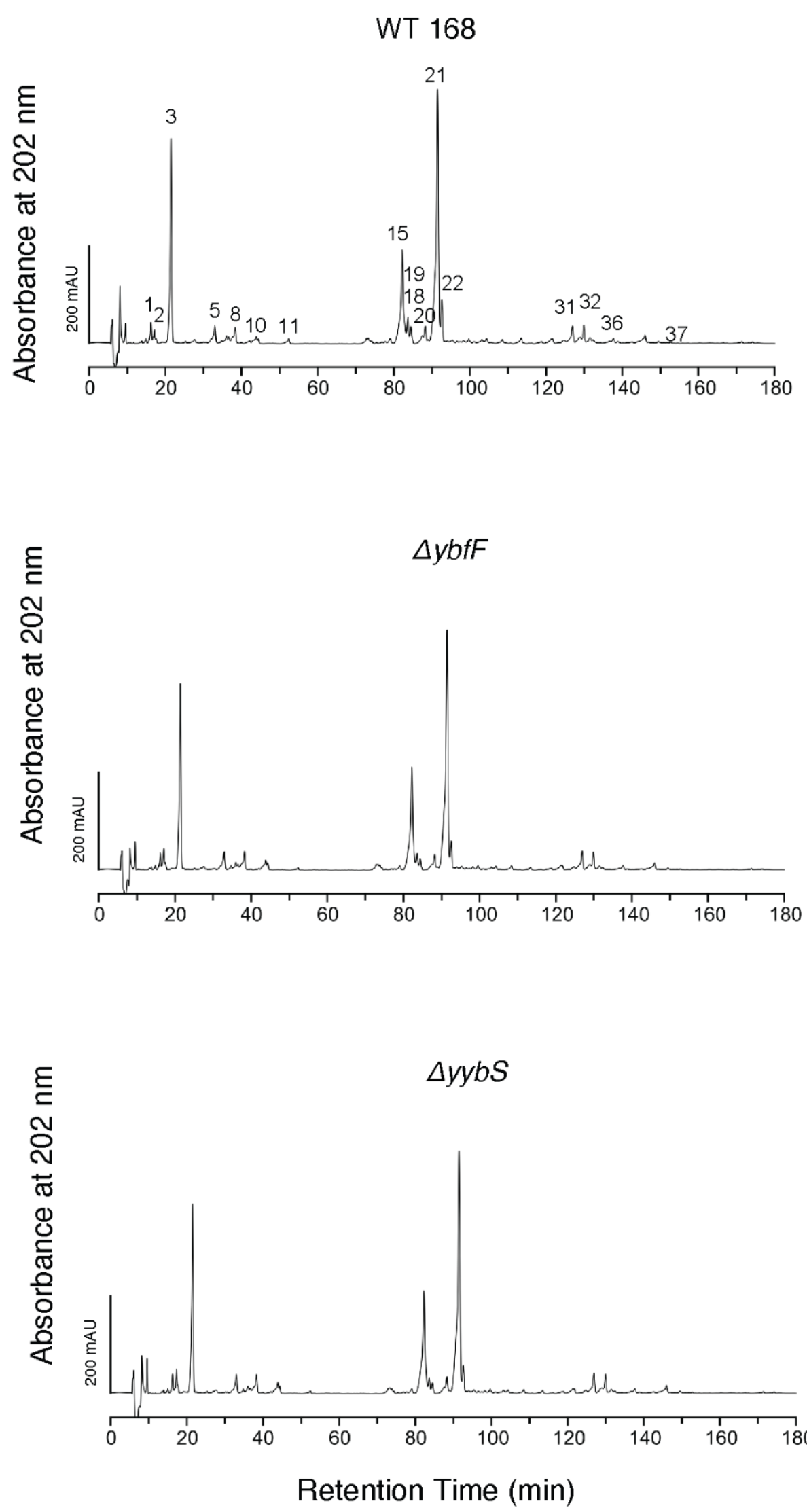

Peak #	Muropeptide
1	Tri
2	Tri (NH2) (PO4)
3	Tri (NH2)
5	Di
6	Tri-Ala-mDAP (NH2)
7	Tetra (NH2)
8	Tri-Ala-mDAP (NH2)2
10	TriTetra(-GM)(NH2)2
11	Penta (NH2)
15	TriTetra (NH2)
18	TriTetra (NH2) (deAc)
19	TriTetra (NH2) (deAc)
20	TriTetra (NH2)
21	TriTetra (NH2)2
22	TriTetra (NH2)2 (deAc)
31	TriTetraTetra (NH2)3
32	TriTetraTetra (NH2)3 (deAc)
36	TriTetra(Anh) (NH2)2
37	TriTetraTetraTetra (NH2)4

Fig. S6. Representative HPLC samples of muropeptides isolated from WT, *ΔybfF*, and *ΔyybS B. subtilis.*

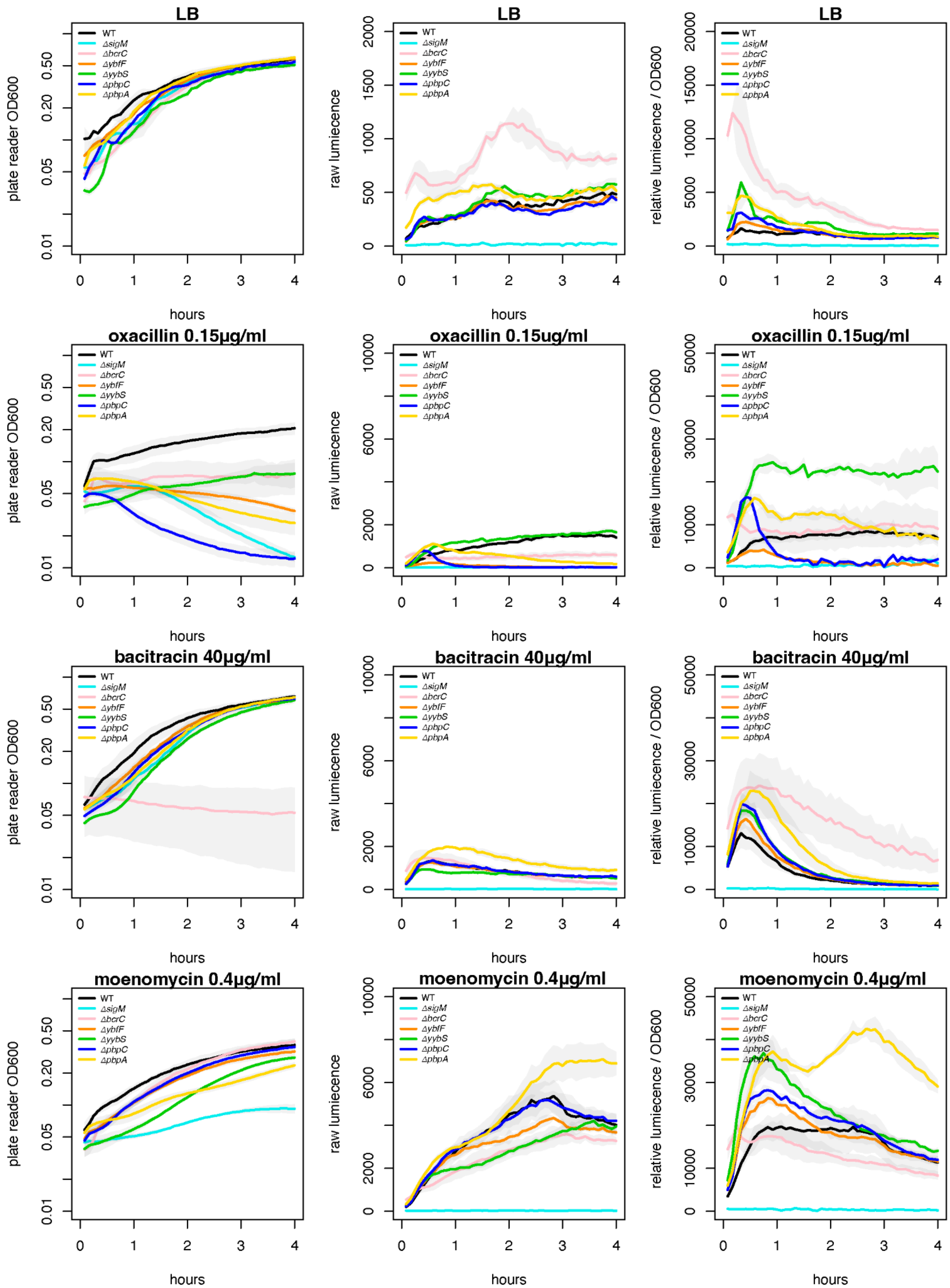

LB
plate reader OD600
raw lumiecence
relative lumiecence / OD600
oxacillin 0.15µg/ml
oxacillin 0.15ug/ml
bacitracin 40µg/ml
moenomycin 0.4µg/ml
hours
WT
ΔsigM
ΔbcrC
ΔybfF
ΔyybS
ΔpbpC
ΔpbpA
0.01
0.05
0.20
0.50
0
500
1000
1500
2000
2000
6000
10000
5000
10000
15000
20000
10000
30000
50000
0
1
2
3
4

Fig. S7. Expanded SigM reporter data. OD600, raw luminescence, and relative luminescence (raw luminescence / OD600) over 4 hours of growth of WT, *ΔbcrC*, *ΔsigM*, *ΔpbpA, ΔpbpC,* or *ΔybfF* mutants plus a P_{lux}-SigM reporter (Methods). Strains were diluted into LB + 0.15μg/mL oxacillin, LB, LB + 40μg/mL bacitracin, or LB + 0.4μg/mL moenomycin in a 96-well plate and OD600 and luminescence were measured (Methods).

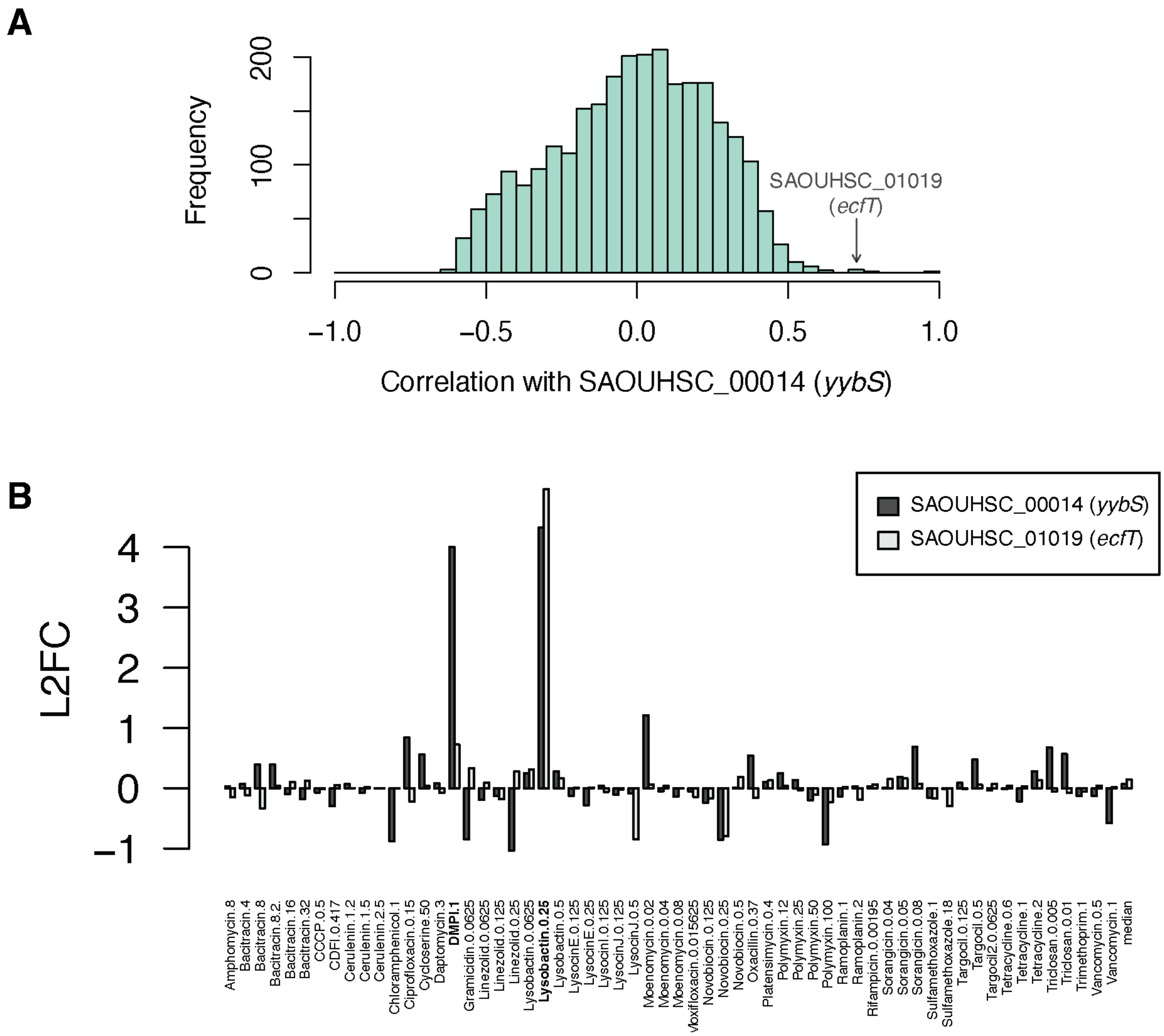

Fig. S8. YybS protection phenotypes against cell wall drugs are conserved in *S. aureus***.**

A. Gene-gene correlations with *yybS* (SAOUHSC_00014) across all conditions in an *S. aureus* Tn-seq chemical genomics screen (14). *ecfT* (SAOUHSC_01019) is highly correlated with *yybS*, suggesting that *yybS* acts with ECF transporters in *S. aureus.*

B. L2FC of *S. aureus* transposon deletions of *yybS* (SAOUHSC_00014) and *ecfT* (SAOUHSC_01019) across all conditions in a *S. aureus* Tn-seq chemical genomics screen (14). Deletion of *yybS* protects against lysobactin, which inhibits PG synthesis by binding Lipid II, and DPMI, which prevents export of Lipid II to the cell surface (14).

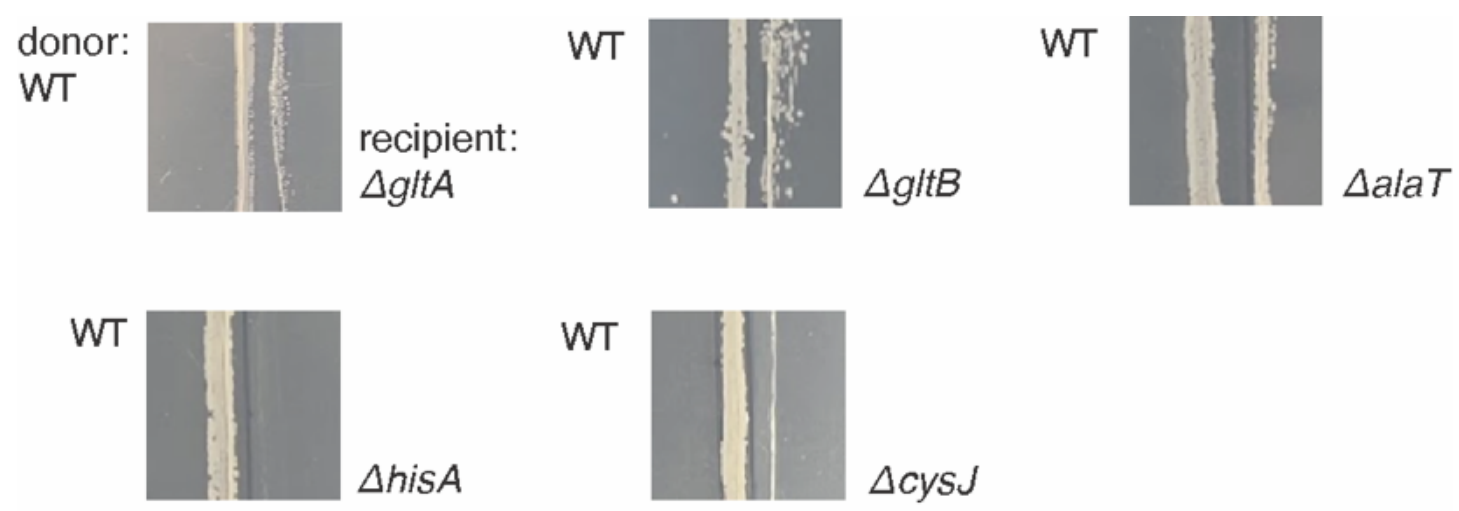

Fig. S9. Crossfeeding of PG components. The “donor” strain (WT) was struck onto MM agar and incubated at 37°C. After 3 hours, the auxotrophic recipient strain was struck ~3mm from the donor in parallel (Methods) and showed crossfeeding at this distance in glutamate and alanine auxotrophs but not *ΔhisA* or *ΔcysJ*. No evidence of crossfeeding for these strains was apparent in arrayed data (8). Photos were taken after 48 hours of incubation.

DATASETS WITH TITLES AND LEGENDS

Dataset S1 Information about primers, strains, and all stress conditions used.

Dataset S2 Per-gene knockdown-fitness curves for all essential or impaired genes (defined in Methods, “sgRNA library design”) targeted by a mismatch sgRNA library in all conditions. Conditions with a combined |S| > 20 across sgRNA bins are highlighted.

Dataset S3 RF for all sgRNAs across all conditions passing quality control.

Dataset S4 S-scores for all genes across all conditions passing quality control.

Dataset S5 Correlations between all genes across conditions.

Dataset S6 Mapped suppressor mutations for *yneF*, *ybfF,* and *ponA* knockout strains.

Dataset S7 Foldseek e-values for YybS.

Dataset S8 pH values of post-dilution bacterial conditioned media.

REFERENCES

1. D. Szklarczyk, *et al.*, STRING v10: protein–protein interaction networks, integrated over the tree of life. *Nucleic Acids Res.* **43**, D447–D452 (2015).
2. Y. Luo, J. D. Helmann, Analysis of the role of Bacillus subtilis σM in β-lactam resistance reveals an essential role for c-di-AMP in peptidoglycan homeostasis. *Mol. Microbiol.* **83**, 623–639 (2012).
3. J. Abramson, *et al.*, Accurate structure prediction of biomolecular interactions with AlphaFold 3. *Nature* **630**, 493–500 (2024).
4. J. Sassine, J. Sousa, M. Lalk, R. A. Daniel, W. Vollmer, Cell morphology maintenance in Bacillus subtilis through balanced peptidoglycan synthesis and hydrolysis. *Sci. Rep.* **10**, 17910 (2020).
5. A. Atrih, G. Bacher, G. Allmaier, M. P. Williamson, S. J. Foster, Analysis of Peptidoglycan Structure from Vegetative Cells of Bacillus subtilis 168 and Role of PBP 5 in Peptidoglycan Maturation. *J. Bacteriol.* **181**, 3956–3966 (1999).
6. D. Ladant, Lipopolysaccharide Transport, Methods and Protocols. *Methods Mol. Biol.* **2548**, 145–167 (2022).
7. H. Zhao, D. M. Roistacher, J. D. Helmann, Aspartate deficiency limits peptidoglycan synthesis and sensitizes cells to antibiotics targeting cell wall synthesis in Bacillus subtilis. *Mol. Microbiol.* **109**, 826–844 (2018).
8. B.-M. Koo, *et al.*, Construction and Analysis of Two Genome-Scale Deletion Libraries for Bacillus subtilis. *Cell Syst* **4**, 291-305.e7 (2017).
9. M. N. Price, *et al.*, Mutant phenotypes for thousands of bacterial genes of unknown function. *Nature* **557**, 503–509 (2018).
10. A. L. Shiver, *et al.*, A Chemical-Genomic Screen of Neglected Antibiotics Reveals Illicit Transport of Kasugamycin and Blasticidin S. *Plos Genet* **12**, e1006124 (2016).
11. R. J. Nichols, *et al.*, Phenotypic Landscape of a Bacterial *Cell*. Cell **144**, 143–156 (2011).
12. K. R. Sidiq, M. W. Chow, Z. Zhao, R. A. Daniel, Alanine metabolism in Bacillus subtilis. *Mol. Microbiol.* **115**, 739–757 (2021).
13. J. S. Hawkins, *et al.*, Mismatch-CRISPRi Reveals the Co-varying Expression-Fitness Relationships of Essential Genes in Escherichia coli and Bacillus subtilis. *Cell Syst* **11**, 523-535.e9 (2020).
14. M. Santiago, *et al.*, Genome-wide mutant profiling predicts the mechanism of a Lipid II binding antibiotic. *Nat Chem Biol* **14**, 601–608 (2018).
