# Supplementary material for: The phenotypic landscape of the model firmicute Bacillus subtilis
Source: Proc Natl Acad Sci U S A. 2026 Sep 29;123(40):e2619440123. doi: 10.1073/pnas.2619440123 (PMC13643137; doi:10.1073/pnas.2619440123)
Supplement: Supplementary file 2 [file pnas.2619440123.sd02.pdf]

Dataset S2. Landscapes for Essential and Impaired genes
“Essential” and “Impaired” genes defined by parameters specified in “sgRNA library design” section in Methods.
Conditions where |sum of S-scores across bins| > 20 are highlighted by color.
Lines are colored by roughly-grouped condition types (see key below).
Line type of colored lines (dashed/solid/dotted) is arbitrarily assigned within each plot to distinguish between conditions of the same type.

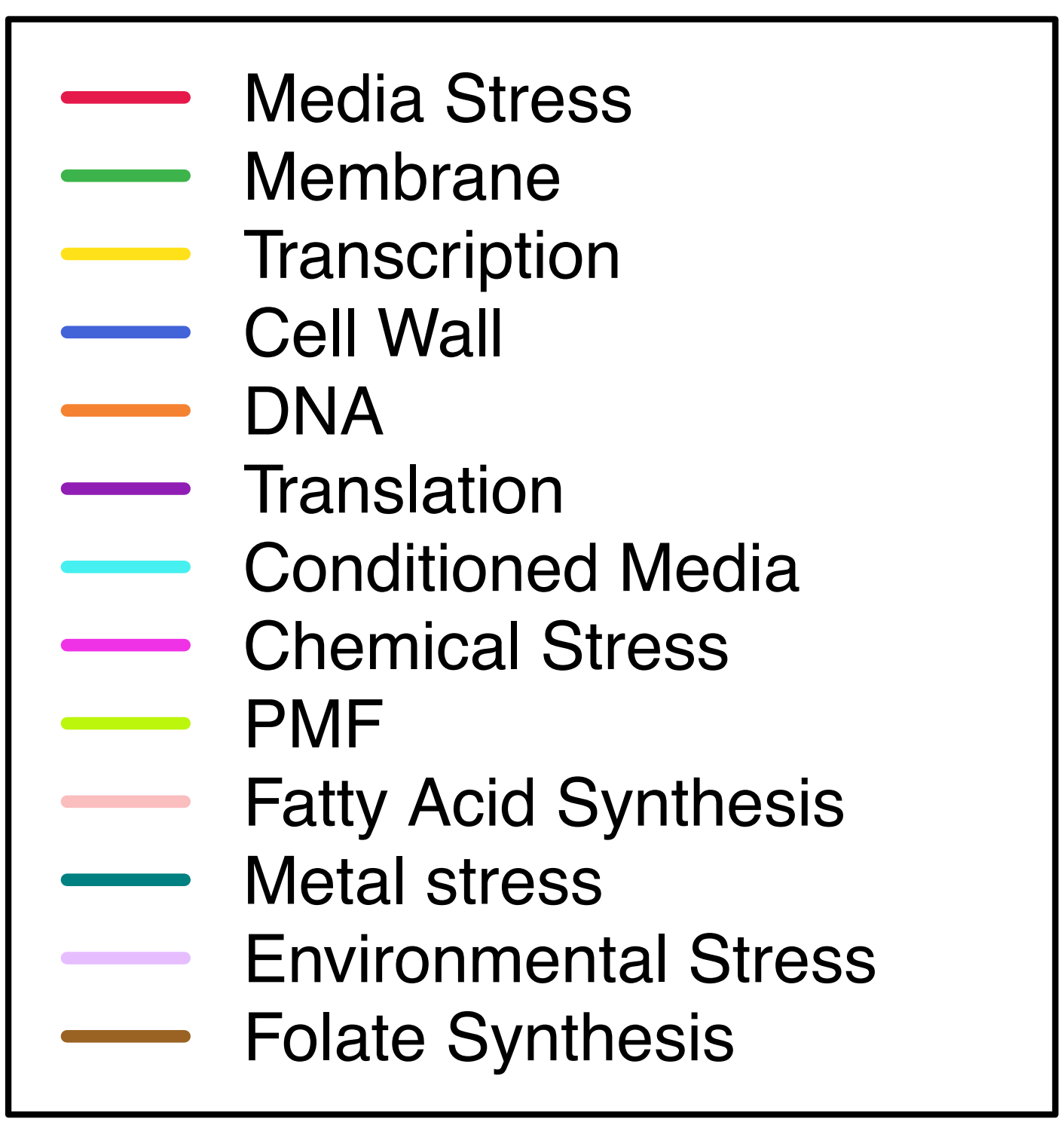

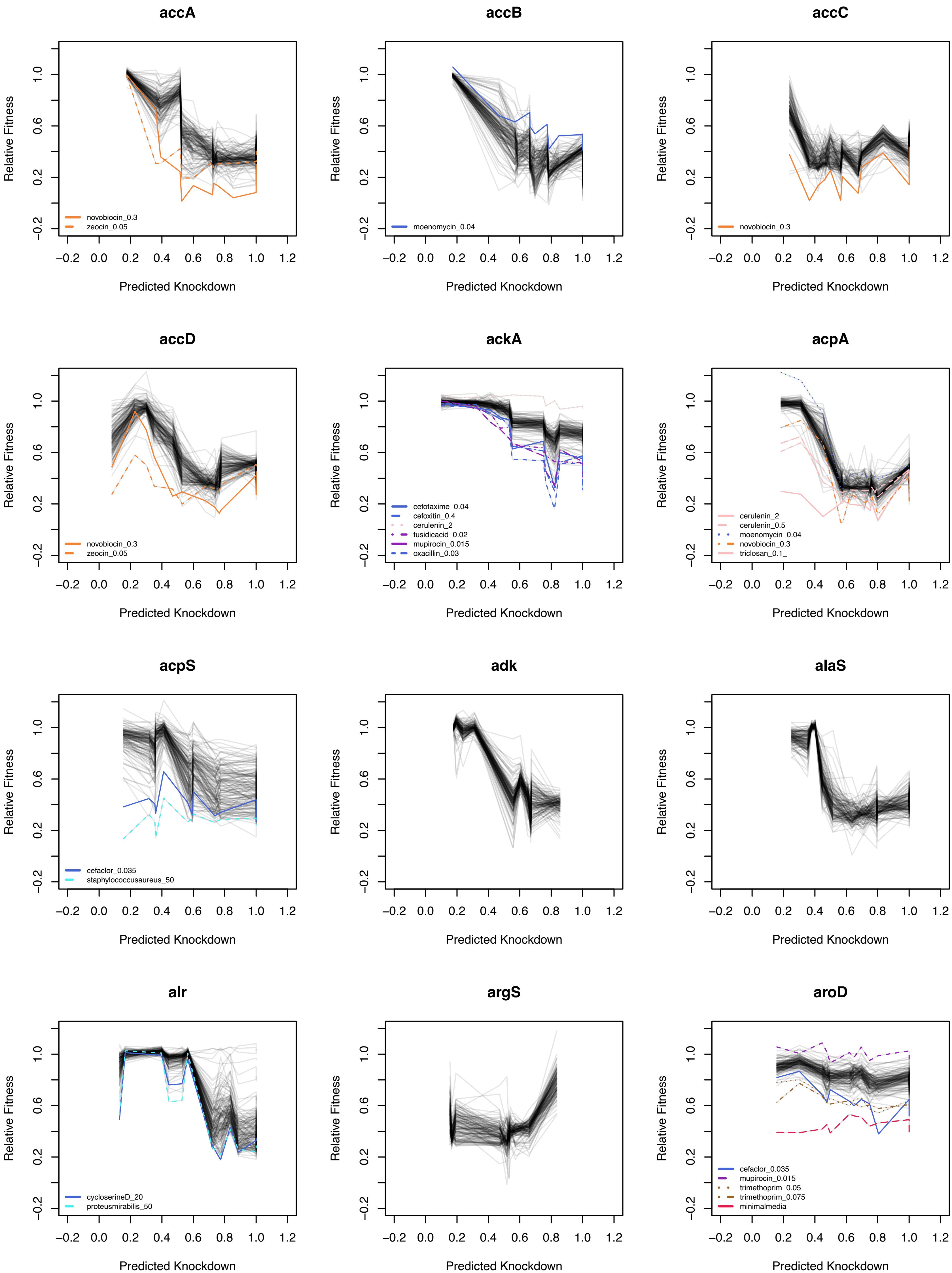

accA
Relative Fitness
Predicted Knockdown
novobiocin_0.3
zeocin_0.05
accB
Relative Fitness
Predicted Knockdown
moenomycin_0.04
accC
Relative Fitness
Predicted Knockdown
novobiocin_0.3
accD
Relative Fitness
Predicted Knockdown
novobiocin_0.3
zeocin_0.05
ackA
Relative Fitness
Predicted Knockdown
cefotaxime_0.04
cefoxitin_0.4
cerulenin_2
fusidicacid_0.02
mupirocin_0.015
oxacillin_0.03
acpA
Relative Fitness
Predicted Knockdown
cerulenin_2
cerulenin_0.5
moenomycin_0.04
novobiocin_0.3
triclosan_0.1_
acpS
Relative Fitness
Predicted Knockdown
cefaclor_0.035
staphylococcusaureus_50
adk
Relative Fitness
Predicted Knockdown
alaS
Relative Fitness
Predicted Knockdown
alr
Relative Fitness
Predicted Knockdown
cycloserineD_20
proteusmirabilis_50
argS
Relative Fitness
Predicted Knockdown
aroD
Relative Fitness
Predicted Knockdown
cefaclor_0.035
mupirocin_0.015
trimethoprim_0.05
trimethoprim_0.075
minimalmedia

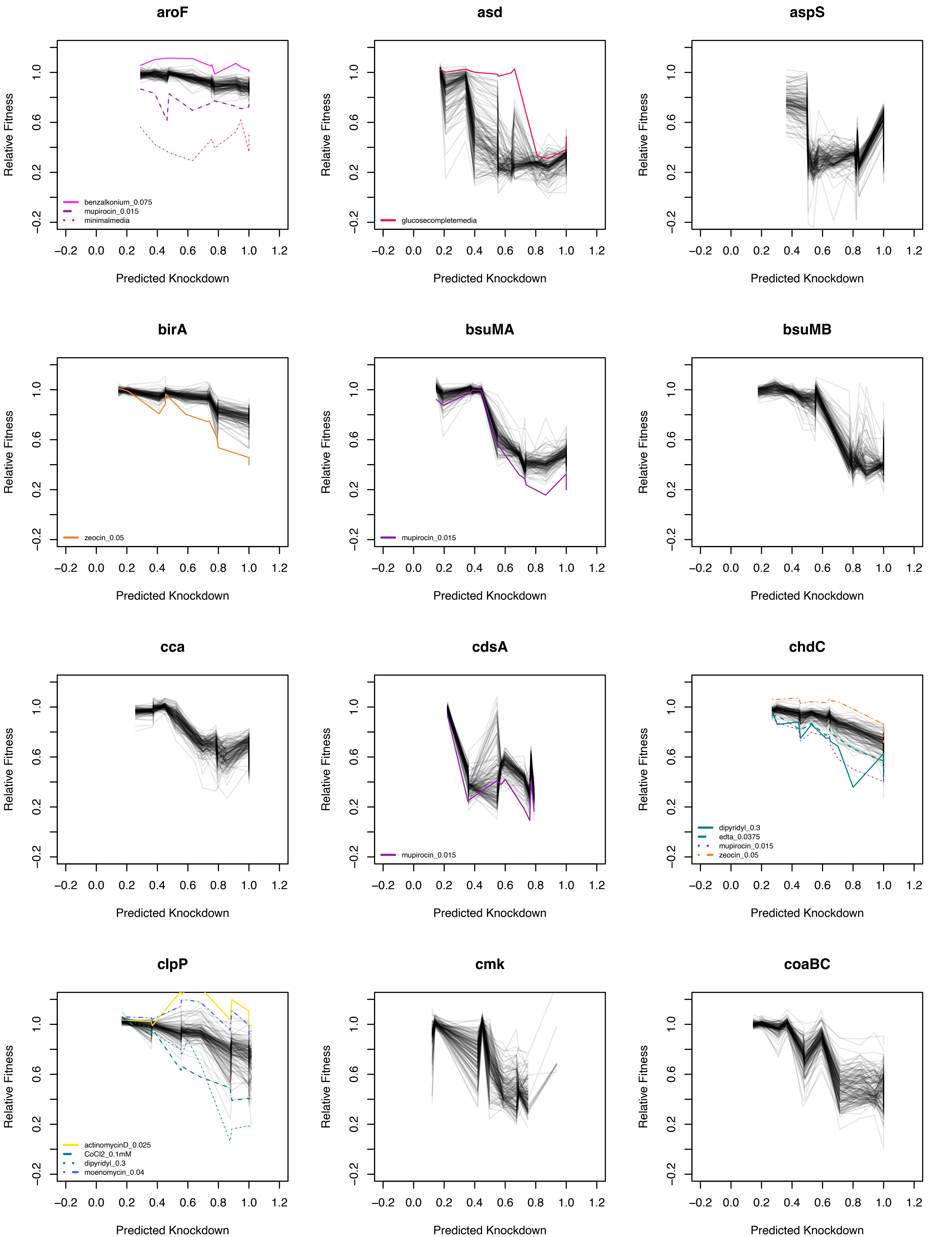
aroF
Relative Fitness
Predicted Knockdown
benzalkonium_0.075
mupirocin_0.015
minimalmedia
asd
glucosecompletemedia
aspS
birA
zeocin_0.05
bsuMA
mupirocin_0.015
bsuMB
cca
cdsA
mupirocin_0.015
chdC
dipyridyl_0.3
edta_0.0375
mupirocin_0.015
zeocin_0.05
clpP
actinomycinD_0.025
CoCl2_0.1mM
dipyridyl_0.3
moenomycin_0.04
cmk
coaBC

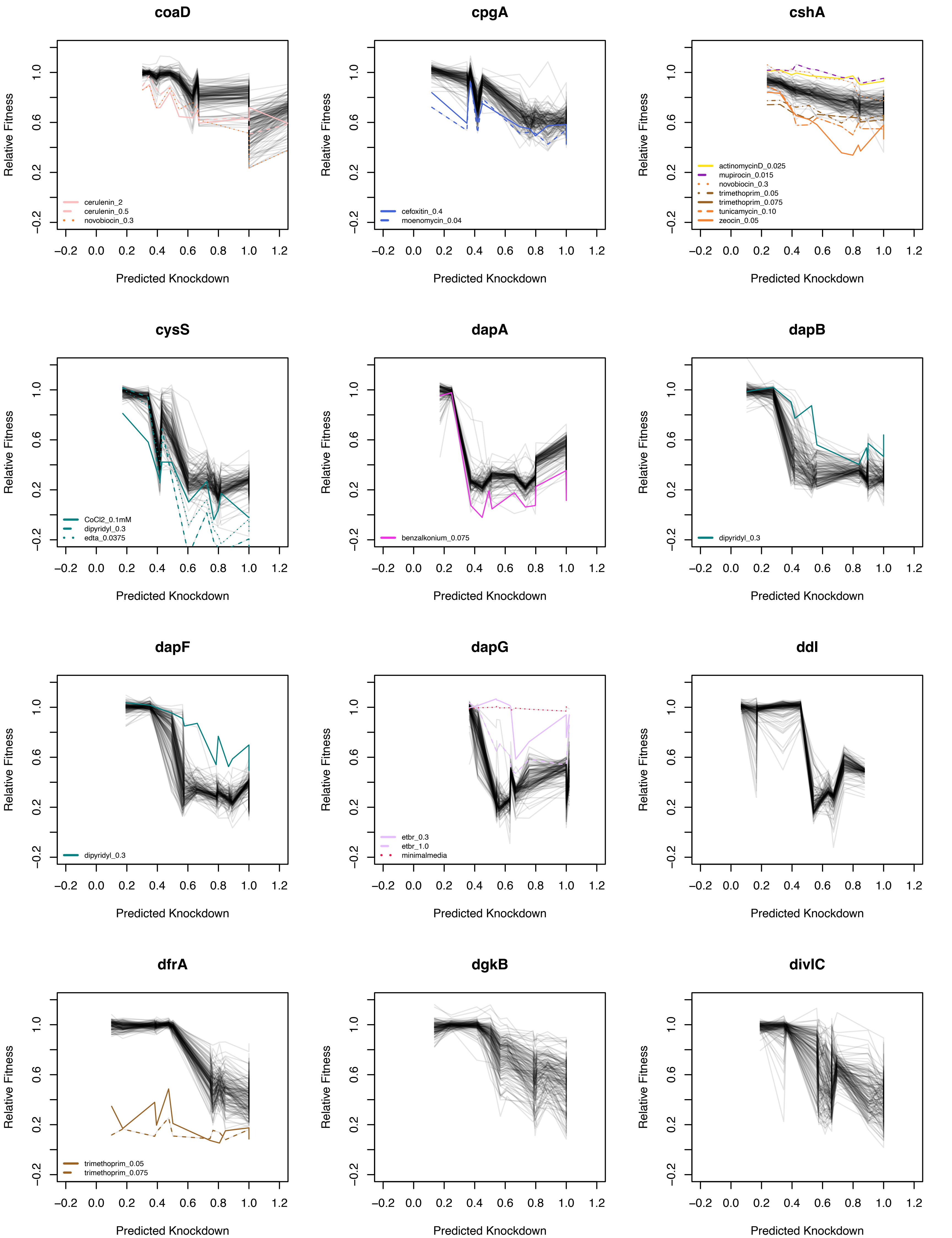
coaD
Relative Fitness
Predicted Knockdown
cerulenin_2
cerulenin_0.5
novobiocin_0.3
cpgA
Relative Fitness
Predicted Knockdown
cefoxitin_0.4
moenomycin_0.04
cshA
Relative Fitness
Predicted Knockdown
actinomycinD_0.025
mupirocin_0.015
novobiocin_0.3
trimethoprim_0.05
trimethoprim_0.075
tunicamycin_0.10
zeocin_0.05
cysS
Relative Fitness
Predicted Knockdown
CoCl2_0.1mM
dipyridyl_0.3
edta_0.0375
dapA
Relative Fitness
Predicted Knockdown
benzalkonium_0.075
dapB
Relative Fitness
Predicted Knockdown
dipyridyl_0.3
dapF
Relative Fitness
Predicted Knockdown
dipyridyl_0.3
dapG
Relative Fitness
Predicted Knockdown
etbr_0.3
etbr_1.0
minimalmedia
ddl
Relative Fitness
Predicted Knockdown
dfrA
Relative Fitness
Predicted Knockdown
trimethoprim_0.05
trimethoprim_0.075
dgkB
Relative Fitness
Predicted Knockdown
divIC
Relative Fitness
Predicted Knockdown

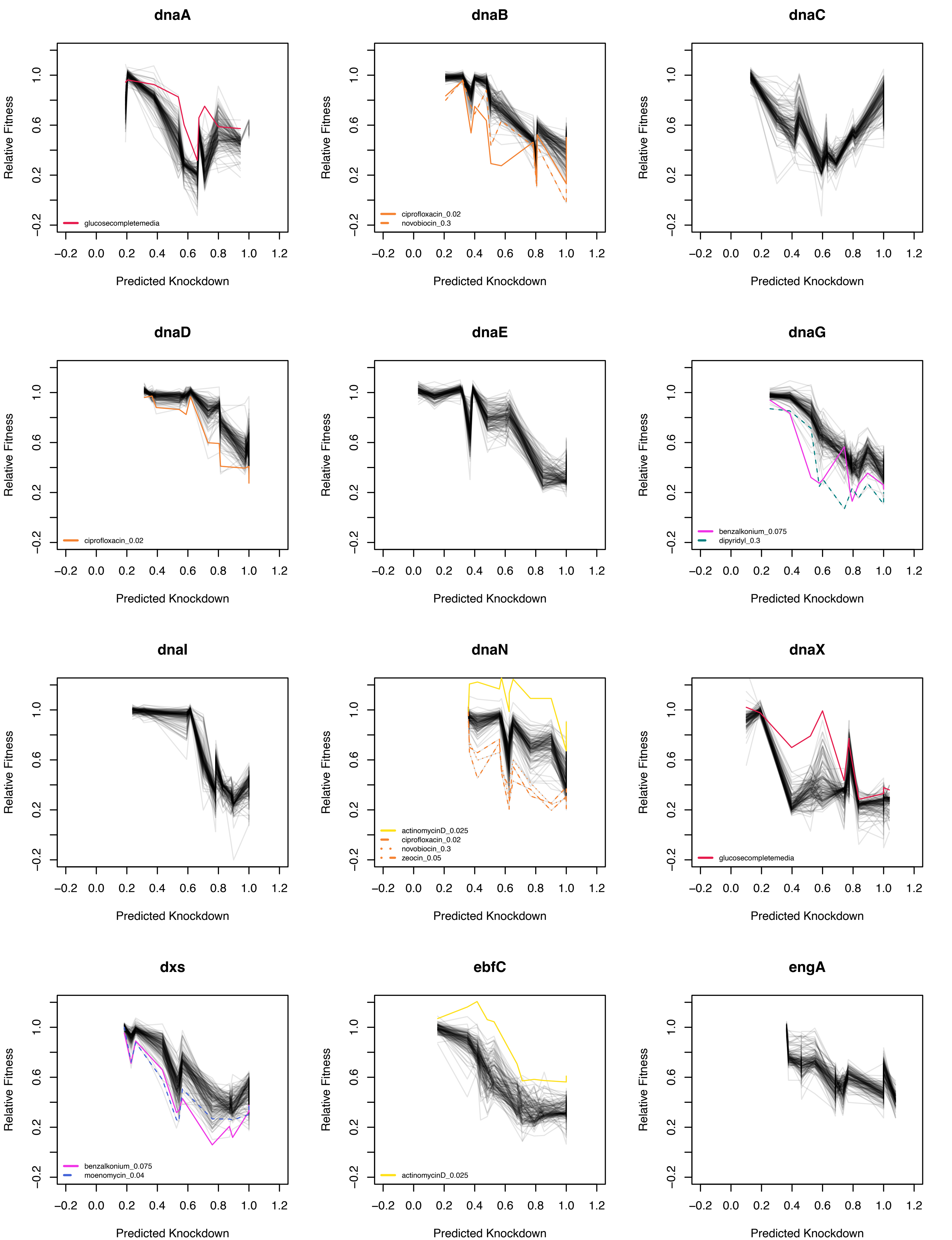
dnaA
glucosecompletemedia
dnaB
ciprofloxacin_0.02
novobiocin_0.3
dnaC
dnaD
ciprofloxacin_0.02
dnaE
dnaG
benzalkonium_0.075
dipyridyl_0.3
dnaI
dnaN
actinomycinD_0.025
ciprofloxacin_0.02
novobiocin_0.3
zeocin_0.05
dnaX
glucosecompletemedia
dxs
benzalkonium_0.075
moenomycin_0.04
ebfC
actinomycinD_0.025
engA
Relative Fitness
Predicted Knockdown
−0.2 0.2 0.6 1.0
−0.2 0.0 0.2 0.4 0.6 0.8 1.0 1.2

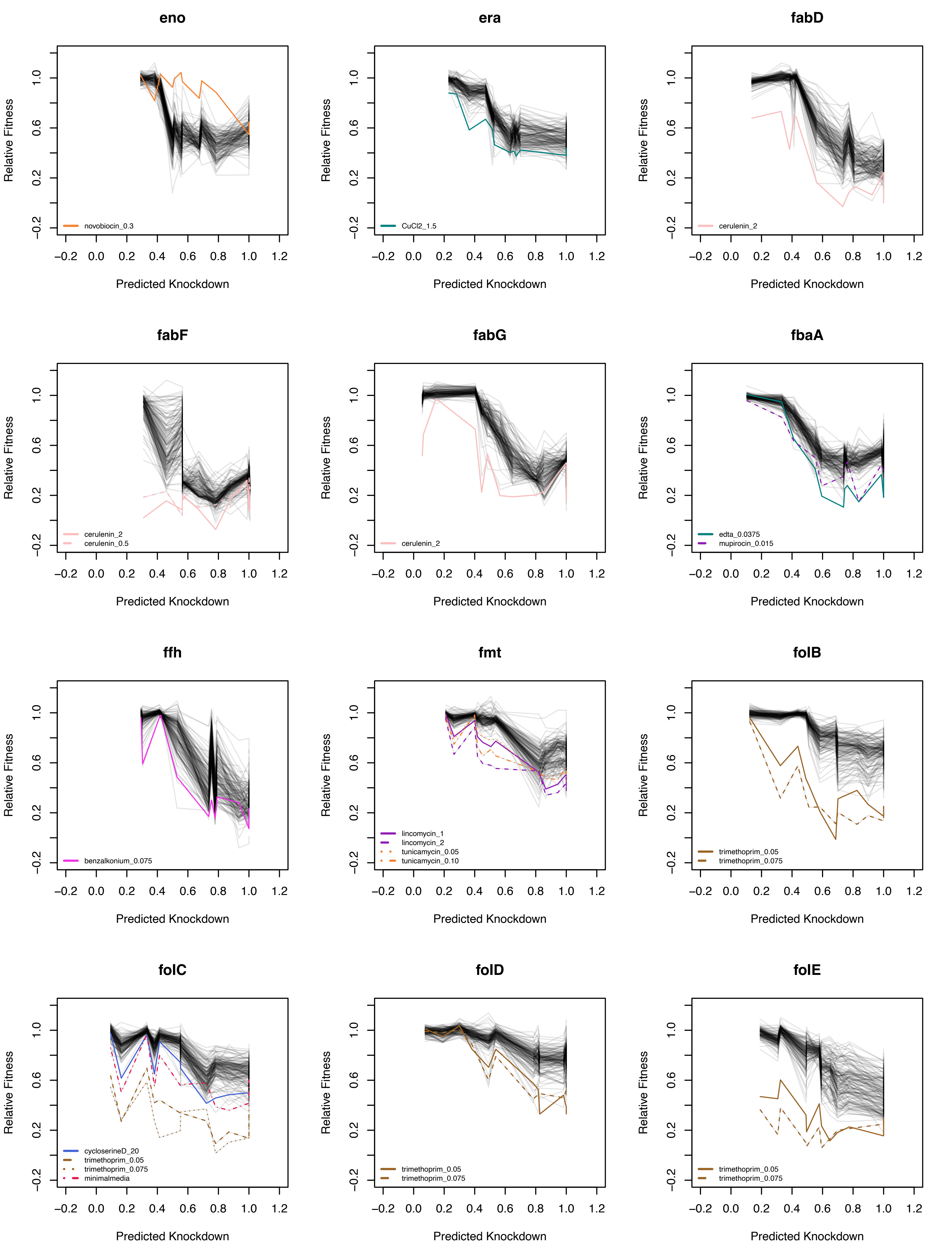
eno
novobiocin_0.3
era
CuCl2_1.5
fabD
cerulenin_2
fabF
cerulenin_2
cerulenin_0.5
fabG
cerulenin_2
fbaA
edta_0.0375
mupirocin_0.015
ffh
benzalkonium_0.075
fmt
lincomycin_1
lincomycin_2
tunicamycin_0.05
tunicamycin_0.10
folB
trimethoprim_0.05
trimethoprim_0.075
folC
cycloserineD_20
trimethoprim_0.05
trimethoprim_0.075
minimalmedia
folD
trimethoprim_0.05
trimethoprim_0.075
folE
trimethoprim_0.05
trimethoprim_0.075
Relative Fitness
Predicted Knockdown
-0.2
0.2
0.6
1.0
-0.2
0.0
0.2
0.4
0.6
0.8
1.0
1.2

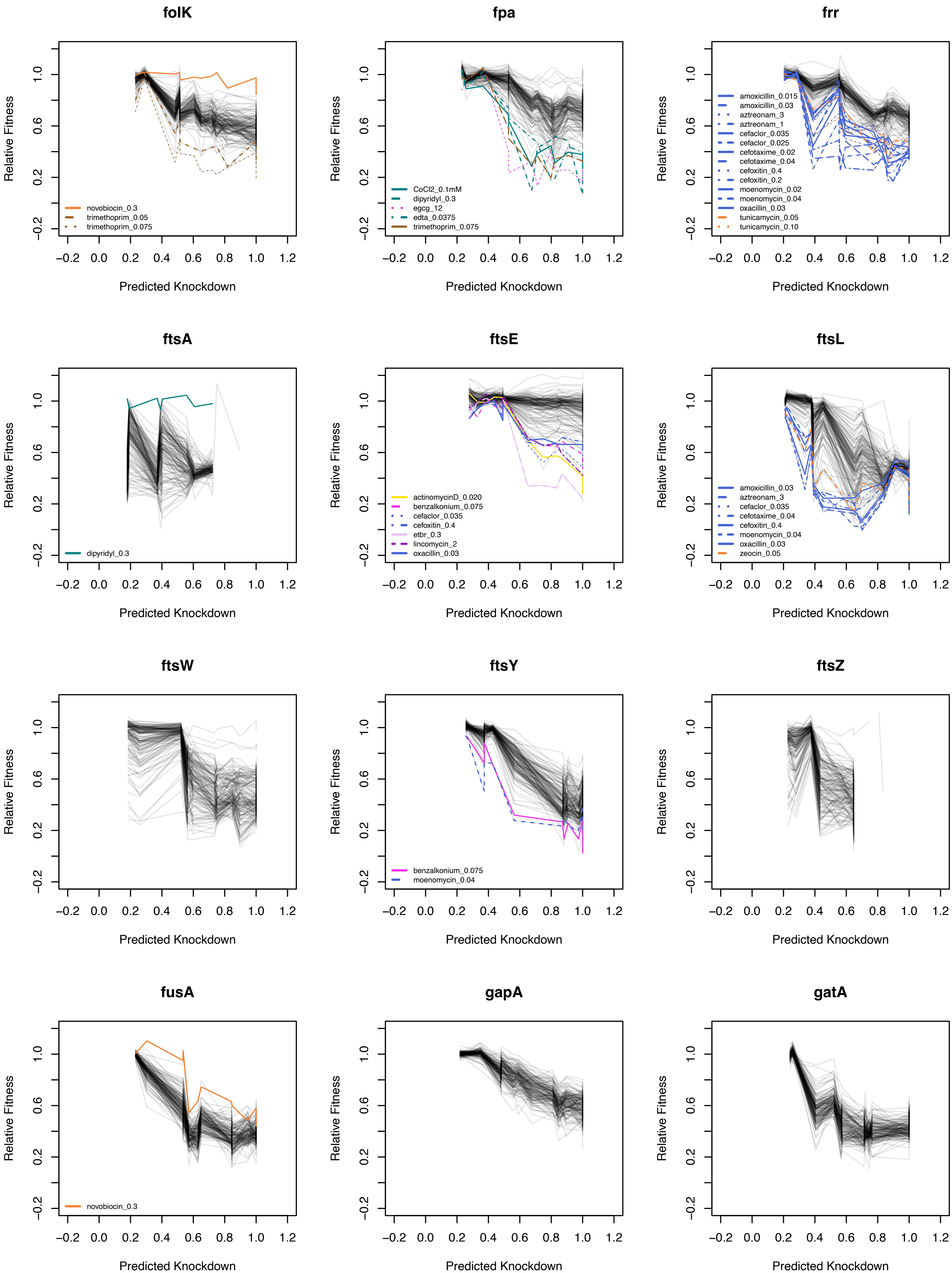

folK
fpa
frr
ftsA
ftsE
ftsL
ftsW
ftsY
ftsZ
fusA
gapA
gatA
Relative Fitness
Predicted Knockdown
novobiocin_0.3
trimethoprim_0.05
trimethoprim_0.075
CoCl2_0.1mM
dipyridyl_0.3
egcg_12
edta_0.0375
trimethoprim_0.075
amoxicillin_0.015
amoxicillin_0.03
aztreonam_3
aztreonam_1
cefaclor_0.035
cefaclor_0.025
cefotaxime_0.02
cefotaxime_0.04
cefoxitin_0.4
cefoxitin_0.2
moenomycin_0.02
moenomycin_0.04
oxacillin_0.03
tunicamycin_0.05
tunicamycin_0.10
dipyridyl_0.3
actinomycinD_0.020
benzalkonium_0.075
cefaclor_0.035
cefoxitin_0.4
etbr_0.3
lincomycin_2
oxacillin_0.03
amoxicillin_0.03
aztreonam_3
cefaclor_0.035
cefotaxime_0.04
cefoxitin_0.4
moenomycin_0.04
oxacillin_0.03
zeocin_0.05
benzalkonium_0.075
moenomycin_0.04
novobiocin_0.3

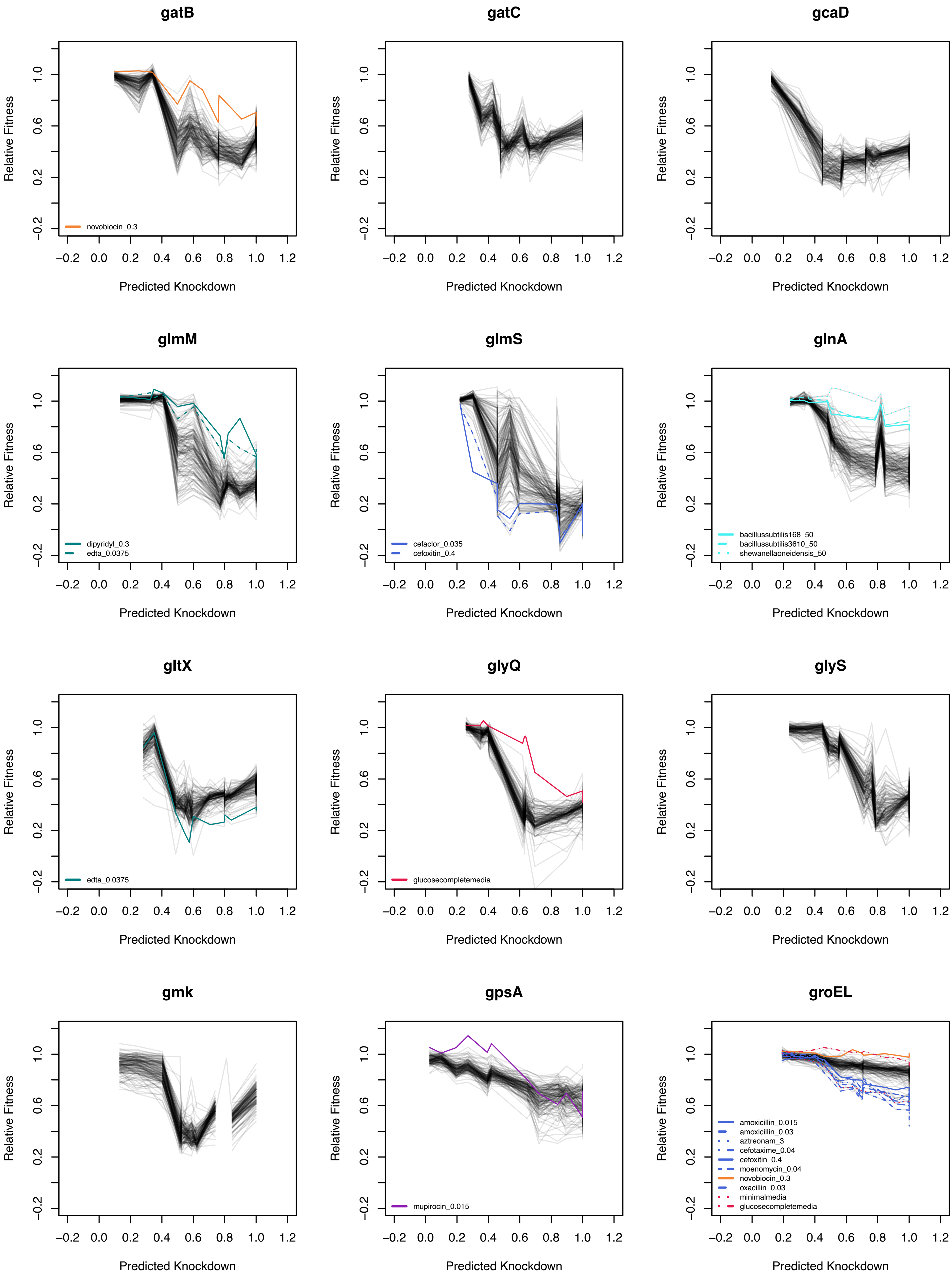
gatB
novobiocin_0.3
gatC
gcaD
glmM
dipyridyl_0.3
edta_0.0375
glmS
cefaclor_0.035
cefoxitin_0.4
glnA
bacillussubtilis168_50
bacillussubtilis3610_50
shewanellaoneidensis_50
gltX
edta_0.0375
glyQ
glucosecompletemedia
glyS
gmk
gpsA
mupirocin_0.015
groEL
amoxicillin_0.015
amoxicillin_0.03
aztreonam_3
cefotaxime_0.04
cefoxitin_0.4
moenomycin_0.04
novobiocin_0.3
oxacillin_0.03
minimalmedia
glucosecompletemedia
Relative Fitness
Predicted Knockdown

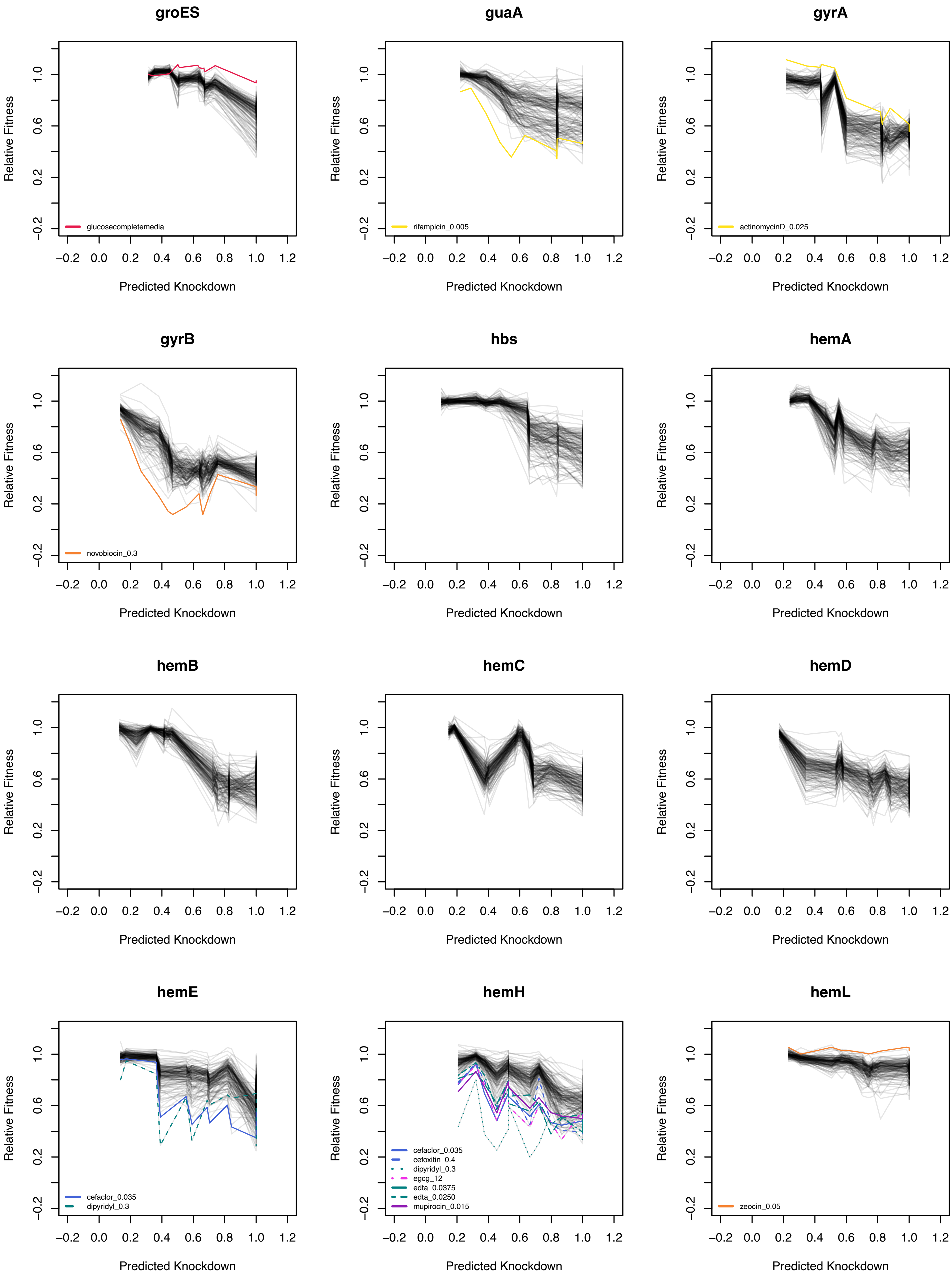
groES
glucosecompletemedia
guaA
rifampicin_0.005
gyrA
actinomycinD_0.025
gyrB
novobiocin_0.3
hbs
hemA
hemB
hemC
hemD
hemE
cefaclor_0.035
dipyridyl_0.3
hemH
cefaclor_0.035
cefoxitin_0.4
dipyridyl_0.3
egcg_12
edta_0.0375
edta_0.0250
mupirocin_0.015
hemL
zeocin_0.05
Relative Fitness
Predicted Knockdown
-0.2
0.2
0.6
1.0
0.0
0.4
0.8
1.2

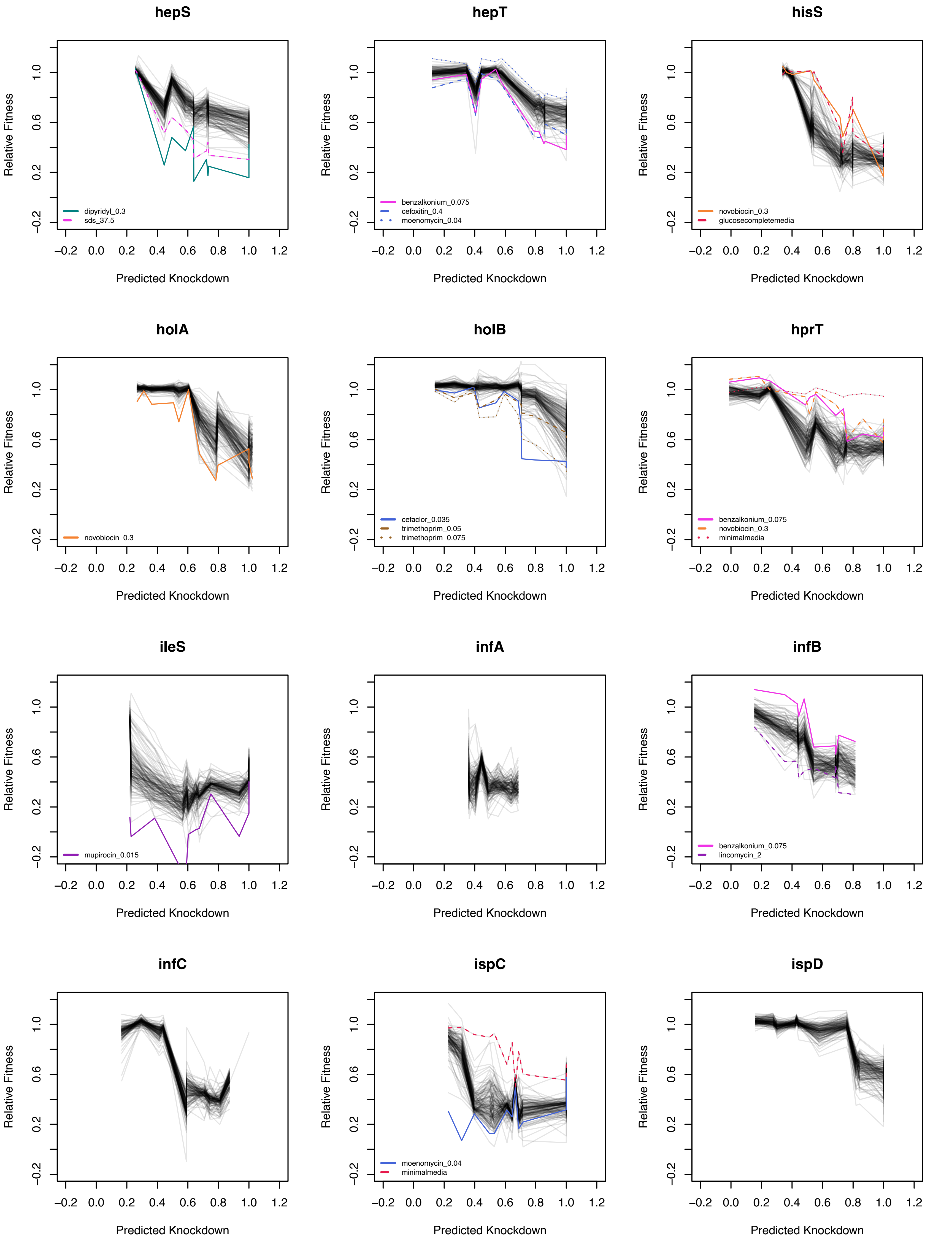
hepS
Relative Fitness
Predicted Knockdown
dipyridyl_0.3
sds_37.5
hepT
Relative Fitness
Predicted Knockdown
benzalkonium_0.075
cefoxitin_0.4
moenomycin_0.04
hisS
Relative Fitness
Predicted Knockdown
novobiocin_0.3
glucosecompletemedia
holA
Relative Fitness
Predicted Knockdown
novobiocin_0.3
holB
Relative Fitness
Predicted Knockdown
cefaclor_0.035
trimethoprim_0.05
trimethoprim_0.075
hprT
Relative Fitness
Predicted Knockdown
benzalkonium_0.075
novobiocin_0.3
minimalmedia
ileS
Relative Fitness
Predicted Knockdown
mupirocin_0.015
infA
Relative Fitness
Predicted Knockdown
infB
Relative Fitness
Predicted Knockdown
benzalkonium_0.075
lincomycin_2
infC
Relative Fitness
Predicted Knockdown
ispC
Relative Fitness
Predicted Knockdown
moenomycin_0.04
minimalmedia
ispD
Relative Fitness
Predicted Knockdown

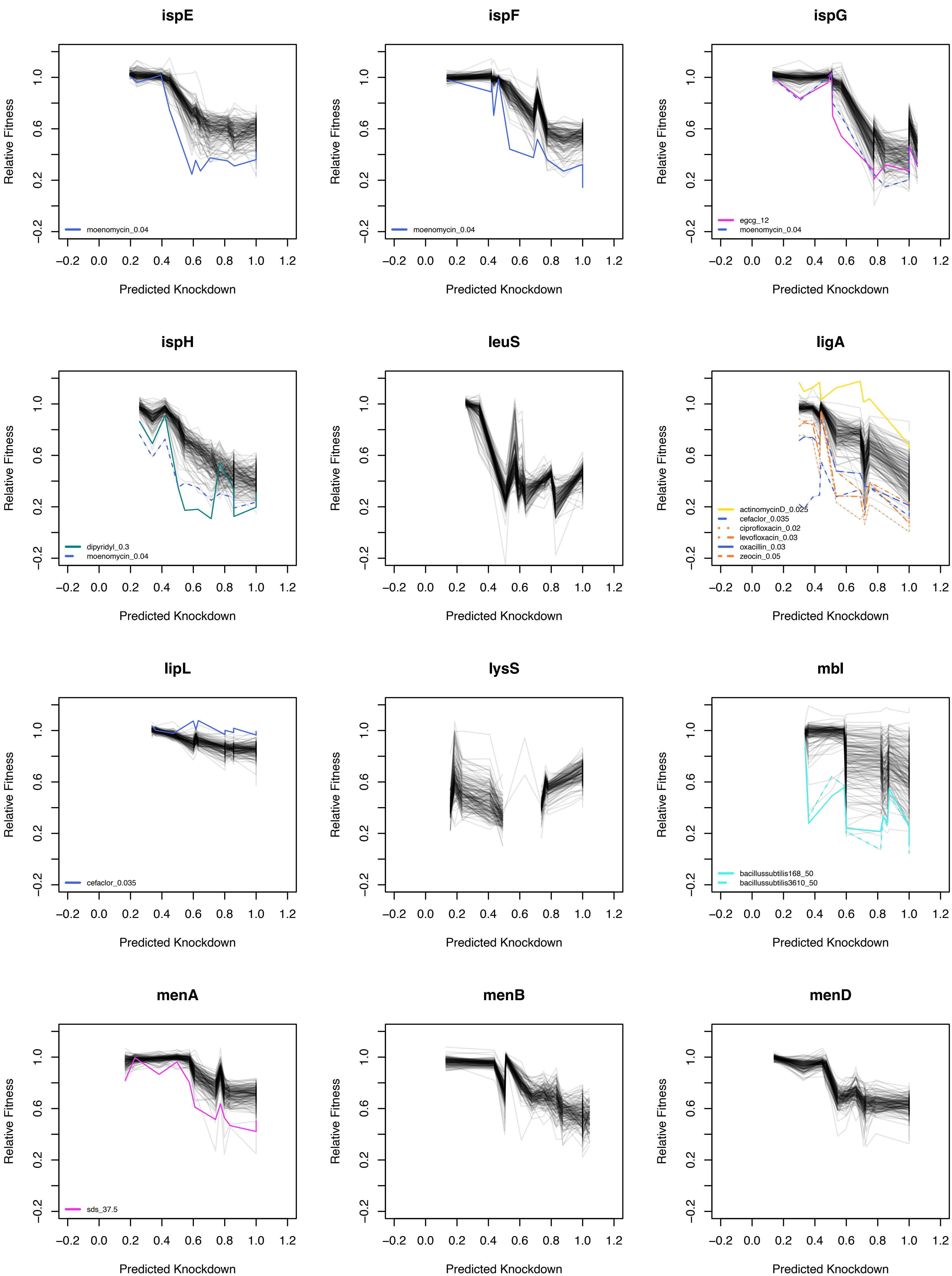

ispE
Relative Fitness
Predicted Knockdown
moenomycin_0.04
ispF
Relative Fitness
Predicted Knockdown
moenomycin_0.04
ispG
Relative Fitness
Predicted Knockdown
egcg_12
moenomycin_0.04
ispH
Relative Fitness
Predicted Knockdown
dipyridyl_0.3
moenomycin_0.04
leuS
Relative Fitness
Predicted Knockdown
ligA
Relative Fitness
Predicted Knockdown
actinomycinD_0.025
cefaclor_0.035
ciprofloxacin_0.02
levofloxacin_0.03
oxacillin_0.03
zeocin_0.05
lipL
Relative Fitness
Predicted Knockdown
cefaclor_0.035
lysS
Relative Fitness
Predicted Knockdown
mbl
Relative Fitness
Predicted Knockdown
bacillussubtilis168_50
bacillussubtilis3610_50
menA
Relative Fitness
Predicted Knockdown
sds_37.5
menB
Relative Fitness
Predicted Knockdown
menD
Relative Fitness
Predicted Knockdown

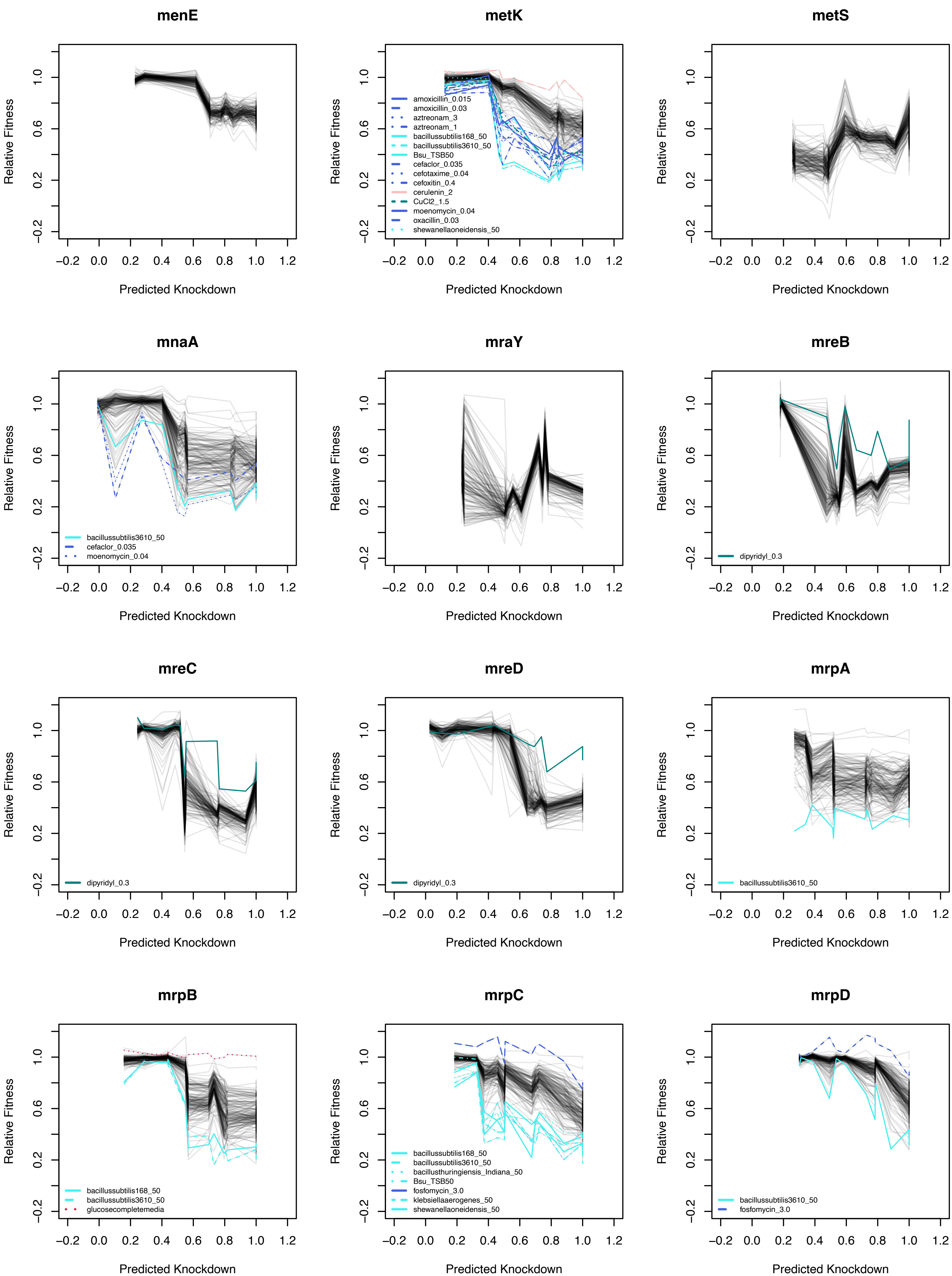

menE
metK
amoxicillin_0.015
amoxicillin_0.03
aztreonam_3
aztreonam_1
bacillussubtilis168_50
bacillussubtilis3610_50
Bsu_TSB50
cefaclor_0.035
cefotaxime_0.04
cefoxitin_0.4
cerulenin_2
CuCl2_1.5
moenomycin_0.04
oxacillin_0.03
shewanellaoneidensis_50
metS
mnaA
bacillussubtilis3610_50
cefaclor_0.035
moenomycin_0.04
mraY
mreB
dipyridyl_0.3
mreC
dipyridyl_0.3
mreD
dipyridyl_0.3
mrpA
bacillussubtilis3610_50
mrpB
bacillussubtilis168_50
bacillussubtilis3610_50
glucosecompletemedia
mrpC
bacillussubtilis168_50
bacillussubtilis3610_50
bacillusthuringiensis_Indiana_50
Bsu_TSB50
fosfomycin_3.0
klebsiellaaerogenes_50
shewanellaoneidensis_50
mrpD
bacillussubtilis3610_50
fosfomycin_3.0
Relative Fitness
Predicted Knockdown

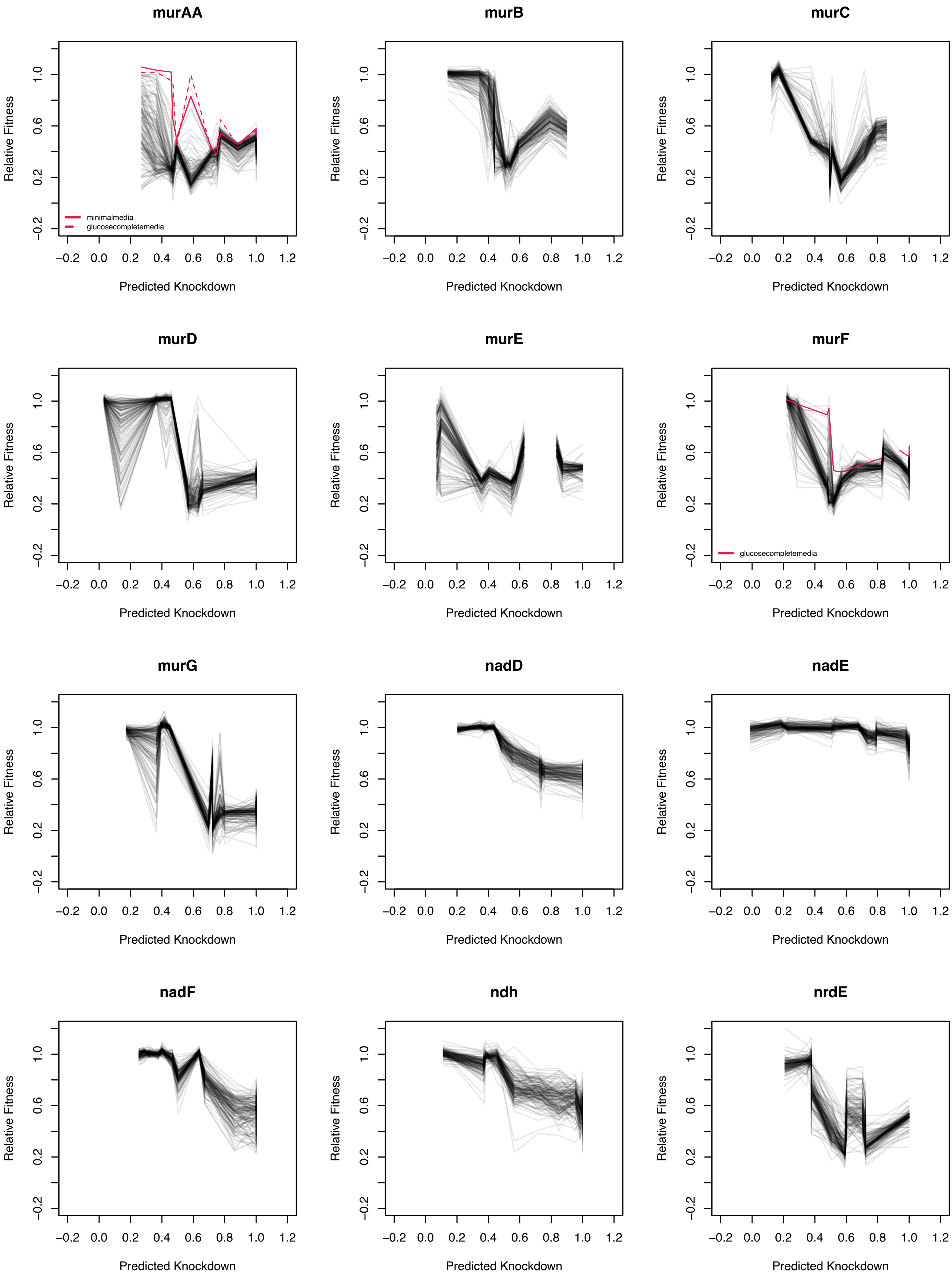
murAA
murB
murC
murD
murE
murF
murG
nadD
nadE
nadF
ndh
nrdE
Relative Fitness
Predicted Knockdown
minimalmedia
glucosecompletemedia
-0.2
0.0
0.2
0.4
0.6
0.8
1.0
1.2

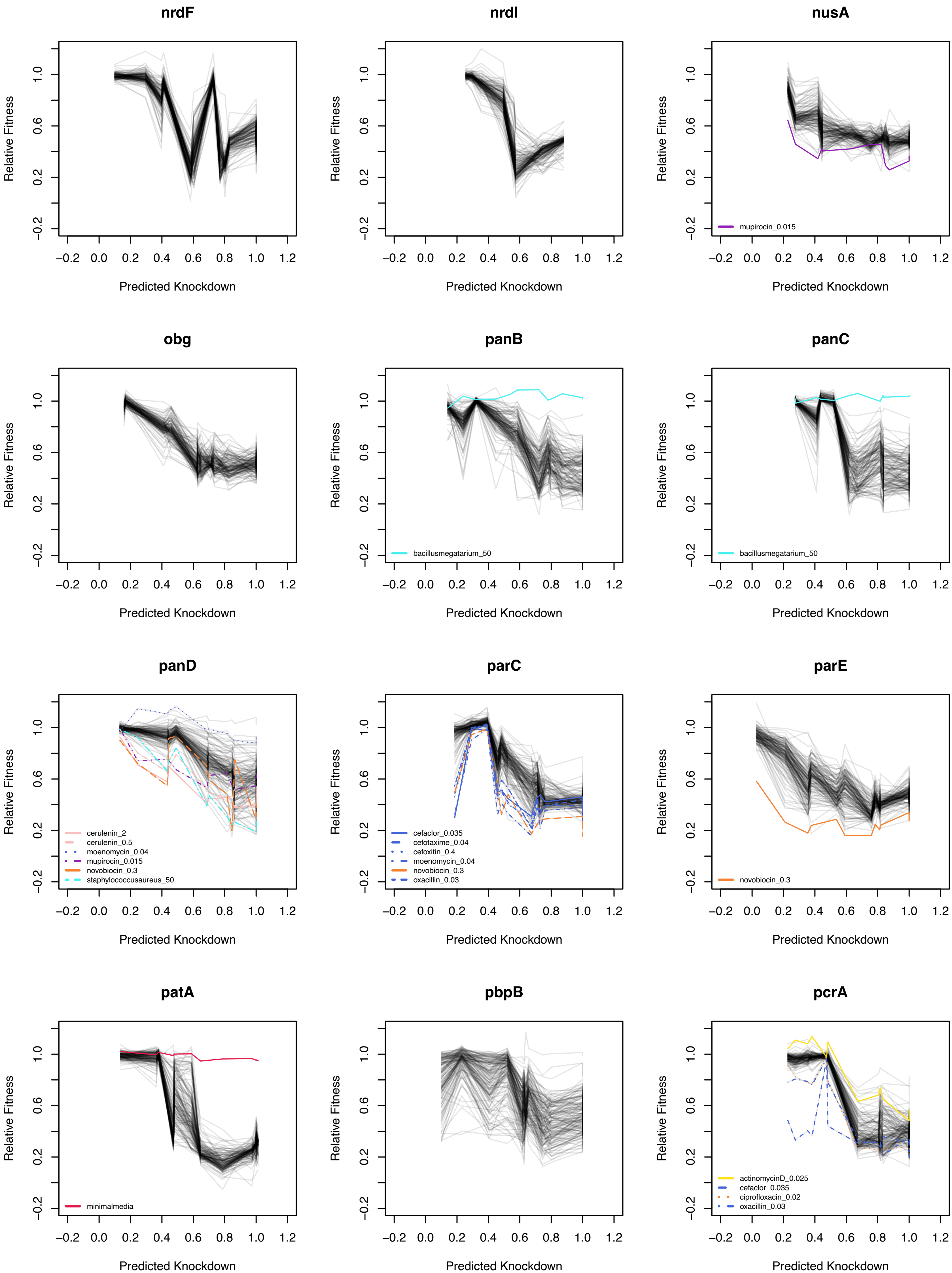

nrdF
nrdI
nusA
obg
panB
panC
panD
parC
parE
patA
pbpB
pcrA
Relative Fitness
Predicted Knockdown
-0.2
0.2
0.6
1.0
-0.2
0.0
0.2
0.4
0.6
0.8
1.0
1.2
mupirocin_0.015
bacillusmegatarium_50
bacillusmegatarium_50
cerulenin_2
cerulenin_0.5
moenomycin_0.04
mupirocin_0.015
novobiocin_0.3
staphylococcusaureus_50
cefaclor_0.035
cefotaxime_0.04
cefoxitin_0.4
moenomycin_0.04
novobiocin_0.3
oxacillin_0.03
novobiocin_0.3
minimalmedia
actinomycinD_0.025
cefaclor_0.035
ciprofloxacin_0.02
oxacillin_0.03

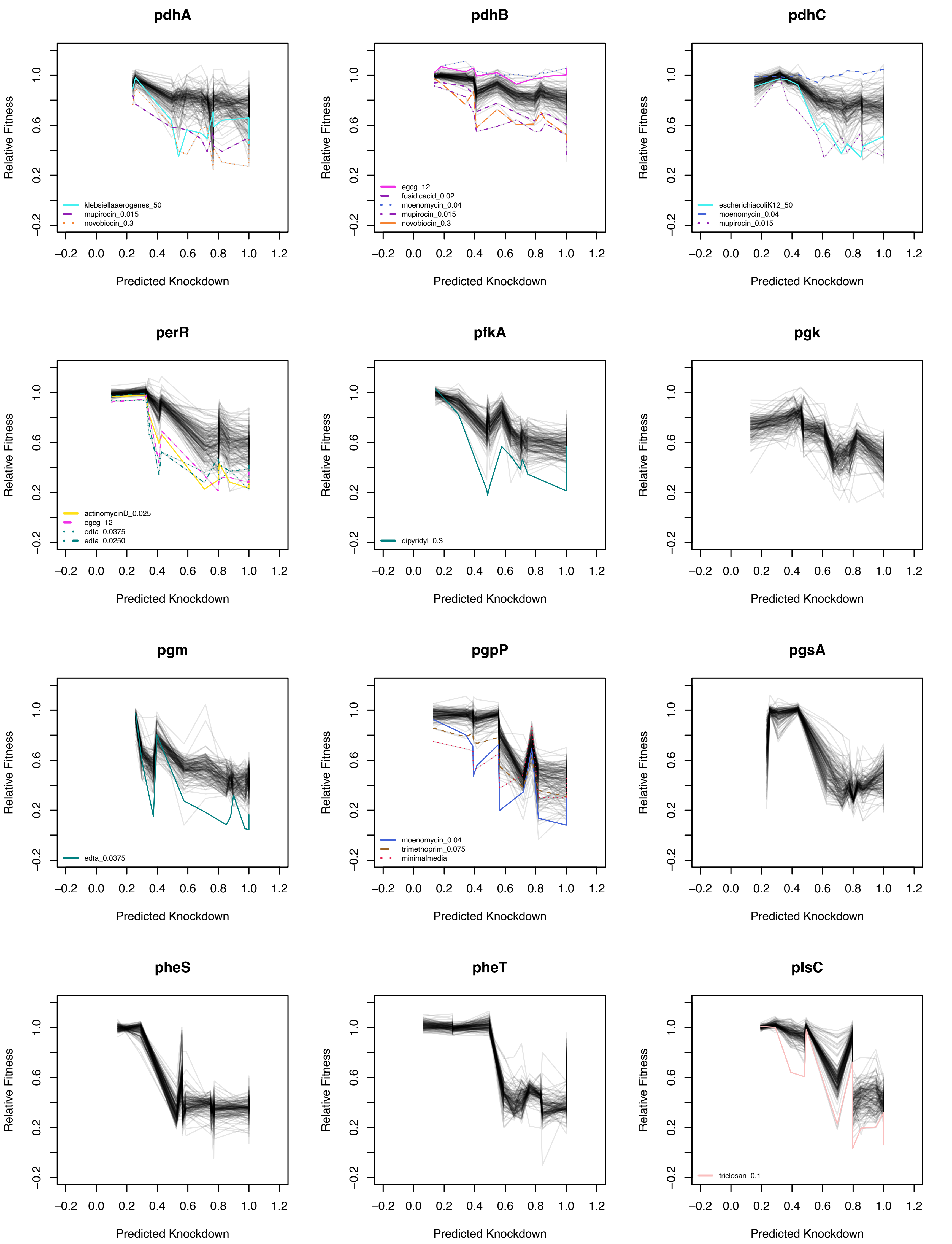
pdhA
Relative Fitness
Predicted Knockdown
klebsiellaaerogenes_50
mupirocin_0.015
novobiocin_0.3
pdhB
Relative Fitness
Predicted Knockdown
egcg_12
fusidicacid_0.02
moenomycin_0.04
mupirocin_0.015
novobiocin_0.3
pdhC
Relative Fitness
Predicted Knockdown
escherichiacoliK12_50
moenomycin_0.04
mupirocin_0.015
perR
Relative Fitness
Predicted Knockdown
actinomycinD_0.025
egcg_12
edta_0.0375
edta_0.0250
pfkA
Relative Fitness
Predicted Knockdown
dipyridyl_0.3
pgk
Relative Fitness
Predicted Knockdown
pgm
Relative Fitness
Predicted Knockdown
edta_0.0375
pgpP
Relative Fitness
Predicted Knockdown
moenomycin_0.04
trimethoprim_0.075
minimalmedia
pgsA
Relative Fitness
Predicted Knockdown
pheS
Relative Fitness
Predicted Knockdown
pheT
Relative Fitness
Predicted Knockdown
plsC
Relative Fitness
Predicted Knockdown
triclosan_0.1_

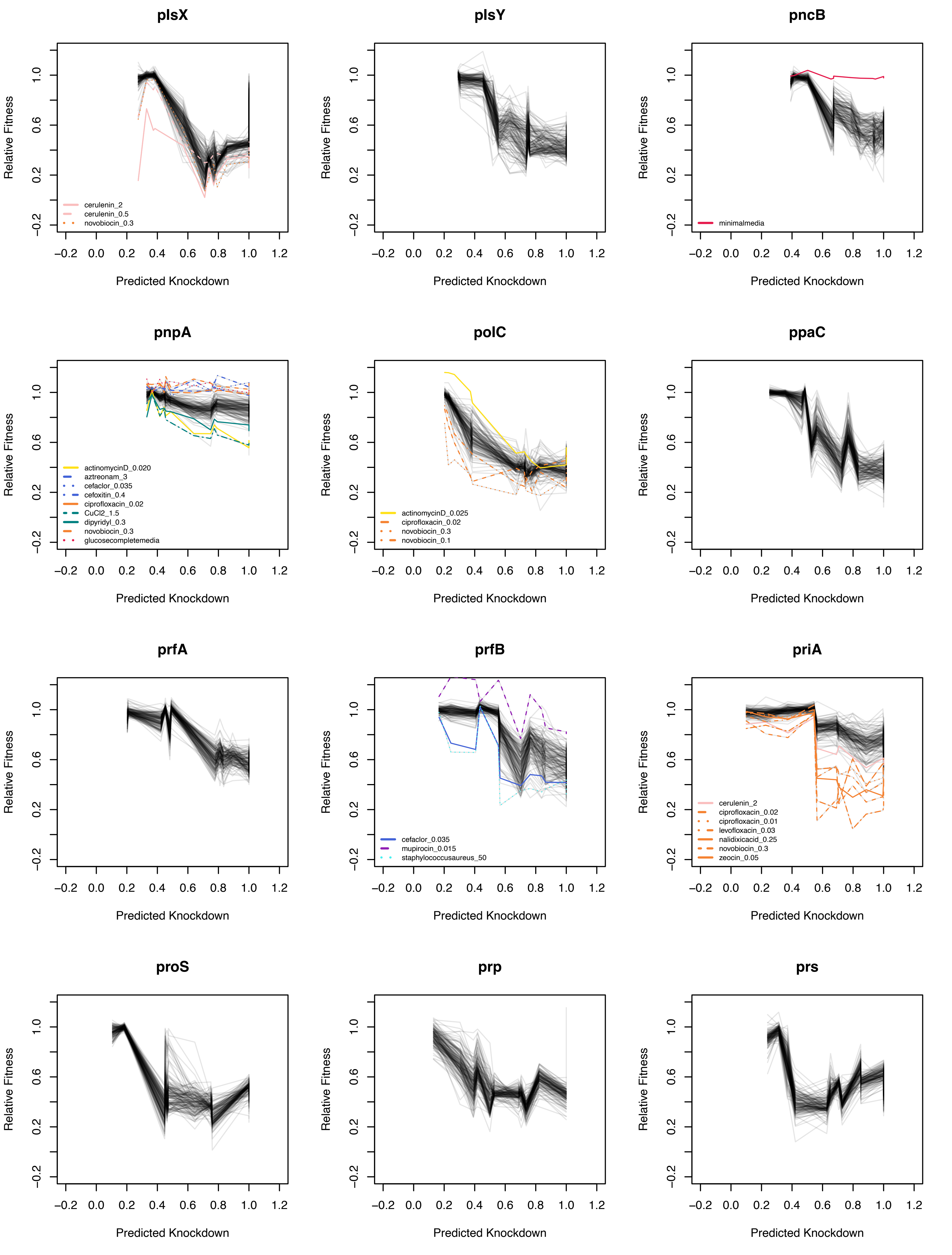
plsX
Relative Fitness
Predicted Knockdown
cerulenin_2
cerulenin_0.5
novobiocin_0.3
plsY
Relative Fitness
Predicted Knockdown
pncB
Relative Fitness
Predicted Knockdown
minimalmedia
pnpA
Relative Fitness
Predicted Knockdown
actinomycinD_0.020
aztreonam_3
cefaclor_0.035
cefoxitin_0.4
ciprofloxacin_0.02
CuCl2_1.5
dipyridyl_0.3
novobiocin_0.3
glucosecompletemedia
polC
Relative Fitness
Predicted Knockdown
actinomycinD_0.025
ciprofloxacin_0.02
novobiocin_0.3
novobiocin_0.1
ppaC
Relative Fitness
Predicted Knockdown
prfA
Relative Fitness
Predicted Knockdown
prfB
Relative Fitness
Predicted Knockdown
cefaclor_0.035
mupirocin_0.015
staphylococcusaureus_50
priA
Relative Fitness
Predicted Knockdown
cerulenin_2
ciprofloxacin_0.02
ciprofloxacin_0.01
levofloxacin_0.03
nalidixicacid_0.25
novobiocin_0.3
zeocin_0.05
proS
Relative Fitness
Predicted Knockdown
prp
Relative Fitness
Predicted Knockdown
prs
Relative Fitness
Predicted Knockdown

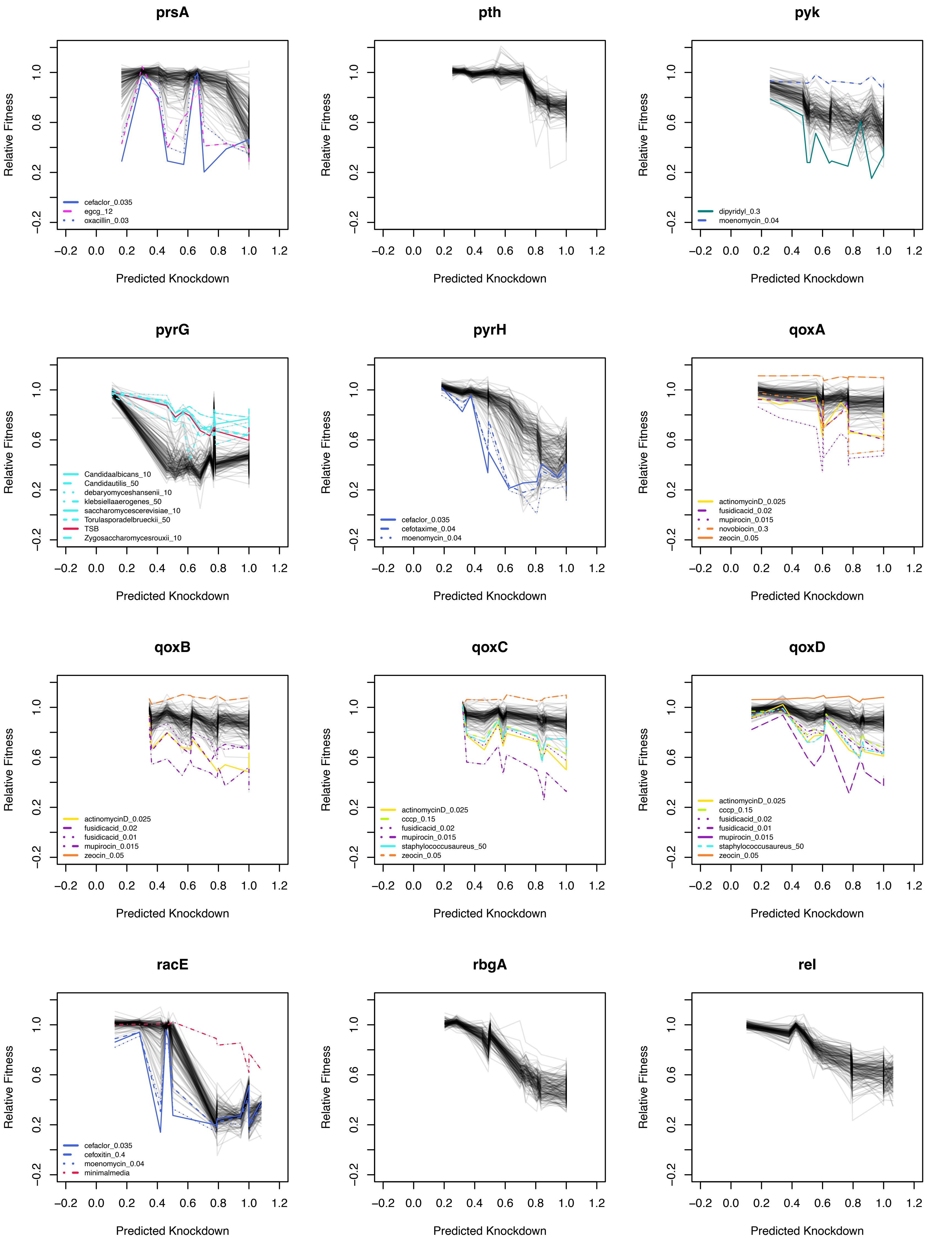
prsA
Relative Fitness
Predicted Knockdown
cefaclor_0.035
egcg_12
oxacillin_0.03
pth
Relative Fitness
Predicted Knockdown
pyk
Relative Fitness
Predicted Knockdown
dipyridyl_0.3
moenomycin_0.04
pyrG
Relative Fitness
Predicted Knockdown
Candidaalbicans_10
Candidautilis_50
debaryomyceshansenii_10
klebsiellaaerogenes_50
saccharomycescerevisiae_10
Torulasporadelbrueckii_50
TSB
Zygosaccharomycesrouxii_10
pyrH
Relative Fitness
Predicted Knockdown
cefaclor_0.035
cefotaxime_0.04
moenomycin_0.04
qoxA
Relative Fitness
Predicted Knockdown
actinomycinD_0.025
fusidicacid_0.02
mupirocin_0.015
novobiocin_0.3
zeocin_0.05
qoxB
Relative Fitness
Predicted Knockdown
actinomycinD_0.025
fusidicacid_0.02
fusidicacid_0.01
mupirocin_0.015
zeocin_0.05
qoxC
Relative Fitness
Predicted Knockdown
actinomycinD_0.025
cccp_0.15
fusidicacid_0.02
mupirocin_0.015
staphylococcusaureus_50
zeocin_0.05
qoxD
Relative Fitness
Predicted Knockdown
actinomycinD_0.025
cccp_0.15
fusidicacid_0.02
fusidicacid_0.01
mupirocin_0.015
staphylococcusaureus_50
zeocin_0.05
racE
Relative Fitness
Predicted Knockdown
cefaclor_0.035
cefoxitin_0.4
moenomycin_0.04
minimalmedia
rbgA
Relative Fitness
Predicted Knockdown
rel
Relative Fitness
Predicted Knockdown

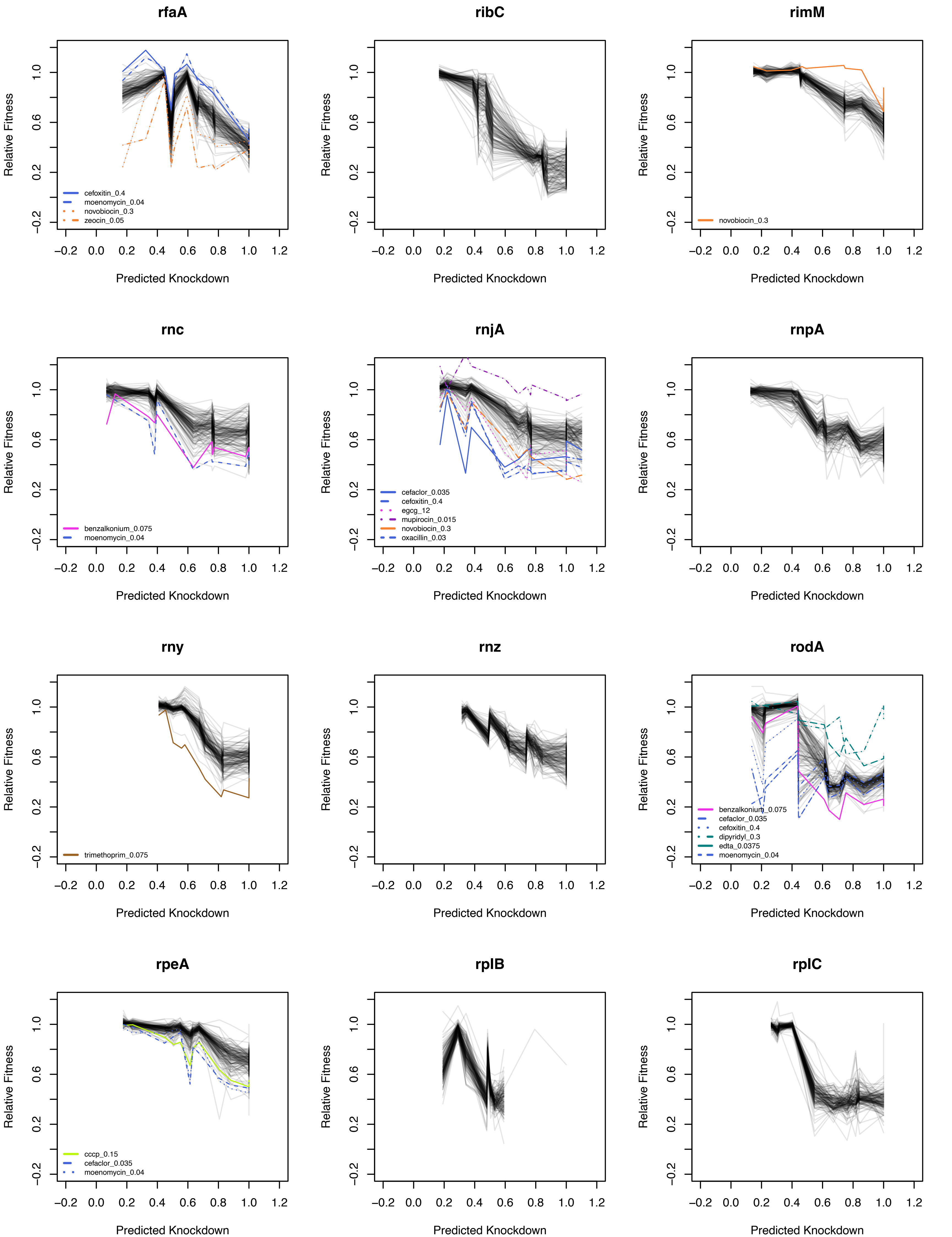
rfaA
Relative Fitness
Predicted Knockdown
cefoxitin_0.4
moenomycin_0.04
novobiocin_0.3
zeocin_0.05
ribC
Relative Fitness
Predicted Knockdown
rimM
Relative Fitness
Predicted Knockdown
novobiocin_0.3
rnc
Relative Fitness
Predicted Knockdown
benzalkonium_0.075
moenomycin_0.04
rnjA
Relative Fitness
Predicted Knockdown
cefaclor_0.035
cefoxitin_0.4
egcg_12
mupirocin_0.015
novobiocin_0.3
oxacillin_0.03
rnpA
Relative Fitness
Predicted Knockdown
rny
Relative Fitness
Predicted Knockdown
trimethoprim_0.075
rnz
Relative Fitness
Predicted Knockdown
rodA
Relative Fitness
Predicted Knockdown
benzalkonium_0.075
cefaclor_0.035
cefoxitin_0.4
dipyridyl_0.3
edta_0.0375
moenomycin_0.04
rpeA
Relative Fitness
Predicted Knockdown
cccp_0.15
cefaclor_0.035
moenomycin_0.04
rplB
Relative Fitness
Predicted Knockdown
rplC
Relative Fitness
Predicted Knockdown

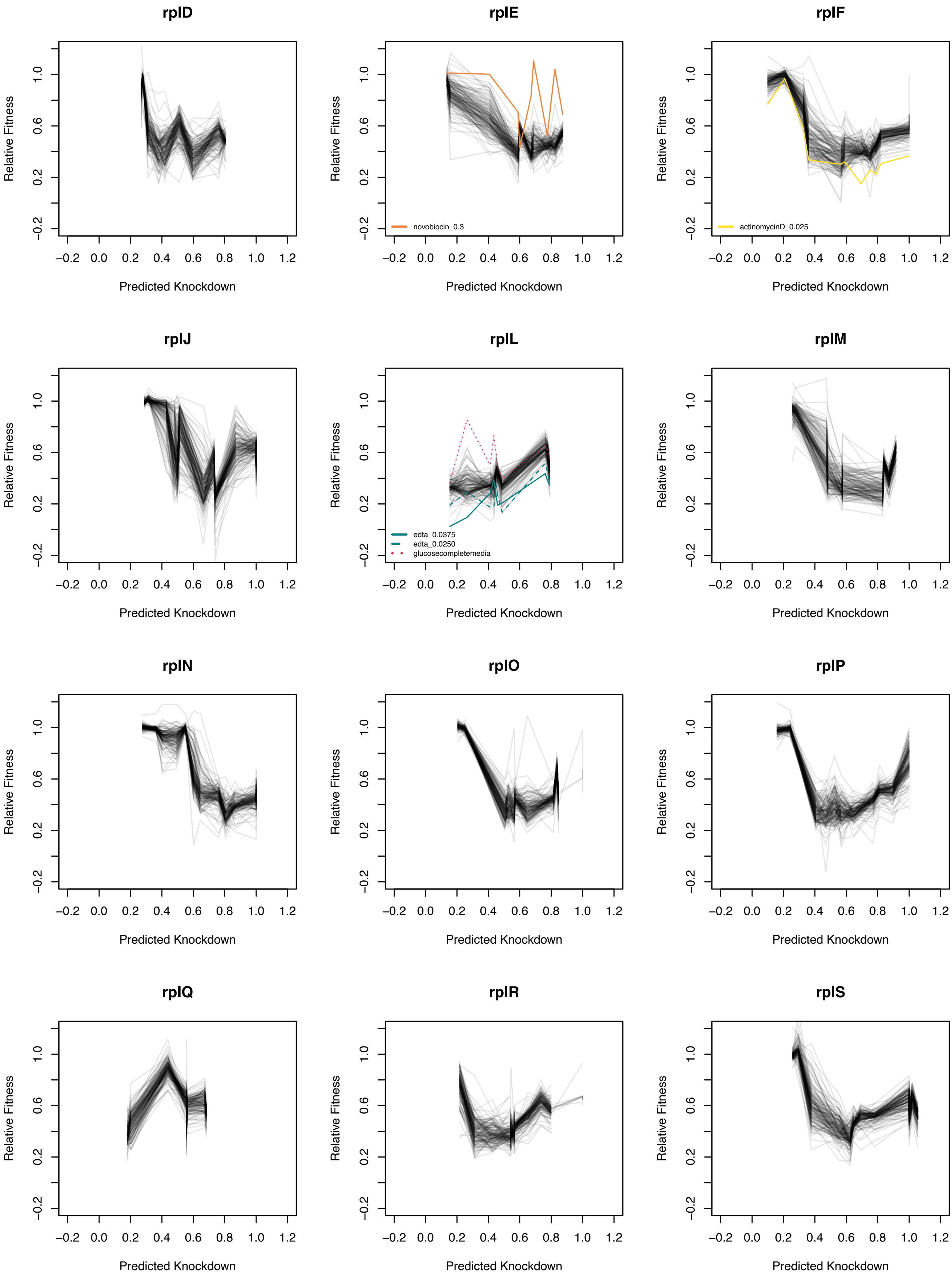
rplD
rplE
rplF
rplJ
rplL
rplM
rplN
rplO
rplP
rplQ
rplR
rplS
Relative Fitness
Predicted Knockdown
novobiocin_0.3
actinomycinD_0.025
edta_0.0375
edta_0.0250
glucosecompletemedia

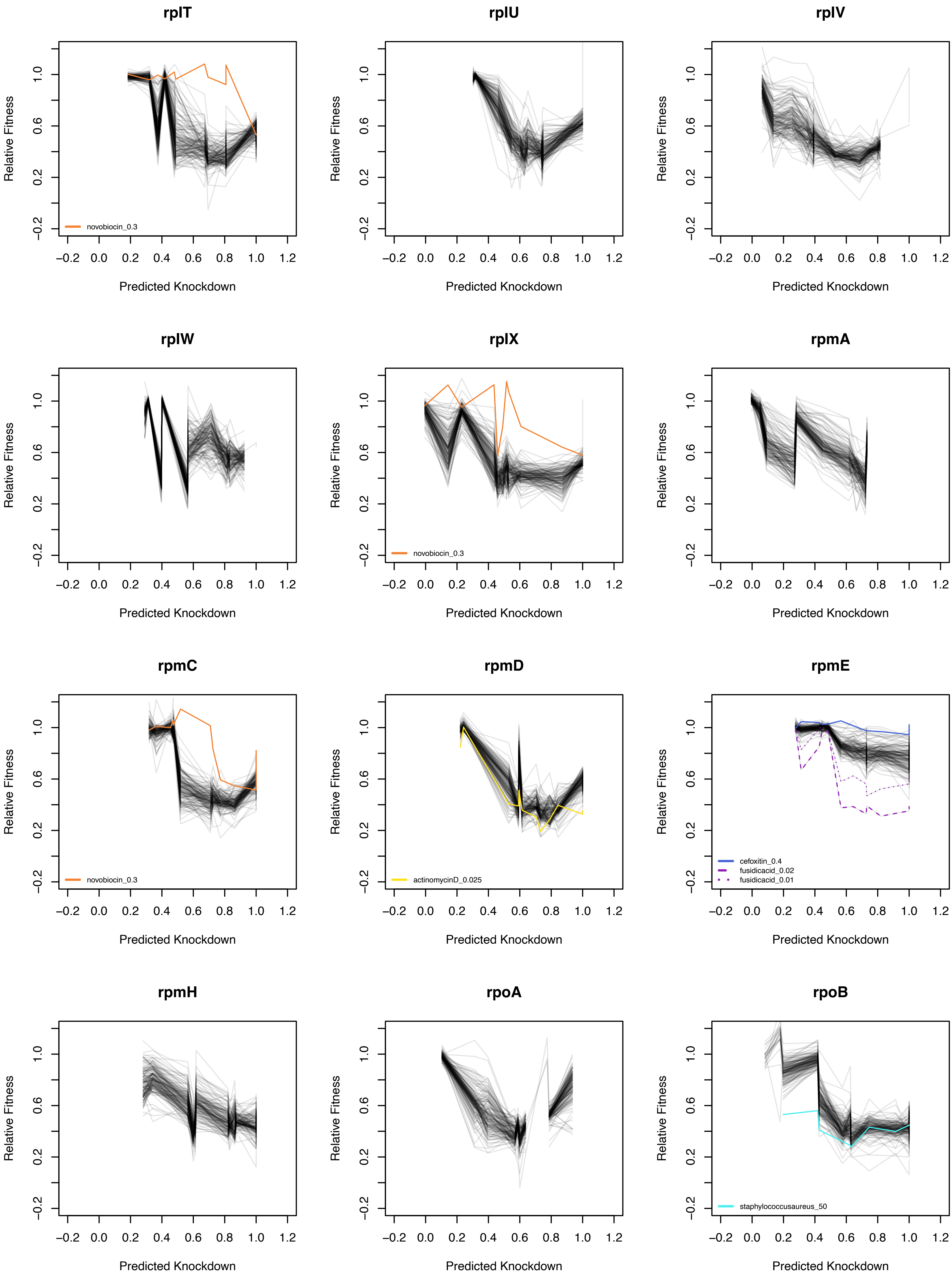
rplT
Relative Fitness
-0.2 0.2 0.6 1.0
-0.2 0.0 0.2 0.4 0.6 0.8 1.0 1.2
Predicted Knockdown
novobiocin_0.3
rplU
Relative Fitness
-0.2 0.2 0.6 1.0
-0.2 0.0 0.2 0.4 0.6 0.8 1.0 1.2
Predicted Knockdown
rplV
Relative Fitness
-0.2 0.2 0.6 1.0
-0.2 0.0 0.2 0.4 0.6 0.8 1.0 1.2
Predicted Knockdown
rplW
Relative Fitness
-0.2 0.2 0.6 1.0
-0.2 0.0 0.2 0.4 0.6 0.8 1.0 1.2
Predicted Knockdown
rplX
Relative Fitness
-0.2 0.2 0.6 1.0
-0.2 0.0 0.2 0.4 0.6 0.8 1.0 1.2
Predicted Knockdown
novobiocin_0.3
rpmA
Relative Fitness
-0.2 0.2 0.6 1.0
-0.2 0.0 0.2 0.4 0.6 0.8 1.0 1.2
Predicted Knockdown
rpmC
Relative Fitness
-0.2 0.2 0.6 1.0
-0.2 0.0 0.2 0.4 0.6 0.8 1.0 1.2
Predicted Knockdown
novobiocin_0.3
rpmD
Relative Fitness
-0.2 0.2 0.6 1.0
-0.2 0.0 0.2 0.4 0.6 0.8 1.0 1.2
Predicted Knockdown
actinomycinD_0.025
rpmE
Relative Fitness
-0.2 0.2 0.6 1.0
-0.2 0.0 0.2 0.4 0.6 0.8 1.0 1.2
Predicted Knockdown
cefoxitin_0.4
fusidicacid_0.02
fusidicacid_0.01
rpmH
Relative Fitness
-0.2 0.2 0.6 1.0
-0.2 0.0 0.2 0.4 0.6 0.8 1.0 1.2
Predicted Knockdown
rpoA
Relative Fitness
-0.2 0.2 0.6 1.0
-0.2 0.0 0.2 0.4 0.6 0.8 1.0 1.2
Predicted Knockdown
rpoB
Relative Fitness
-0.2 0.2 0.6 1.0
-0.2 0.0 0.2 0.4 0.6 0.8 1.0 1.2
Predicted Knockdown
staphylococcusaureus_50

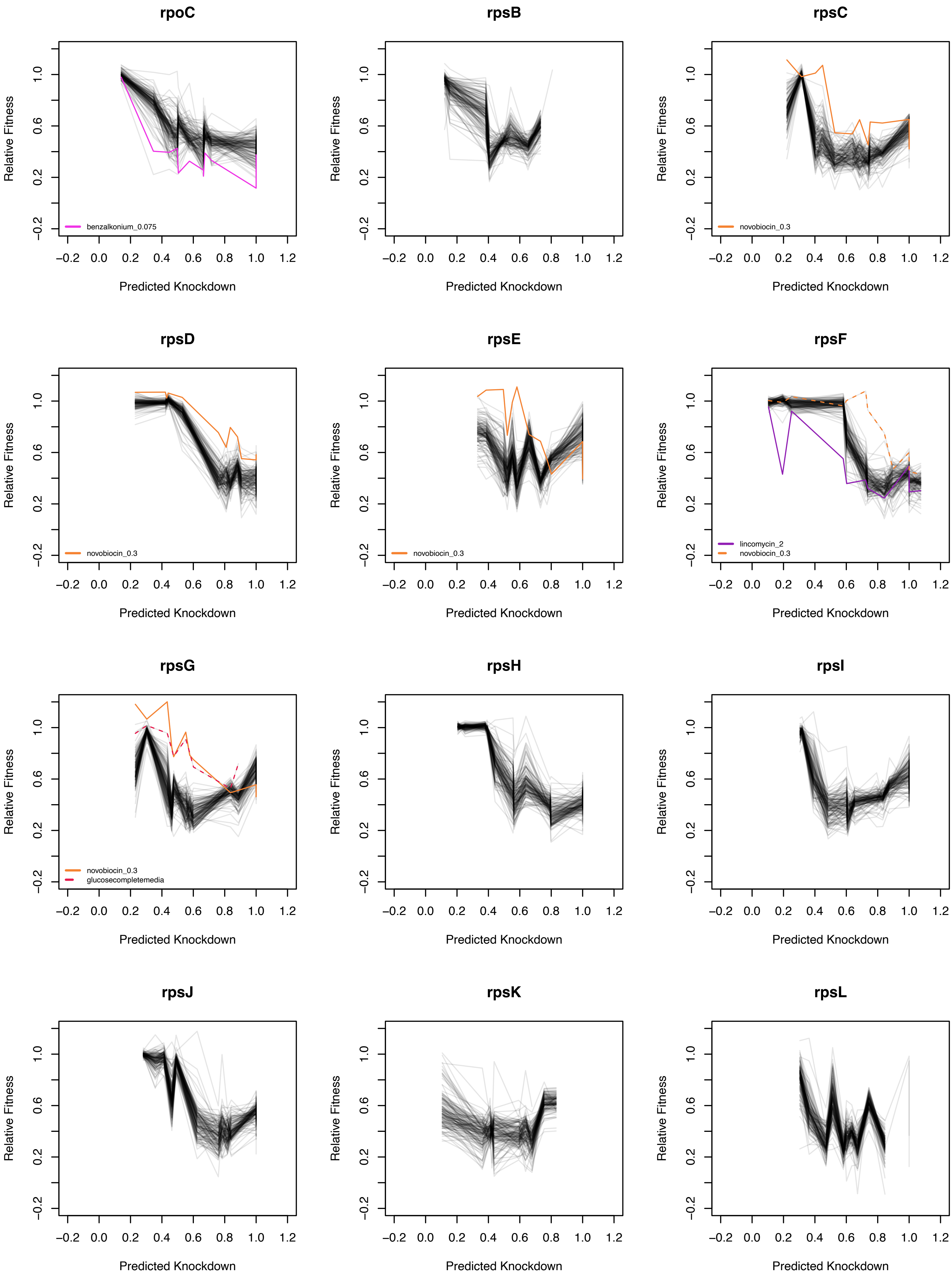
rpoC
benzalkonium_0.075
rpsB
rpsC
novobiocin_0.3
rpsD
novobiocin_0.3
rpsE
novobiocin_0.3
rpsF
lincomycin_2
novobiocin_0.3
rpsG
novobiocin_0.3
glucosecompletemedia
rpsH
rpsI
rpsJ
rpsK
rpsL
Relative Fitness
Predicted Knockdown

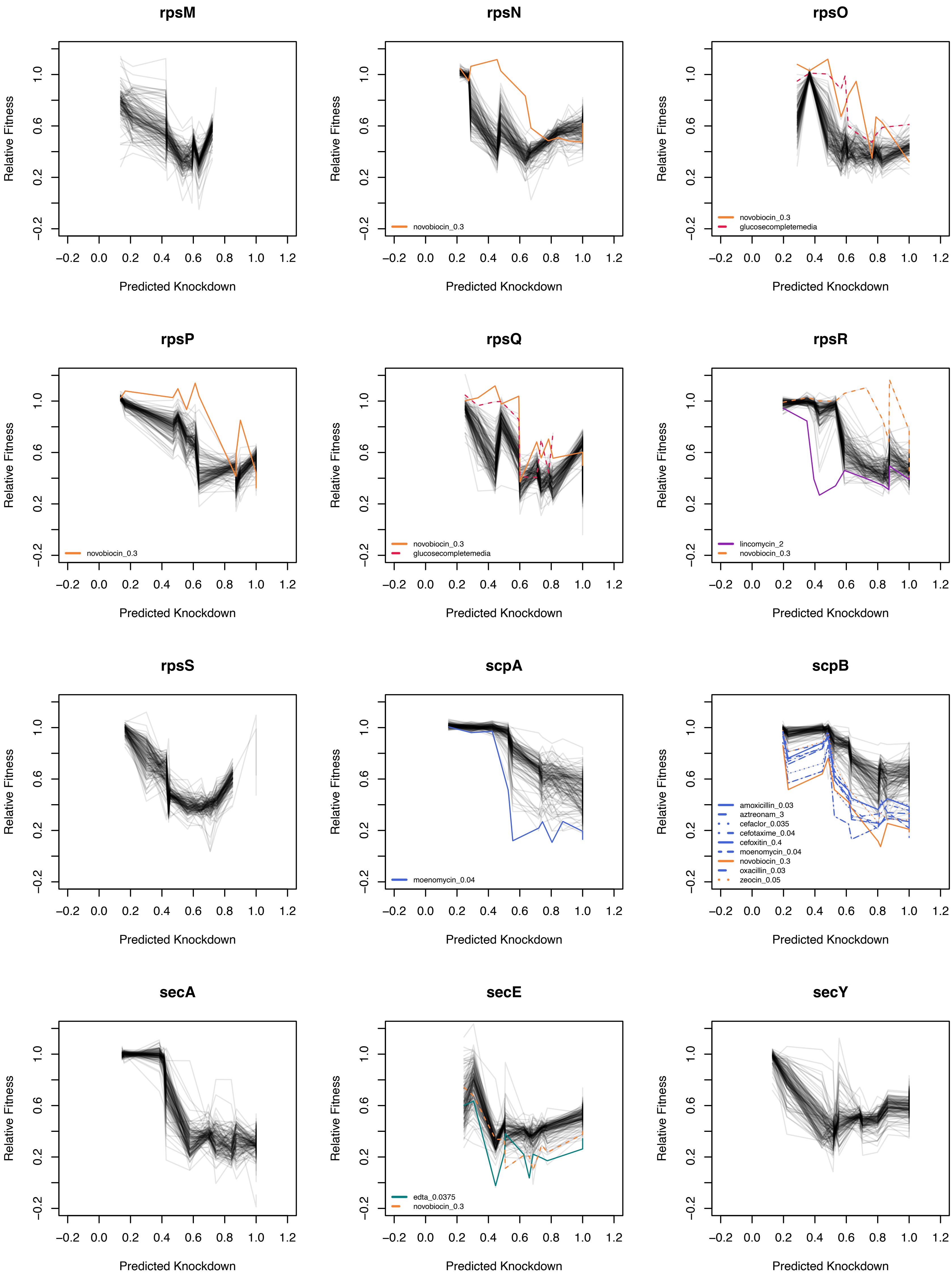
rpsM
rpsN
novobiocin_0.3
rpsO
novobiocin_0.3
glucosecompletemedia
rpsP
novobiocin_0.3
rpsQ
novobiocin_0.3
glucosecompletemedia
rpsR
lincomycin_2
novobiocin_0.3
rpsS
scpA
moenomycin_0.04
scpB
amoxicillin_0.03
aztreonam_3
cefaclor_0.035
cefotaxime_0.04
cefoxitin_0.4
moenomycin_0.04
novobiocin_0.3
oxacillin_0.03
zeocin_0.05
secA
secE
edta_0.0375
novobiocin_0.3
secY
Relative Fitness
Predicted Knockdown
−0.2 0.2 0.6 1.0
−0.2 0.0 0.2 0.4 0.6 0.8 1.0 1.2

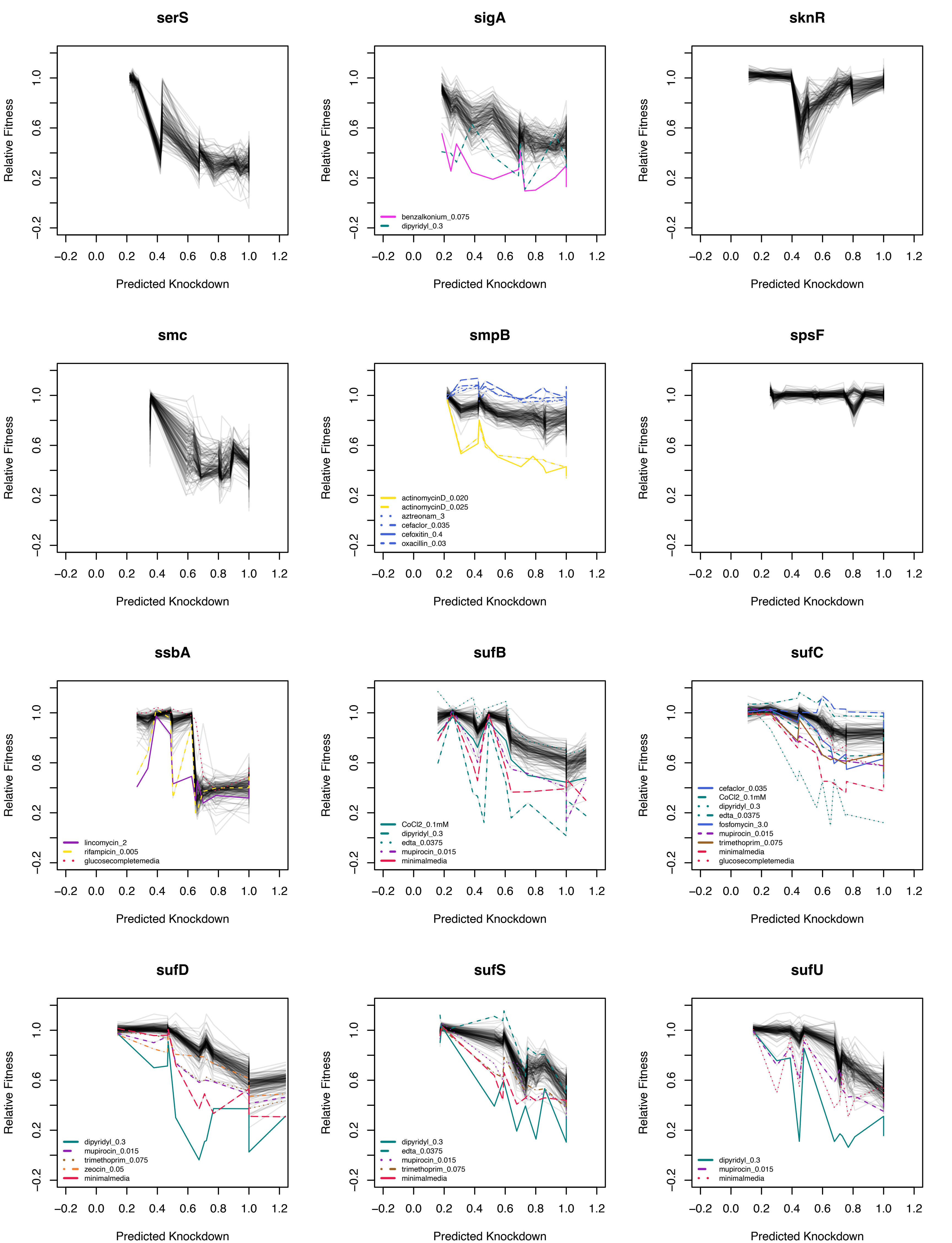

serS
sigA
benzalkonium_0.075
dipyridyl_0.3
sknR
smc
smpB
actinomycinD_0.020
actinomycinD_0.025
aztreonam_3
cefaclor_0.035
cefoxitin_0.4
oxacillin_0.03
spsF
ssbA
lincomycin_2
rifampicin_0.005
glucosecompletemedia
sufB
CoCl2_0.1mM
dipyridyl_0.3
edta_0.0375
mupirocin_0.015
minimalmedia
sufC
cefaclor_0.035
CoCl2_0.1mM
dipyridyl_0.3
edta_0.0375
fosfomycin_3.0
mupirocin_0.015
trimethoprim_0.075
minimalmedia
glucosecompletemedia
sufD
dipyridyl_0.3
mupirocin_0.015
trimethoprim_0.075
zeocin_0.05
minimalmedia
sufS
dipyridyl_0.3
edta_0.0375
mupirocin_0.015
trimethoprim_0.075
minimalmedia
sufU
dipyridyl_0.3
mupirocin_0.015
minimalmedia
Relative Fitness
Predicted Knockdown
−0.2 0.0 0.2 0.4 0.6 0.8 1.0 1.2

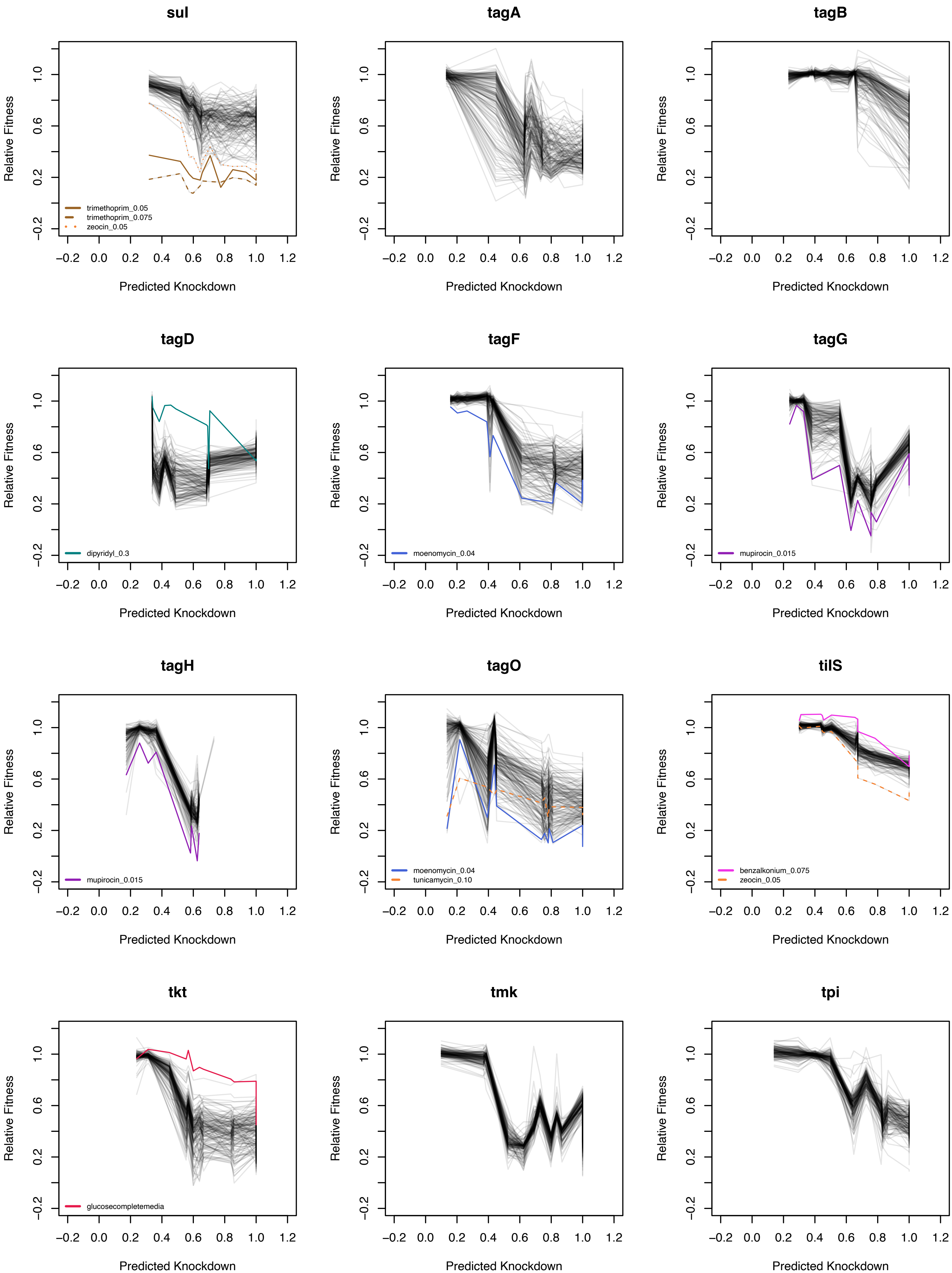
sul
trimethoprim_0.05
trimethoprim_0.075
zeocin_0.05
tagA
tagB
tagD
dipyridyl_0.3
tagF
moenomycin_0.04
tagG
mupirocin_0.015
tagH
mupirocin_0.015
tagO
moenomycin_0.04
tunicamycin_0.10
tilS
benzalkonium_0.075
zeocin_0.05
tkt
glucosecompletemedia
tmk
tpi
Relative Fitness
Predicted Knockdown

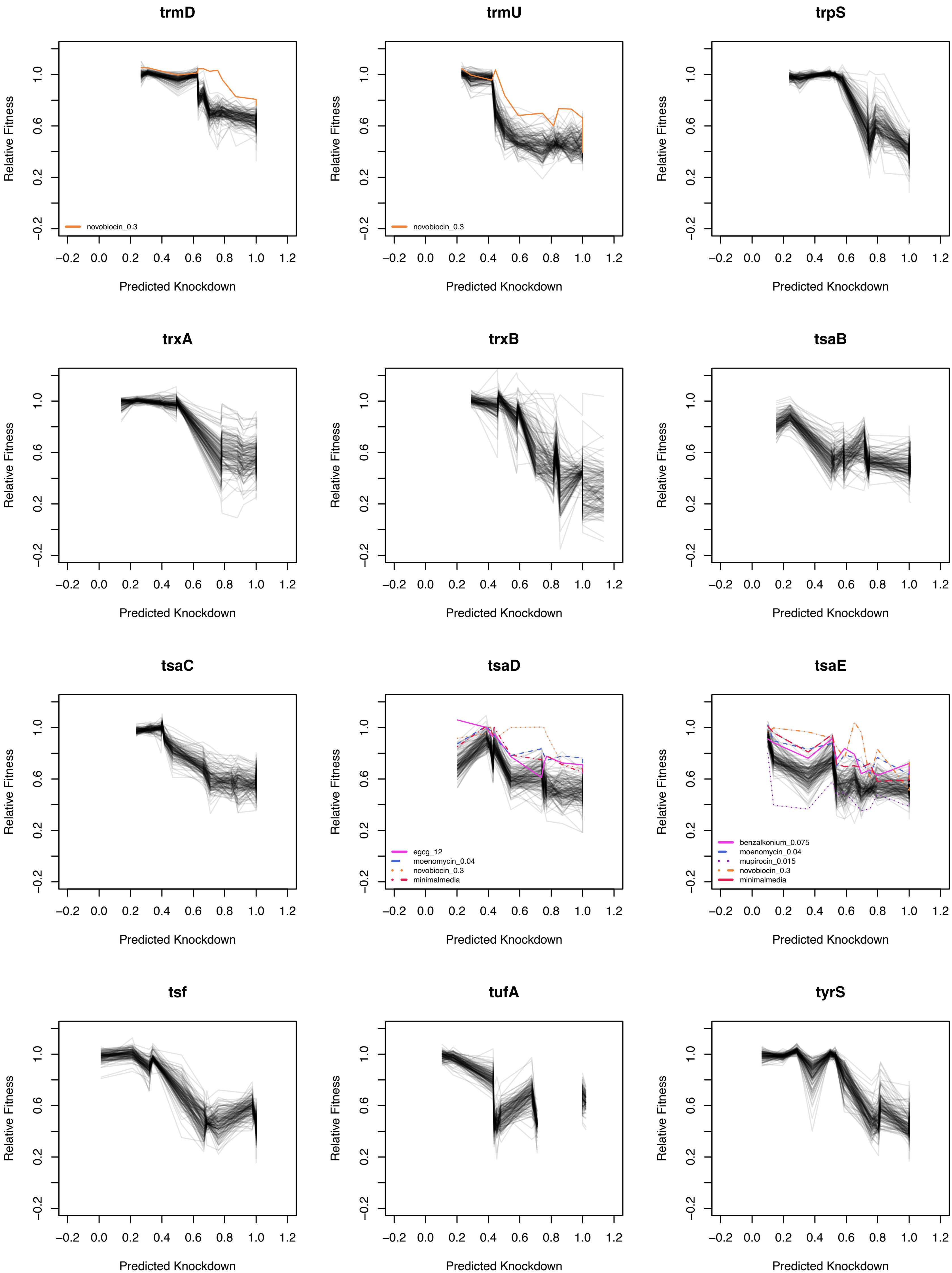
trmD
novobiocin_0.3
trmU
novobiocin_0.3
trpS
trxA
trxB
tsaB
tsaC
tsaD
egcg_12
moenomycin_0.04
novobiocin_0.3
minimalmedia
tsaE
benzalkonium_0.075
moenomycin_0.04
mupirocin_0.015
novobiocin_0.3
minimalmedia
tsf
tufA
tyrS
Relative Fitness
-0.2
0.2
0.6
1.0
Predicted Knockdown
-0.2
0.0
0.2
0.4
0.6
0.8
1.0
1.2

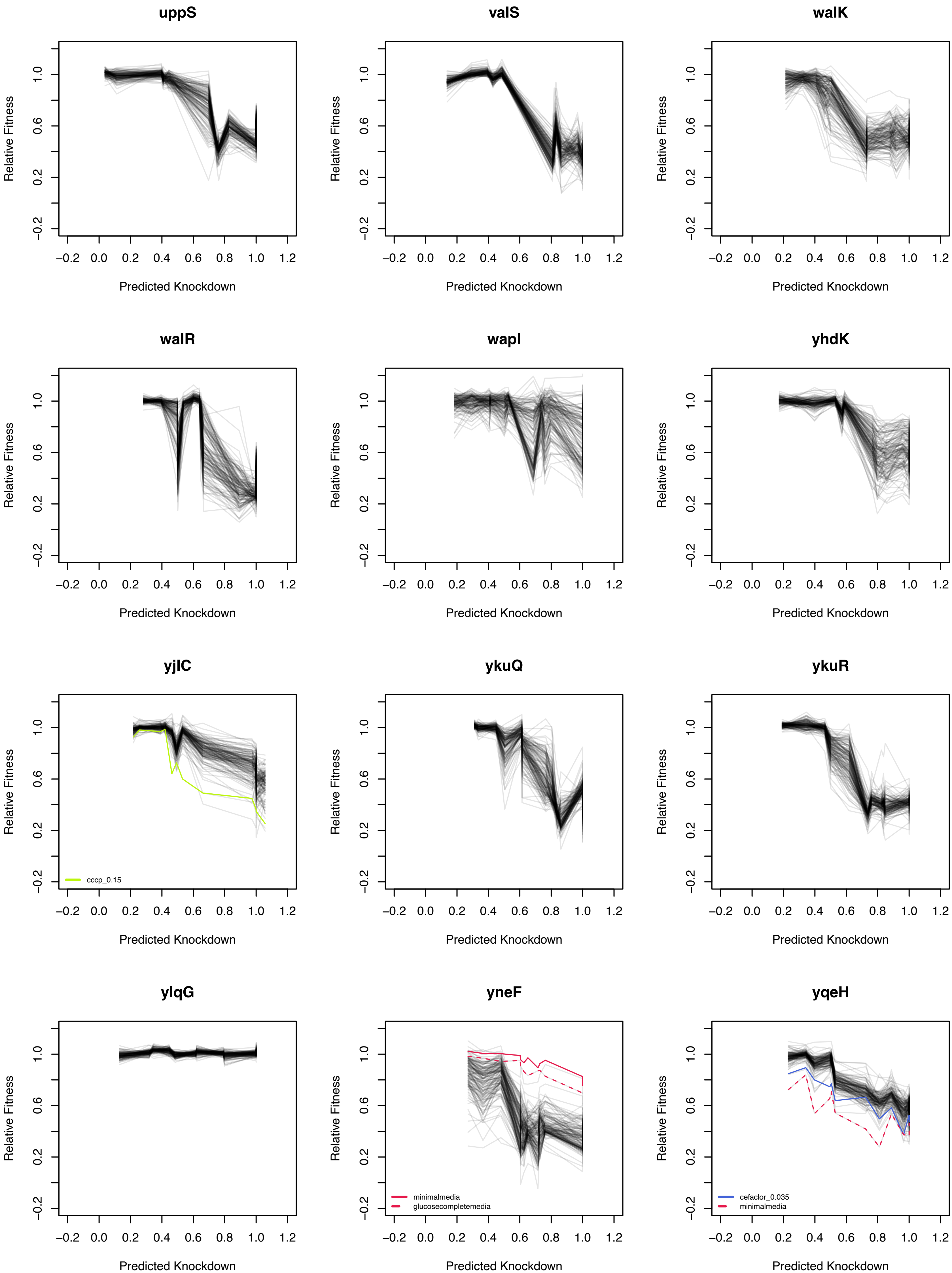

uppS
valS
walK
walR
wapI
yhdK
yjlC
ykuQ
ykuR
ylqG
yneF
yqeH
Relative Fitness
Predicted Knockdown
cccp_0.15
minimalmedia
glucosecompletemedia
cefaclor_0.035
minimalmedia

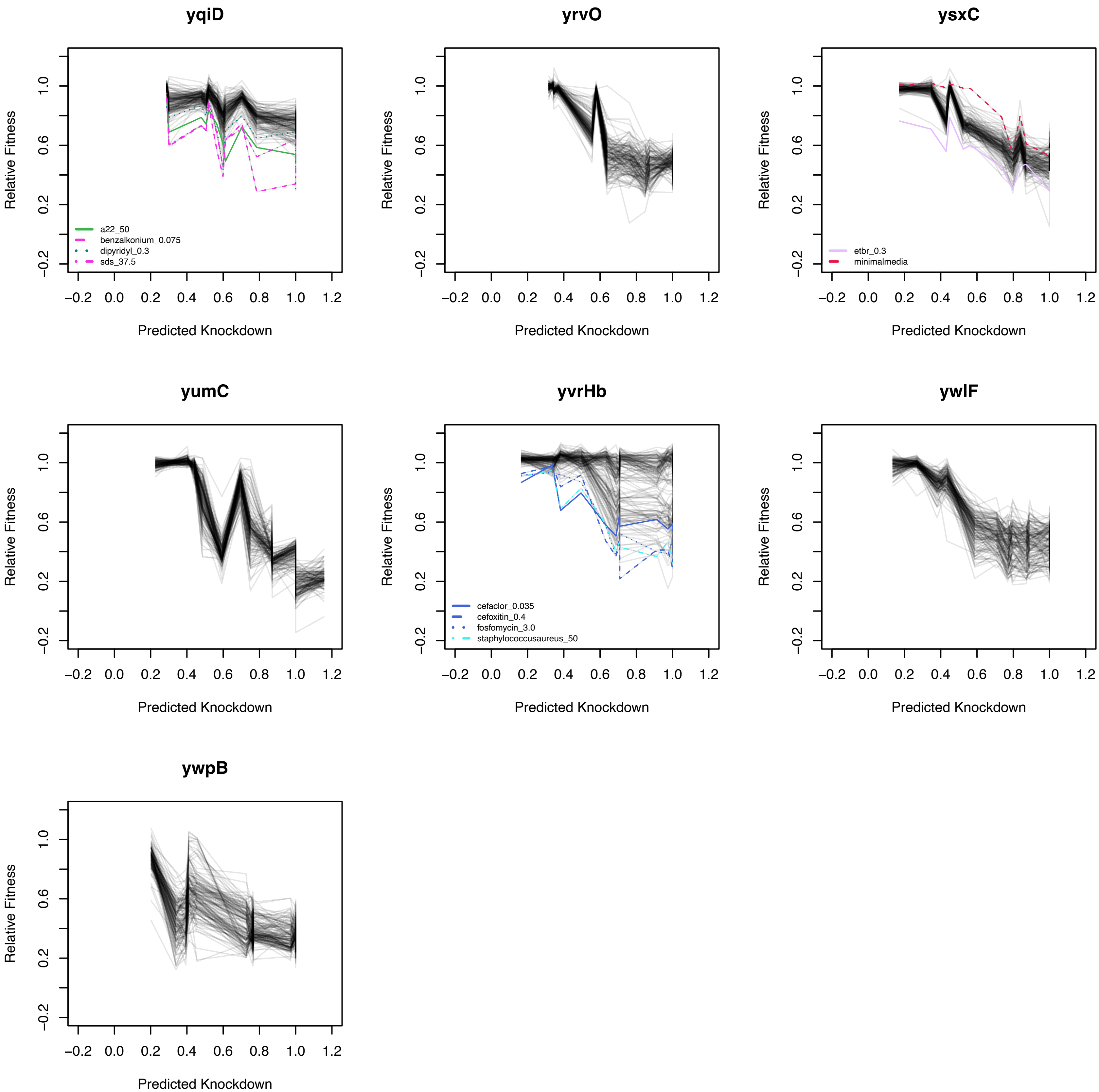
yqiD
a22_50
benzalkonium_0.075
dipyridyl_0.3
sds_37.5
yrvO
ysxC
etbr_0.3
minimalmedia
yumC
yvrHb
cefaclor_0.035
cefoxitin_0.4
fosfomycin_3.0
staphylococcusaureus_50
ywlF
ywpB
Relative Fitness
Predicted Knockdown
-0.2
0.2
0.6
1.0
-0.2
0.0
0.2
0.4
0.6
0.8
1.0
1.2
